# How much does TRPV1 deviate from an ideal MWC-type protein?

**DOI:** 10.1016/j.bpj.2024.04.005

**Published:** 2024-04-06

**Authors:** Shisheng Li, Jie Zheng

**Affiliations:** 1 Department of Physiology and Membrane Biology, University of California at Davis, School of Medicine, Davis, California

## Abstract

Many ion channels are known to behave as an allosteric protein, coupling environmental stimuli captured by specialized sensing domains to the opening of a central pore. The classic Monod-Wyman-Changeux (MWC) model, originally proposed to describe binding of gas molecules to hemoglobin, has been widely used as a framework for analyzing ion channel gating. Here, we address the issue of how accurately the MWC model predicts activation of the capsaicin receptor TRPV1 by vanilloids. Taking advantage of a concatemeric design that makes it possible to lock TRPV1 in states with zero to four bound vanilloid molecules, we showed quantitatively that the overall gating behavior is satisfactorily predicted by the MWC model. There is, however, a small yet detectable subunit position effect: ligand binding to two kitty-corner subunits is 0.3–0.4 kcal/mol more effective in inducing opening than binding to two neighbor subunits. This difference—less than 10% of the overall energetic contribution from ligand binding—might be due to the restriction on subunit arrangement imposed by the planar membrane; if this is the case, then the position effect is not expected in hemoglobin, in which each subunit is related equivalently to all the other subunits.

## Significance

The Monod-Wyman-Changeux (MWC) model, proposed more than 50 years ago, is elegantly simple yet powerful in predicting the behavior of allosteric proteins like hemoglobin. Its prediction power for ion channel gating has been beautifully demonstrated in the studies of BK channels. Our present work aims to determine how accurately the MWC model predicts TRPV1 activation induced by vanilloids. Our findings support the notion that the evolutionary drive upon allosteric proteins applies generally to multi-subunit proteins including ion channels.

## Introduction

In the landmark study of neuronal action potential, Hodgkin and Huxley found that the entities controlling transmembrane conductance for sodium and potassium ions—which are now known as voltage-gated sodium (Nav) channels and voltage-gated potassium (Kv) channels—operate with high voltage sensitivities (1). In their empirical equations describing the voltage dependence of sodium and potassium conductance, this high sensitivity is reflected by the exponents assigned to the probability terms. Modern expansions of the H&H ideas incorporating knowledge of Nav and Kv channel structures reveal that high voltage sensitivity is partially rooted in the highly cooperative nature of voltage-dependent activation. In the 1990s, Zagotta, Hoshi, and Aldrich (2) and Schoppa and Sigworth (3) identified a late cooperative transition that would be needed in an otherwise independent subunit gating scheme to satisfactorily describe the course of activation in *Shaker* potassium channels. It is thought that the voltage sensors of *Shaker*, as well as many other voltage-gated ion channels, operate in a mostly independent manner, whereas some of the conformational changes in the channel pore must be concerted (4).

BK potassium channels are activated by not just transmembrane voltage but also intracellular calcium. Their activation also exhibits cooperativity (5). In a comprehensive investigation of BK channel macroscopic currents, single-channel currents, and gating currents, Horrigan, Cui, and Aldrich revealed that the voltage sensor and calcium sensor operate separately, and they both influence the channel pore opening allosterically (6,7,8). The kinetic model that could satisfactorily describe BK activation behaviors thus contains two branches of allosteric coupling. In each branch of the model, the four sensors (for voltage or calcium) contribute an equal amount of energy toward influencing the pore opening. Cooperativity among subunits is rooted in this joint influence of open pore stability.

This type of allosteric coupling has been previously proposed to govern another protein, the oxygen carrier hemoglobin. Hemoglobin is a protein complex made of two α subunits and two β subunits; each of these subunits contains a structurally and functionally similar binding pocket for gas molecules (9). Oxygen binding to these four sites in hemoglobin is highly cooperative (10). Monod, Wyman, and Changeux postulated that the cooperativity comes from a concerted conformational change that affects all gas binding sites equally (11). Hemoglobin serves as a carrier for gas molecules; there is no function equivalent to ion conduction in an ion channel that can be used as a direct indicator of the concerted transition. Studies of hemoglobin therefore focused on the ligand-binding process. It was proposed that there is an equal energetic contribution to the concerted transition by each gas molecule binding step and that these binding steps, per se, are independent (11). Introducing interactions at the binding steps, such as those seen in the sequential model, yields good performance but also adds complexity (12).

Equal and independent contribution to gating by each subunit is assumed for BK channels in the study by Horrigan, Cui, and Aldrich and for many other ion channels studied subsequently. The MWC-type models in general worked well in predicting channel behaviors and provided important guidance for mechanistic investigations in the following decades. Many mechanistic predictions from these studies were nicely confirmed when ion channel structures became available (4). In recent studies of the capsaicin receptor TRPV1 (13,14), we realized that a set of concatemers previously designed by Priel and colleagues (15) would allow us to lock a TRPV1 channel in each of the intermediate ligand-bound states. This and the opportunity to directly access the allosteric transition in an ion channel through current recording present a unique opportunity to directly test the various postulates in the MWC model. This is possible because the concatemers, made of various combinations of wild-type and Y511A mutant protomers, would trap a vanilloid molecule such as resiniferatoxin (RTX) when it binds to a wild-type subunit but allow it to fall off a mutant subunit. Isolating intermediate binding states for equilibrium measurements has been challenging for hemoglobin bound with small gas molecules; it is, to our best knowledge, not done with any concatemeric ion channels. In the present study, we took advantage of this powerful system to address the question of how closely TRPV1 resembles an ideal MWC-type allosteric protein.

## Materials and methods

### Molecular biology

The plasmids used in this study were made in the Priel laboratory (15). Briefly, wild-type rat TRPV1 (Y) cDNAs were joined with the Y511A mutant (A) cDNAs in various combinations: YYYY, YYYA, YYAA, YAYA, AYAY, YAAA, and AAAA. Their functional properties have been carefully tested and described in previous publications; no noticeable change in gating behavior was detected in these studies (13,14,15). Representative single-channel recordings for each concatemer are presented in Figs. S1–S5. Y511 locates near the entrance of the vanilloid binding pocket (16). Its long side chain points downward in the apo state; binding of a vanilloid ligand causes the side chain to flip upwards (17). In this up position, the side chain would slow down a bound ligand from exiting the pocket (14,18). We recently found that the Y512A mutation (with a smaller side chain) in the mouse TRPV1, or the equivalent Y511A mutation in the rat TRPV1, makes binding of RTX and 6′-iodoresiniferatoxin (6′-iRTX) reversible (13,14). Exit of a bound capsaicin, which has the same vanillyl headgroup as RTX but is much small in overall size, is apparently also facilitated by the mutation (19).

### Cell culture

TSA201 cells (HEK293T variant from American Type Culture Collection, Manassas, VA, USA) served as the expression system for patch-clamp assays. These cells were cultivated on 25 mm glass coverslips in 30 mm dishes (from Thermo Scientific, Waltham, MA, USA) until reaching 30%–50% confluence and then transiently transfected. Transfection was carried out using Lipofectamine 2000 (Invitrogen, Carlsbad, CA, USA) 24 h prior to patch-clamp recording, following the manufacturer’s guidelines. For single-channel recordings, a combination of 0.1 *μ*g concatemer plasmid and 0.2 *μ*g EYFP plasmid was utilized per transfection.

### Chemical solutions

For inside-out patch-clamp recordings, we used symmetric bath and pipette solutions containing 140 mM NaCl, 15 mM HEPES, 2 mM EDTA (pH 7.4). Capsaicin (from Sigma-Aldrich, St. Louis, MO, USA) was dissolved in DMSO to prepare a 1 M stock and then further diluted to concentrations ranging from 0.01 to 100 *μ*M using the bath solution. Both RTX (Alomone Labs, Jerusalem, Israel) and 6′-iRTX (Sigma-Aldrich) were dissolved in ethanol to create a 1 mM stock and subsequently diluted to a 200 nM working concentration with the bath solution. Lastly, 2-aminoethoxydiphenyl borate (Sigma-Aldrich) was dissolved in DMSO to produce a 1 M stock, which was then diluted to a 3 mM working solution.

### Electrophysiology

Pipettes for patch-clamp recordings were pulled from borosilicate glass capillaries (Sutter Instrument, Novato, CA, USA) using a P-97 micropipette puller (Sutter Instrument) and fire polished to achieve resistances 8–15 MΩ for single-channel recordings. We employed an EPC 10 USB patch-clamp amplifier (Warner Instruments, Hamden, CT, USA) operated by the PatchMaster software. Sampling and filtering frequencies were set at 10 kHz and 2.25 kHz, respectively. Patch-clamp configurations were primarily inside out unless specifically stated otherwise. The holding potential began at 0 mV and proceeded at steps of +80 and −80 mV. Step durations were adjusted as required. A gravity-driven perfusion system, controlled by the Rapid Solution Changer (RSC-200, BioLogic, Seyssinet-Pariset, France), facilitated solution perfusion and changes.

### Data analysis

Patch-clamp data, exported from PatchMaster in the Igor format, were analyzed using Igor Pro 8 (WaveMetrics, Portland, OR, USA). Statistical analyses were conducted in GraphPad Prism 8. Two-way ANOVA tests were done to detect difference between YYAA (ligand binding in neighboring subunits) versus YAYA or AYAY (ligand binding in kitty-corner subunits). Detailed results for the two-way ANOWA test are included in Tables S1–S8. Data are presented as mean ± SEM.

Single-channel open probabilities were determined by creating all-point histograms from single-channel current traces. For recordings from a single channel, the histogram contained two peaks, whose area represented the total time spent in the closed state, *t*_*c*_, and the open state, *t*_*o*_, respectively. The open probability was calculated as $Po=\frac{t_{o}}{t_{o}+t_{c}}$. For patches containing two channels, the histogram contained two open-state peaks corresponding to one and two channels in the open state. The areas under them, *t*_*1*_ and *t*_*2*_, respectively, were used to determine the open probability using the equation $Po=\frac{t_{1}+2\times t_{2}}{2\times T}$. Here, T represents the total area of the histogram. Recordings with more than two channels were discarded.

### Model fitting

As an initial analysis, capsaicin responses of concatemeric channels were fitted to a Hill equation

$$Po=P_{o\_base}+\frac{\left(P_{o\_\max}-P_{o\_base}\right)}{1+{\left(\frac{\text{EC}50}{\left[Cap\right]}\right)}^{n}}\text{,}$$

where EC50 is the concentration for 50% activity, [*Cap*] is the capsaicin concentration, and *n* is the Hill slope factor. Capsaicin responses of concatemeric channels pretreated with RTX or 6′-iRTX were further fitted to models with specific modifications of the MWC model (shown in Fig. 2). The goal of these analyses was to obtain an upper limit estimate for the free energy representing the deviation from an ideal MWC system. For these analyses, a global fitting routine was applied simultaneously to data from all three concatemers pretreated with either RTX or 6′-iRTX. The global fitting package in Igor Pro 8 was used to fit these single-channel *Po* data.

To obtain free energy estimates, we considered two extreme situations. For the first situation, we assumed that the observed position effect was solely due to differences in capsaicin binding affinity. This could be reflected by assigning a cooperativity coefficient to the first ligand-binding step in the MWC-type model for a two-site system while keeping the other parameters unchanged. Shown in Fig. 3, *A* and *B*, are equilibrium constants for all the transitions, where $L_{2}$ is the equilibrium constant for channels preloaded with two RTX or 6′-iRTX molecules, [c] is the concentration of capsaicin in the perfusion solution, K is the binding affinity parameter, *a* and *a*′ are the cooperativity coefficients for binding, and f is the gating parameter (fold change to *L*_*2*_ as a result of each capsaicin binding step). Solving this model under the equilibrium condition yields the following equation:

$$Po=\frac{L_{2}+2afK\left[c\right]L_{2}+af^{2}K^{2}{\left[c\right]}^{2}L_{2}}{{1+2aK\left[c\right]+aK^{2}{\left[c\right]}^{2}+L}_{2}+2afK\left[c\right]L_{2}+af^{2}K^{2}{\left[c\right]}^{2}L_{2}}\text{.}$$

Global fitting results using this model are shown in Fig. 3 *C*, where the same set of values for *f* and *K* were used and the values for *L*_*2*_, *a* (applied to the YYAA data set), and *a*′ (applied to the YAYA and AYAY data sets) were allowed to vary between different groups.

For the second extreme situation, we assumed that the observed position effect was solely due to a difference in the activation transition. This could be reflected by assigning a cooperativity coefficient to the first vertical gating transition (which would be carried on to the second gating transition and the first binding transition in the open state, to maintain microscopic reversibility) while keeping the other parameters unchanged. Shown in Fig. 4, *A* and *B*, are equilibrium constants for all the transitions, where individual parameters are defined in the same way as described above, and *b* and *b*′ are the cooperativity coefficients for gating (ratio factor between the two gating equilibrium constants following the first and second capsaicin binding steps). Solving this model under the equilibrium condition yields the following equation:

$$Po=\frac{L_{2}+2bfK\left[c\right]L_{2}+bf^{2}K^{2}{\left[c\right]}^{2}L_{2}}{{1+2K\left[c\right]+K^{2}{\left[c\right]}^{2}+L}_{2}+2bfK\left[c\right]L_{2}+bf^{2}K^{2}{\left[c\right]}^{2}L_{2}}\text{.}$$

Global fitting results using this model are shown in Fig. 4 *C*, where the same set of values for *f* and *K* were used and the values of *L*_*2*_, *b* (applied to the YYAA data set), and *b*′ (applied to the YAYA and AYAY data sets) were allowed to vary between different groups.

For comparison, a classic MWC model without a position effect was also used to fit the group data (*gray curves* in Figs. 3 *C* and 4 *C*):

$$Po=\frac{L_{2}+2fK\left[c\right]L_{2}+f^{2}K^{2}{\left[c\right]}^{2}L_{2}}{{1+2K\left[c\right]+K^{2}{\left[c\right]}^{2}+L}_{2}+2fK\left[c\right]L_{2}+f^{2}K^{2}{\left[c\right]}^{2}L_{2}}\text{.}$$

To directly determine the position effect on free energy associated with the gating transitions, we measured the *Po* values when a different number of RTX molecules were bound to the channel. This was achieved by treating each of the YYYY, YYYA, YYAA, YAYA, AYAY, YAAA, and AAAA channels for an extended time, followed by a thorough wash to remove the reversible binding of RTX to the A subunits, as previously described (13). The following equations were used:

For AAAA (with no RTX bound): $Po=\frac{L_{0}}{1+L_{0}}$.

For YAAA (with 1 RTX bound): $Po=\frac{{f_{1}L}_{0}}{1+f_{1}L_{0}}$.

For YAYA and AYAY (with 2 RTX bound at diagonal sites): $Po=\frac{{f_{2}L}_{0}}{1+f_{2}L_{0}}$.

For YYAA (with 2 RTX bound at adjacent sites): $Po=\frac{{{f_{1}}^{2}L}_{0}}{1+{f_{1}}^{2}L_{0}}$.

For YYYA (with 3 RTX bound): $Po=\frac{{f_{1}f_{2}L}_{0}}{1+f_{1}f_{2}L_{0}}$.

For YYYY (with 4 RTX bound): $Po=\frac{{{f_{2}}^{2}L}_{0}}{1+{f_{2}}^{2}L_{0}}$.

In these equations, $L_{0}$ is the equilibrium constant for channels in the apo state, $f_{1}$ is the gating parameter when only one RTX is bound in the diagonal pair of the binding sites, and $f_{2}$ is the gating parameter when two RTX are bound in the diagonal pair of the binding sites. The results of global fitting of these equations simultaneously to the corresponding data sets are shown in Fig. 5 *B*.

The classic MWC model would predict for this situation the following equation: $Po=\frac{{f^{n}L}_{0}}{1+f^{n}L_{0}}$, where $L_{0}$ is the equilibrium constant for channels in the apo state, f is the gating parameter for each RTX bound to the channel, and n is the number of bound RTX. A prediction of this model is included in Fig. 5 *B* for comparison.

## Results

TRPV1 is an allosteric protein. In the absence of ligands such as capsaicin or RTX, TRPV1 can spontaneously transition from a closed state to an open state at a very low probability (with an equilibrium constant less than 0.01) (13,20). As an increasing number of agonist molecules bind to TRPV1, the equilibrium progressively shifts toward the open state. Our previous study showed that, during RTX activation of TRPV1, sequential RTX bindings shift the equilibrium nearly exponentially, suggesting that energy contributions from these RTX binding steps are approximately equal (13) as the classic MWC model postulates (11). Binding of all four subunits with RTX molecules contributes a total energy of 6.8–7.4 kcal/mol toward activation (13).

**Figure 1:**
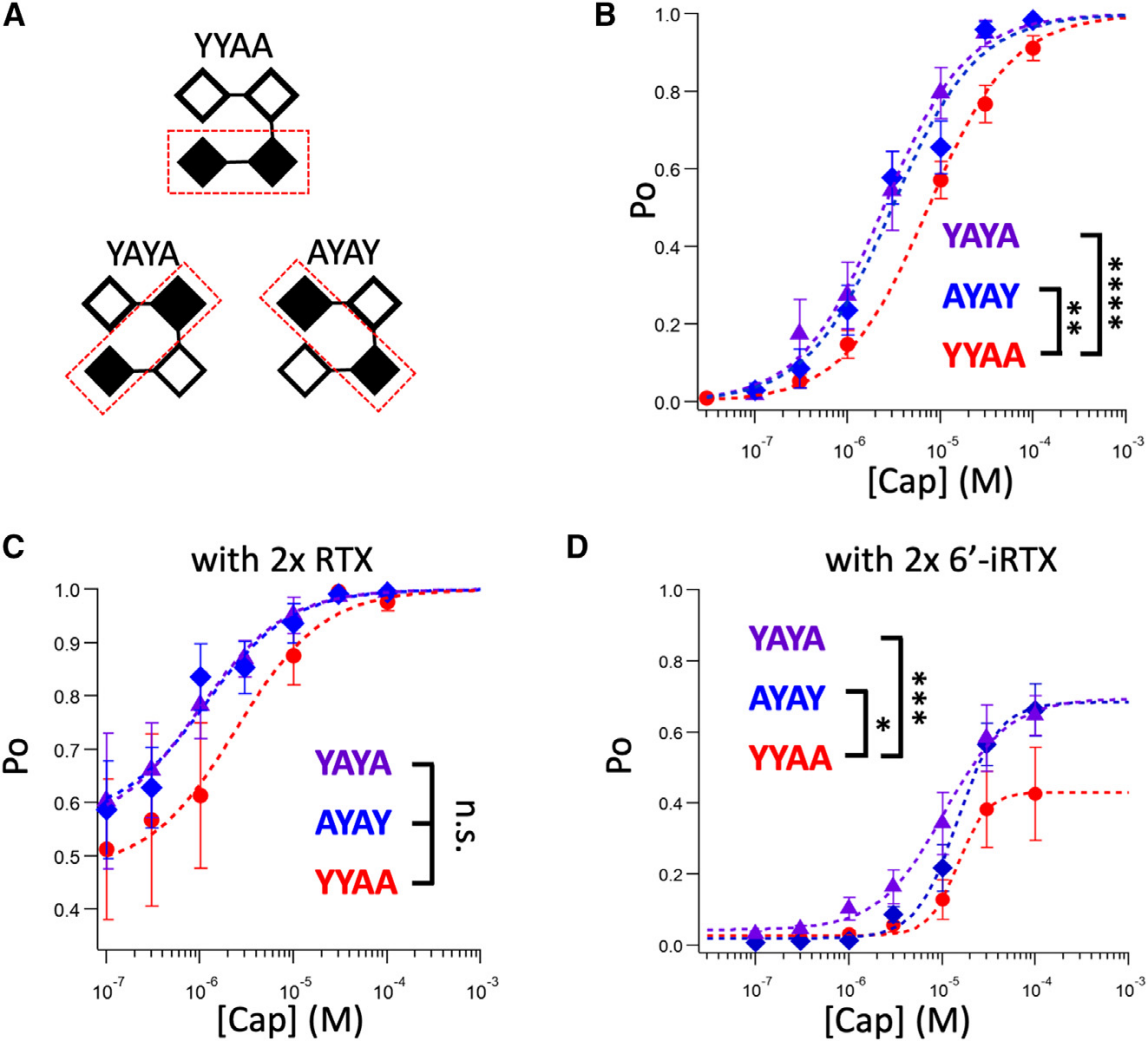
(*A*) Expected subunit positioning for YYAA, YAYA, and AYAY concatemers; an open square represents a wild-type subunit, and a filled square represents a Y511A mutant subunit. (*B*) Capsaicin concentration-dependent single-channel open probability curves for YYAA, YAYA, and AYAY fitted to a Hill equation using the following EC50 and Hill slope parameters: YYAA (*red*), 7.31 *μ*M, 0.87, *n* = 12; AYAY (*blue*), 2.99 *μ*M, 0.95, *n* = 4; YAYA (*black*), 2.42 *μ*M, 0.97, *n* = 5. (*C*) Capsaicin concentration-dependent single-channel open probability curves for YYAA, YAYA, and AYAY with two preloaded RTX molecules in the wild-type protomers fitted to a Hill equation: YYAA, 2.42 *μ*M, 0.93, *n* = 5; AYAY, 0.97 *μ*M, 0.94, *n* = 5; YAYA, 1.13 *μ*M, 0.95, *n* = 5. (*D*) Capsaicin concentration-dependent single-channel open probability curves for YYAA, YAYA, and AYAY with two preloaded 6′-iRTX molecules in the wild-type protomers fitted to a Hill equation: YYAA, 14.77 *μ*M, 2.72, *n* = 4; AYAY, 14.61 *μ*M, 1.95, *n* = 6; YAYA, 10.25 *μ*M, 1.24, *n* = 8. Error bars represent SEM. Two-way ANOVA with Sidak’s multiple comparisons test; n.s., no significance; ^∗^*p* < 0.05, ^∗∗^*p* < 0.01, ^∗∗∗^*p* < 0.001, and ^∗∗∗∗^*p* < 0.0001.

However, when we closely examined the capsaicin response curves for YAYA, AYAY, and YYAA concatemers—all producing channels with two wild-type subunits and two mutant subunits but with different subunit arrangements (Fig. 1 *A*)—a minor but apparent difference could be discerned. Both YAYA and AYAY (with two mutant subunits located at kitty-corners in the assembled channels) appeared to be slightly more sensitive to capsaicin than YYAA (with mutant subunits at neighbor positions) (Fig. 1 *B*). The small differences in the EC50 value, obtained from fitting with a Hill function, would translate into free energy differences of about 0.66 kcal/mol between YYAA and YAYA and 0.53 kcal/mol between YYAA and AYAY. The group data for YAYA and AYAY are significantly deviated from the group data for YYAA (*p* < 0.0001 and *p* = 0.005, respectively) (Table S1). Nonetheless, at most specific capsaicin concentrations, it was hard to confidently establish a statistically significant difference in *Po*. (One of the five recordings from AYAY yielded an abnormal right-shifted capsaicin dependence curve, which was excluded from Fig. 1 *B*. While the cause of this abnormality is unknown, there are previous reports that a concatemeric construct may sometimes fail to constrain the subunit composition. Including this outlier recording would make the difference between AYAY and YYAA even smaller [see Fig. S4].)

Given the uncertainty, we assessed the potential subunit positioning effect with a different approach. Taking advantage of the reversible binding of RTX to the A subunits and irreversible binding to the Y subunits, we first fully loaded each channel type with RTX, followed by a thorough washing. As we reported previously (13,14), this procedure yielded channels containing two Y subunits bound with RTX and two A subunits available for subsequent binding by capsaicin. We measured the channel open probability at increasing concentrations of capsaicin. As shown in Fig. 1 *C*, a slightly higher capsaicin sensitivity in YAYA and AYAY concatemeric channels was again observed, which is reflected in the uplifted concentration-dependent curves, though this apparent difference was not statistically significant (*p* = 0.16 and 0.15) (Table S2). Similar results were obtained using 6′-iRTX, an RTX derivative that is a much weaker TRPV1 agonist: a small yet statistically significant (*p* = 0.0009 and 0.015) upward shift in the capsaicin concentration-dependent curves for AYAY and YAYA could be seen (Fig. 1 *D*).

**Figure 2:**
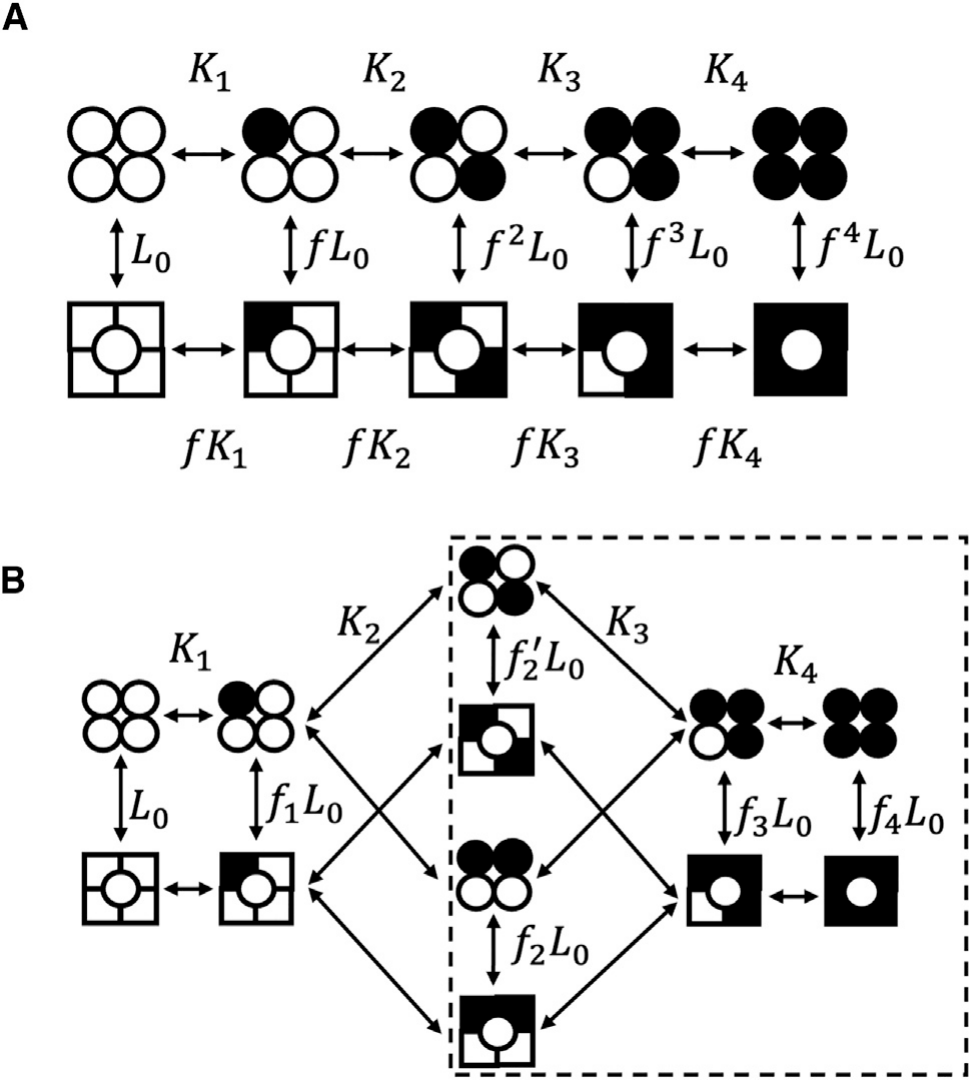
(*A*) Illustration of a classic MWC model for a tetrameric ligand-gated ion channel with four identical ligand-binding sites. *L*_0_ represents the equilibrium constant for the apo state, and *f* represents the cooperative factor, which reflects the energy contribution by each ligand binding; *K*_1_–*K*_4_ represent the ligand association constants. (*B*) Illustration of a modified model with position effects. The dashed box shows the subsystem examined in Figs. 3 and 4.

Our results indicate that, when two ligands bind to a single TRPV1 channel, the diagonal (kitty-corner) binding positions produce a higher open probability than the adjacent binding positions. This behavior is not aligned with the prediction of the classic MWC model, which assumes equality in subunit contributions (Fig. 2 *A*). To experimentally assess the magnitude of this position effect, we used a modified model incorporating distinct states for the two-ligand bound configurations, as shown in Fig. 2 *B*. In this expanded model, the increased cooperativity for the diagonal binding positions could arise either from the binding process (characterized by the *K* parameter) or the gating process (characterized by the *f* parameter). We evaluated these possibilities separately in the following two sets of experiments using single-channel *Po* data in response to capsaicin after the two Y subunits in each channel were preloaded with RTX or 6′-iRTX molecules (the subsystem is shown by the *dashed box* in Fig. 2 *B*).

**Figure 3:**
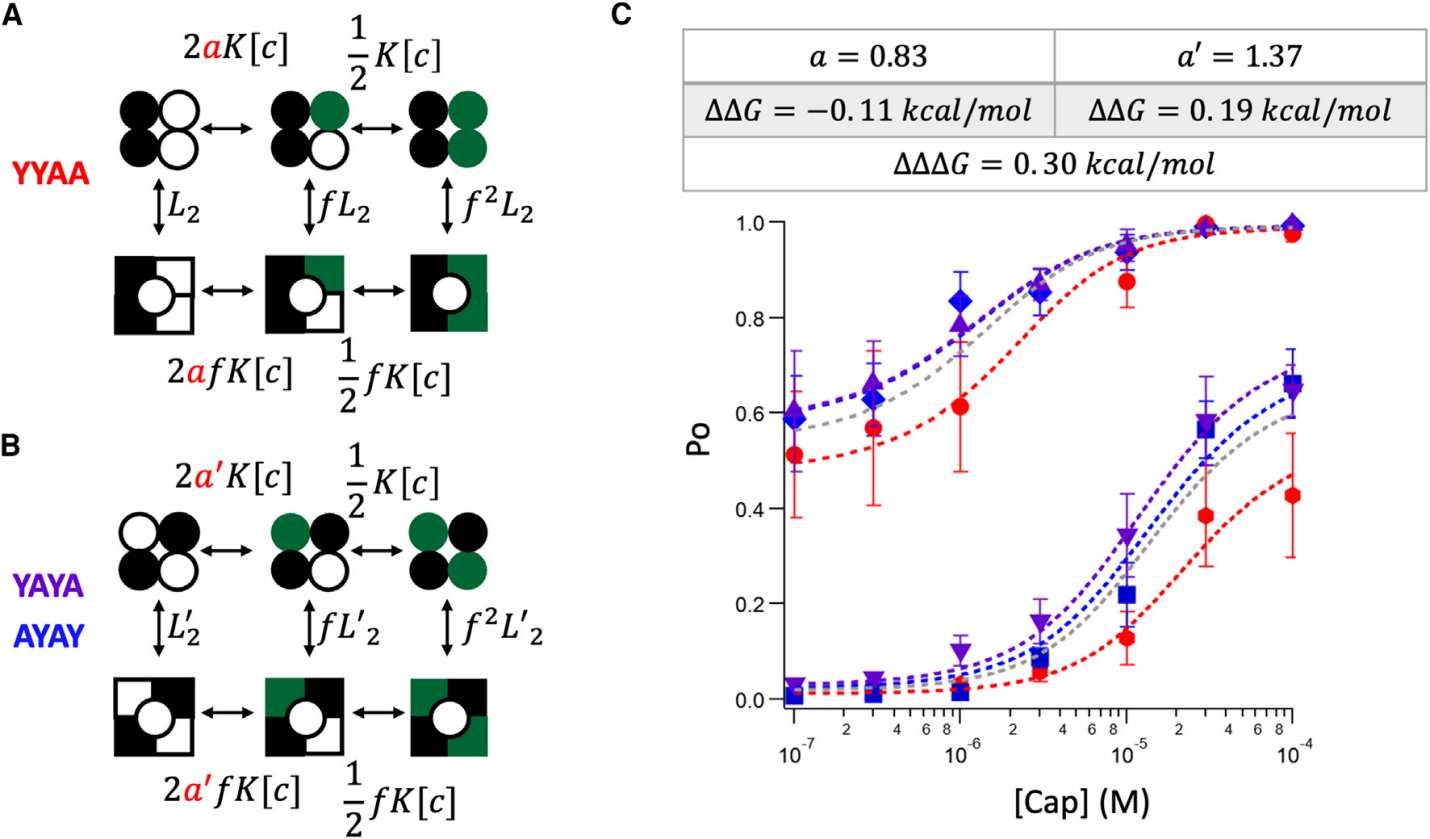
(*A* and *B*) Illustration of the models used for fitting the capsaicin-dependent *Po* data with two preloaded RTX or 6′-iRTX to test the contribution of binding to the position effect. *L*_2_ and *L*_2_′ represent the equilibrium constants for the state with two preloaded RTX or 6′-iRTX (*L*_2_ for YYAA, *L*_2_′ for YAYA and AYAY) before application of capsaicin; *a* and *a*′ are coefficient factors for the position effect (*a* for YYAA, *a*′ for YAYA and AYAY); when there is no position effect, *a* and *a*′ should be 1 for independent bindings; *f* represents the cooperative factor, which reflects the energy contribution by each ligand binding; *K* represents the ligand binding affinity constant for the Y511A mutant subunit, and [*c*] is the capsaicin concentration. (*C*) Global fitting results using the models shown in (*A*) and (*B*) with the following parameters: *a* = 0.83; *a*′ = 1.37; *K* = 4.8 × 10^4^ M^−1^, *f* = 10.3; YYAA: *L* (with 6′-iRTX) = 0.01, *L* (with RTX) = 0.92; YAYA: *L* (with 6′-iRTX) = 0.03, *L* (with RTX) = 1.38; AYAY: *L* (with 6′-iRTX) = 0.02, *L* (with RTX) = 1.35. Deviations of the *a* and *a*′ values from 1 (for independent ligand binding) represent ΔΔ*G* values of −0.11 (for YYAA) and 0.19 kcal/mol (for YAYA and AYAY). For model fitting with the classic MWC model (*gray line*): *K* = 5.8 × 10^4^ M^−1^, *f* = 10.4, *L* (with 6′-iRTX) = 0.02, *L* (with RTX) = 1.16. Error bars represent SEM.

To assess a potential subunit position effect on binding affinity, we first assumed the gating effect (*f*) to be independent of the position effect (Fig. 3, *A* and *B*); that is, variation in gating cooperativity was assumed to originate solely from binding, hence potentially exaggerating the binding effect. We assigned the equilibrium constant for the last binding step the same *K* parameter, since this step is identical for all concatemers, as can be seen in Fig. 3, *A* and *B*. However, transitioning from a two-ligand-bound state to a three-ligand-bound state might show sensitivity to the initial positions of liganded subunits, leading us to introduce a coefficient factor *a* or *a*′ to the binding parameter *K*. In the absence of a position effect, both *a* and *a*′ would be 1, as expected from independent binding. However, if a position effect existed, then *a* and *a*′ would differ. As described earlier, we first treated each concatemeric channel with RTX or 6′-iRTX followed by a thorough wash; this yielded channels in which the wild-type Y subunits were occupied (*black filled symbols* in Fig. 3, *A* and *B*) and the mutant subunits available for binding by capsaicin. Global fitting of the capsaicin response data sets from all concatemers pretreated with either RTX or 6′-iRTX (Figs. 3 *C* and S5, *left*) yielded values of *a* = 0.83 and *a*′ = 1.37, suggesting the presence of a position effect, albeit one that is rather small. These factors represent free energy differences from independence (*a* and *a*′ = 1) by −0.11 and 0.19 kcal/mol, respectively. The kitty-corner bound subunit arrangement deviates more noticeably. The free energy difference between the two subunit arrangements would be ΔΔΔ*G* = 0.30 kcal/mol. Considering that our method likely overestimated binding cooperativity, the actual difference would be smaller.

**Figure 4:**
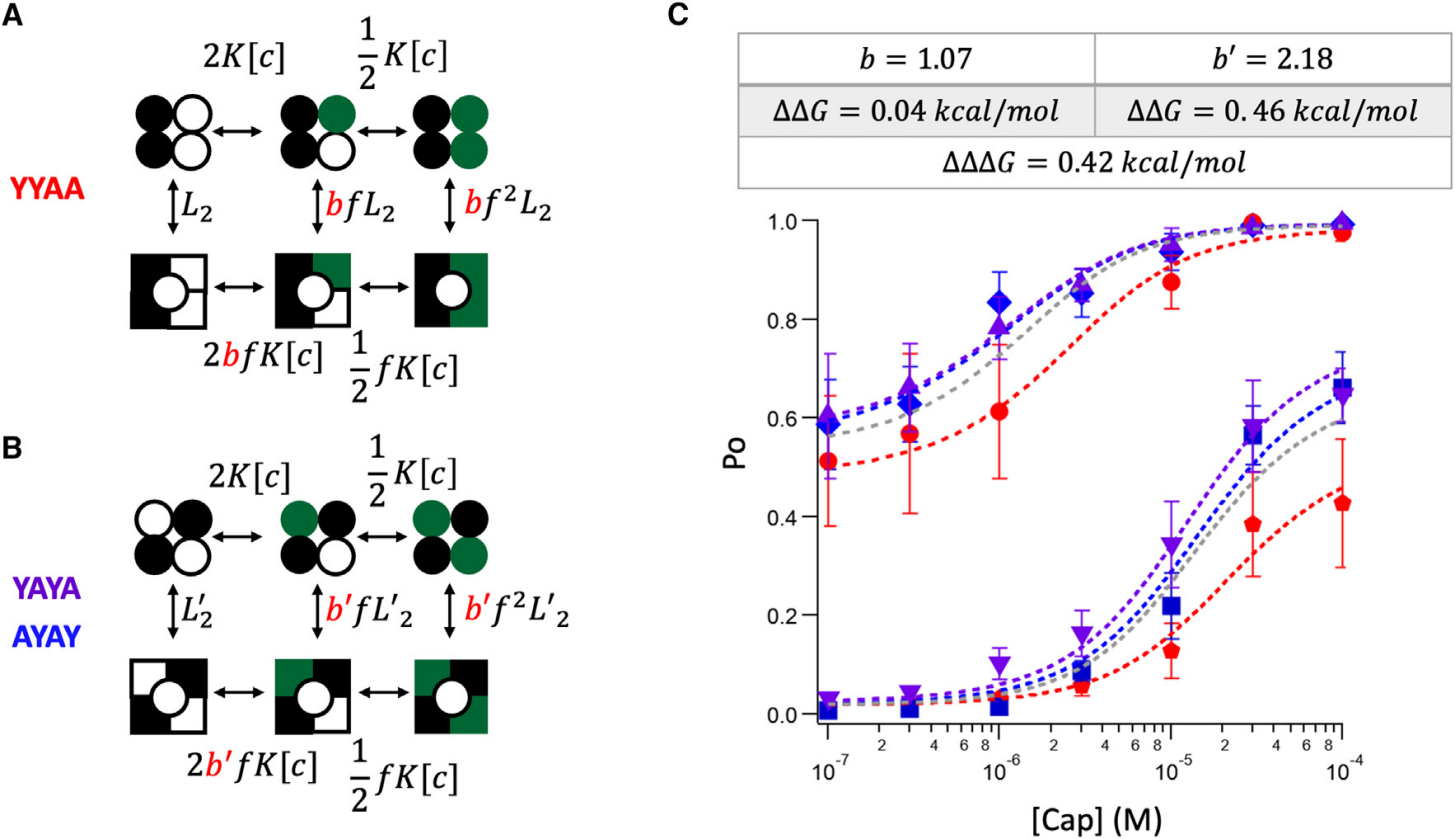
(*A* and *B*) Illustration of the models for testing the contribution of gating to the position effect. *L*_2_ and *L*_2_′ represent the equilibrium constants for the state with two preloaded RTX or 6′-iRTX (*L*_2_ for YYAA, *L*_2_′ for YAYA and AYAY) before application of capsaicin; *b* and *b*′ are coefficient factors for the position effect (*b* for YYAA, *b*′ for YAYA and AYAY); when there is no position effect, *b* and *b*′ should be 1; *f* represents the cooperative factor, which reflects the energy contribution by each ligand binding; *K* represents the ligand binding affinity constant for the Y511A mutant subunit, and [*c*] is the capsaicin concentration. (*C*) Global fitting results using the models from (*A*) and (*B*) with the following parameters: *b* = 1.07; *b*′ = 2.18; *K* = 4.6 × 10^4^ M^−1^, *f* = 7.8; YYAA: *L* (with 6′-iRTX) = 0.02, *L* (with RTX) = 0.94; YAYA: *L* (with 6′-iRTX) = 0.02, *L* (with RTX) = 1.32; AYAY: *L* (with 6′-iRTX) = 0.02, *L* (with RTX) = 1.28. Deviations of the *b* and *b*′ values from 1 (no position effect) represent ΔΔ*G* values of 0.04 (for YYAA) and 0.46 kcal/mol (for YAYA and AYAY). For model fitting with classic MWC model (*gray line*): *K* = 5.8 × 10^4^ M^−1^, *f* = 10.4, L (with 6′-iRTX) = 0.02, *L* (with RTX) = 1.16. Error bars represent SEM.

To gauge the gating effect, we used a similar approach, assuming the binding steps were independent (Fig. 4, *A* and *B*). This would attribute any binding cooperativity to gating, potentially exaggerating the gating effect. For the reason discussed earlier, we assigned the same *f* factor when the channel transitioned from a three-ligand-bound state to a four-ligand-bound state. We introduced a coefficient factor *b* or *b*′ to the gating parameter *f* for transitions from a two-ligand-bound state to a three-ligand-bound state. Without a position effect, *b* and *b*′ would be 1; otherwise, they would differ. Global fitting of the same data sets yielded values of *b* = 1.07 and *b*′ = 2.18, corresponding to free energy deviations from equal subunit contributions (*b* and *b*′ = 1) by 0.04 and 0.46 kcal/mol (Figs. 4 *C* and S5, *right*). Again, the kitty-corner bound subunit arrangement deviates more noticeably. The free energy difference between the two subunit arrangements would be ΔΔΔ*G* = 0.42 kcal/mol.

**Figure 5:**
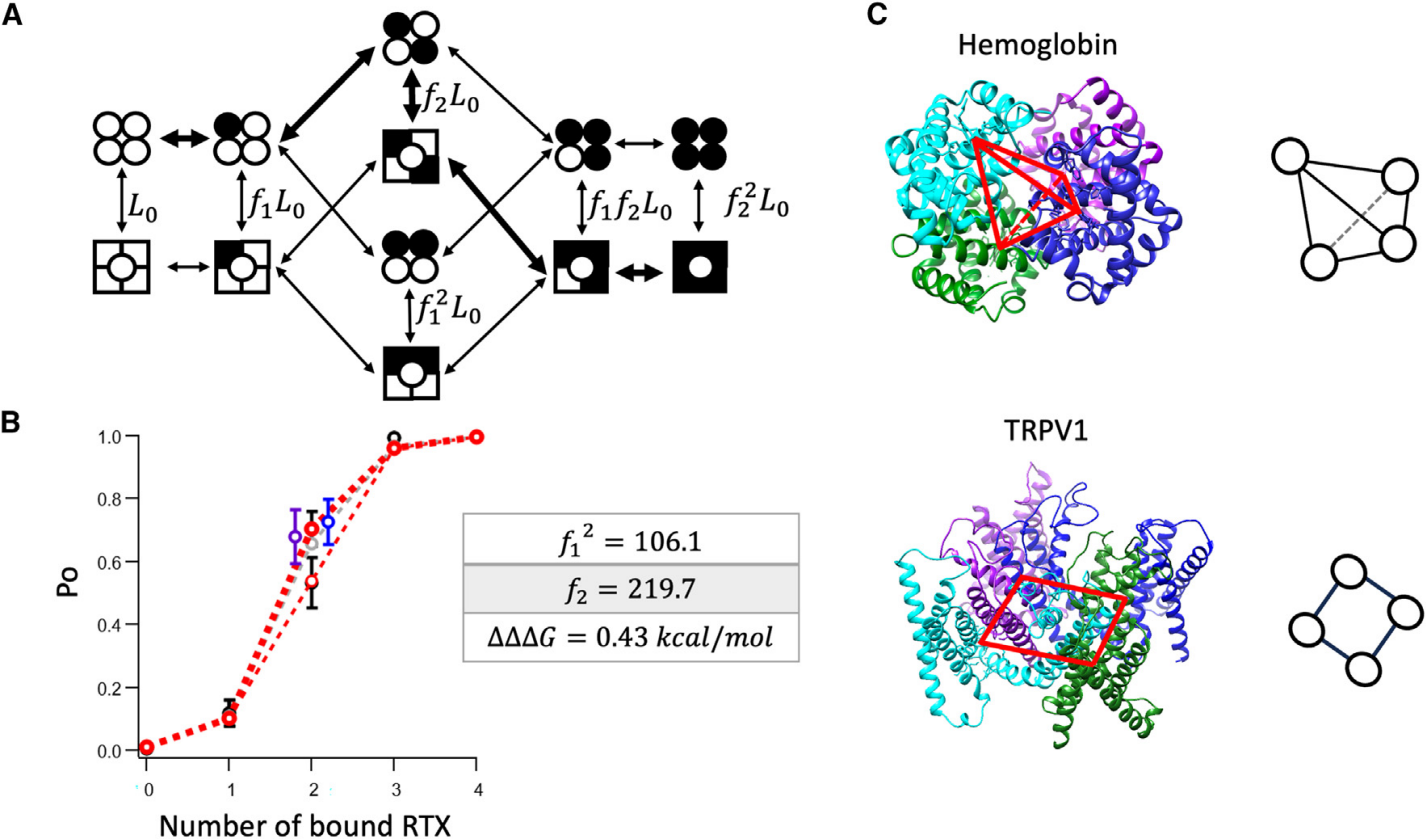
(*A*) Illustration of a modified MWC model with position effects. *L*_0_ represents the equilibrium constant for the apo state, *f*_1_ represents the cooperative factor reflecting the energy contribution by one ligand binding to the pair of diagonal binding sites; *f*_2_ represents the cooperative factor reflecting the energy contribution by two ligands binding to the pair of diagonal binding sites; when there is no position effect, *f*_2_ should equal to *f*_1_^2^, making the model equivalent to the classic MWC model. Thicker arrows indicate pathways with higher probabilities. (*B*) Global fitting results for the *Po* data from AAAA, YAAA, YYAA, YAYA, AYAY, YYYA, and YYYY concatemers, with the Y subunits loaded with RTX: *L*_0_ = 0.01, *f*_1_ = 10.3, *f*_2_ = 219.7, which produce ΔΔΔ*G* = 0.43 kcal/mol between neighbor and kitty-corner positions, *n* = 4–10. Black circles represent the data points; red circles represent predictions of our modified MWC model; gray dashed lines represent a classic MWC model with *L*_0_ = 0.01, *f* = 15.7. The purple circle represents the YAYA data point, and the blue circle represents the AYAY data point. Error bars represent SEM. (*C*) Relationships between ligand-binding pockets in hemoglobin (PDB: 2DHB, *top*) and tetrameric ligand-gated ion channels such as TRPV1 (transmembrane part of TRPV1, modified from PDB: 3J5R, *bottom*). Positions of ligand-binding sites are highlighted by red lines between them and illustrated by a diagram on the right.

Since both fitting methods could potentially skew the estimated position effect, we explored a direct approach to estimate the free energy difference associated with the position effect on gating. We have previously shown that, by fully loading each concatemer with RTX followed by thorough washing, we could obtain channels with one to four RTX-bound subunits (13). *Po* measurements from these channels reflect only the gating equilibrium of each vertical transition seen in Fig. 5 *A* without confounding effects from binding. The difference between two-ligand-bound channels could be represented by introducing just one additional degree of freedom to the classic MWC model. We assigned *f*_1_ as the gating coefficient for a single independent binding site and hypothesized that each pair of diagonal binding sites would exhibit stronger cooperativity (*f*_2_) than two independent sites (*f*_1_^2^). Using this model to fit the RTX-bound TRPV1 single-channel *Po* data (Fig. 5 *B*), we found *f*_2_ to be 107% larger than *f*_1_^2^, equivalent to a free energy difference ΔΔΔ*G* = 0.43 kcal/mol. This result aligns nicely with our indirect estimates discussed earlier, suggesting that, while binding might contribute to the position effect (probably much less than 0.30 kcal/mol), gating plays a larger role, contributing approximately 0.4 kcal/mol. The position effect would favor traversing the diagonally liganded conformation over the adjacently liganded conformation in a roughly 2:1 ratio. Nonetheless, compared to the 6.8–7.4 kcal/mol total energetic contribution from ligand binding to activation, the position effect is small (less than 10%).

## Discussion

The present paper is the third in a series of studies of TRPV1 activation by vanilloid molecules (13,14). Our collective results from these studies suggest that, as an allosteric protein, TRPV1 can exist in various conformations (closed versus open and with various numbers of ligands) at any ligand concentration. A recent structural study has captured many of these conformations, including those with two bound ligands at either neighbor or kitty-corner positions (21). It remains unclear what might contribute to the small difference in stability between these two configurations. The cryoelectron microscopy (cryo-EM) structures reveal that a vanilloid molecule bound in its binding pocket does not belong strictly to one subunit: due to the domain-swapped arrangement, the vanilloid binding pocket is formed by the S3 and S4 segments and the S4-S5 linker from one subunit together with the S5 and S6 segments from a neighbor subunit (16). Whereas capsaicin forms hydrogen bonds with the S4 segment and the S4-S5 linker of the same subunit, extensive hydrophobic interactions with the S5 and S6 segments are predicted from both the cryo-EM structures and computational modeling (22). Polar interactions between capsaicin or other ligands and the S6 segment are also predicted (23,24,25). However, results from our recent study and the present study showed that capsaicin binding to the four TRPV1 subunits are independent events (14), suggesting that the position effect might have a different origin (see below).

One intriguing question that arises from the present study is whether hemoglobin exhibits position differences in ligand-induced allosteric transition like what we have observed in TRPV1. To our best knowledge, no direct experimental evidence in support of such a possibility has been reported. The binding pockets for gas molecules in hemoglobin are formed within each of the four isolated gas molecule binding domains (9). These binding pockets are positioned at the vertex corners of a tetrahedron such that each pocket is related to all the other pockets nearly equally (Fig. 5 *C*, *top*). TRPV1 and other ion channels existing in a planar membrane do not share this symmetry. A vanilloid binding pocket in TRPV1 is related differently to its neighbor pockets and the kitty-corner pocket (Fig. 5 *C*, *bottom*). When two neighbor binding pockets are occupied by ligands, the channel complex lacks the rotational symmetry exhibited when the kitty-corner binding pockets are occupied. Our observations showed that diagonally liganded channels have a higher probability residing in the open state (corresponding to the relaxed state in the MWC model). The situation would not be found in hemoglobin.

Despite the geometric restriction imposed by the planar membrane, a centrally located ion permeation pore surrounded by three to five structurally similar subunits or domains evolves as the overwhelmingly dominant architecture for biological ion channels (4). An obvious benefit of this architecture is the possibility for cooperative control of ion permeation by the multiple subunits or domains, which bestows a much greater sensitivity compared to a monomeric functional unit. Simply combining functional units into a complex without cooperativity does not benefit from this advantageous feature. Heteromeric subunit combination offers further opportunities to yield a wider variety of channel types from limited genetic resource (4) while retaining the advantageous feature of cooperativity. The majority of ligand-gated ion channels have each subunit participating in ligand binding, a situation that would maximize the benefit of cooperativity. Intriguingly, many pentameric channels do not possess five ligand-binding sites. Acetylcholine receptors, for example, often have only two ligand-binding sites (26). In these cases, the ligand-bound sites are always separated across the channel protein complex, as if this arrangement would allow acetylcholine binding to exert the maximal energetic effect on channel activation.

## Author contributions

Conceptualization, S.L. and J.Z.; patch clamp, S.L.; data analysis, S.L. and J.Z.; funding acquisition, J.Z.; supervision, J.Z.; writing – original draft, S.L. and J.Z.; writing – review & editing, S.L. and J.Z.

## Supporting material

Document S1. Figures S1–S5 and Tables S1–S8

Document S2. Article plus supporting material
