# Supplementary material for: How much does TRPV1 deviate from an ideal MWC-type protein?
Source: Biophys J. 2024 Apr 6;123(14):2136–44. doi: 10.1016/j.bpj.2024.04.005 (PMC11309965; doi:10.1016/j.bpj.2024.04.005)
Supplement: Document S1. Figures S1–S5 and Tables S1–S8 [file mmc1.pdf]

Biophysical Journal, Volume *123*

Supplemental information

How much does TRPV1 deviate from an ideal MWC-type protein?

Shisheng Li and Jie Zheng

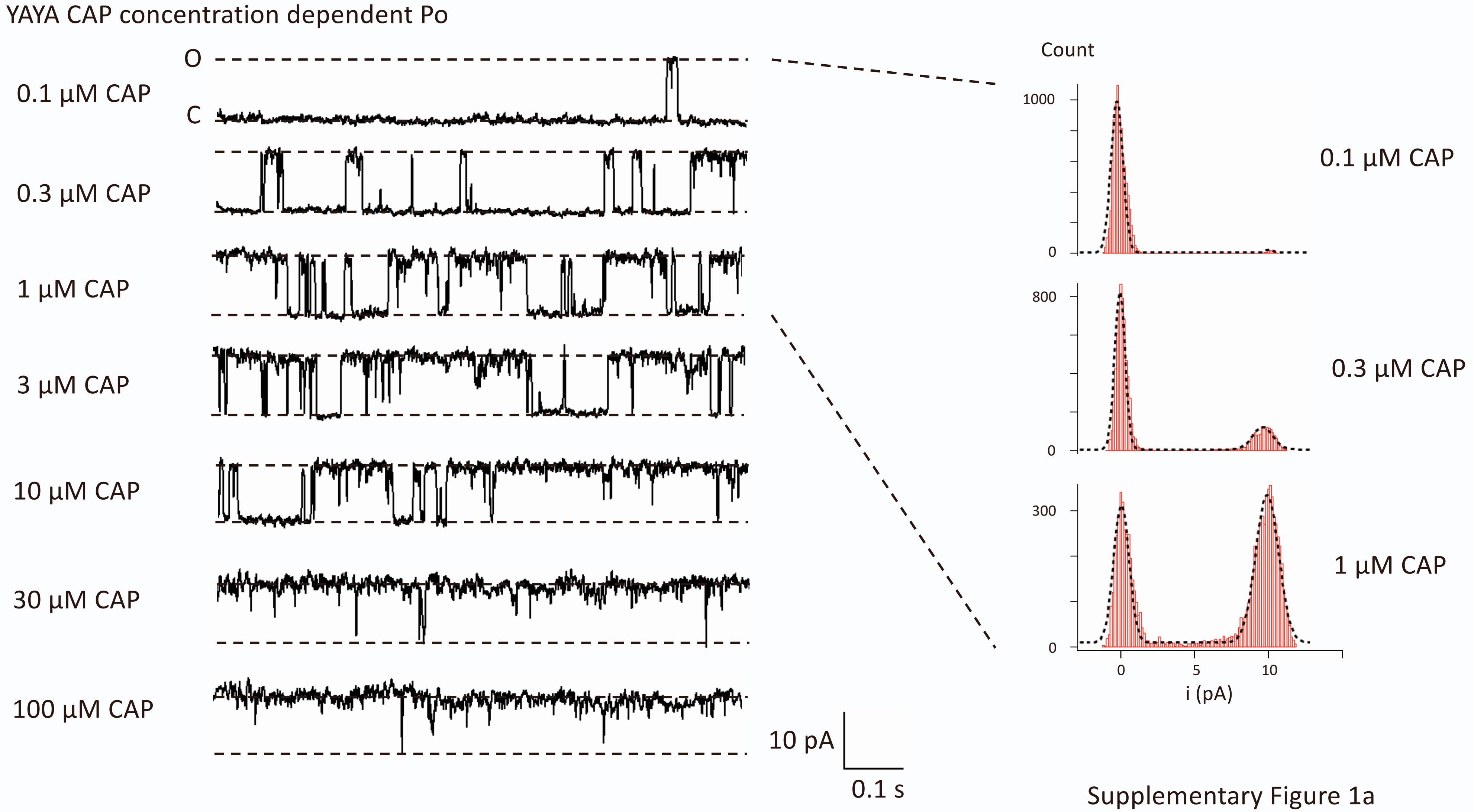

Supplementary Figure 1a

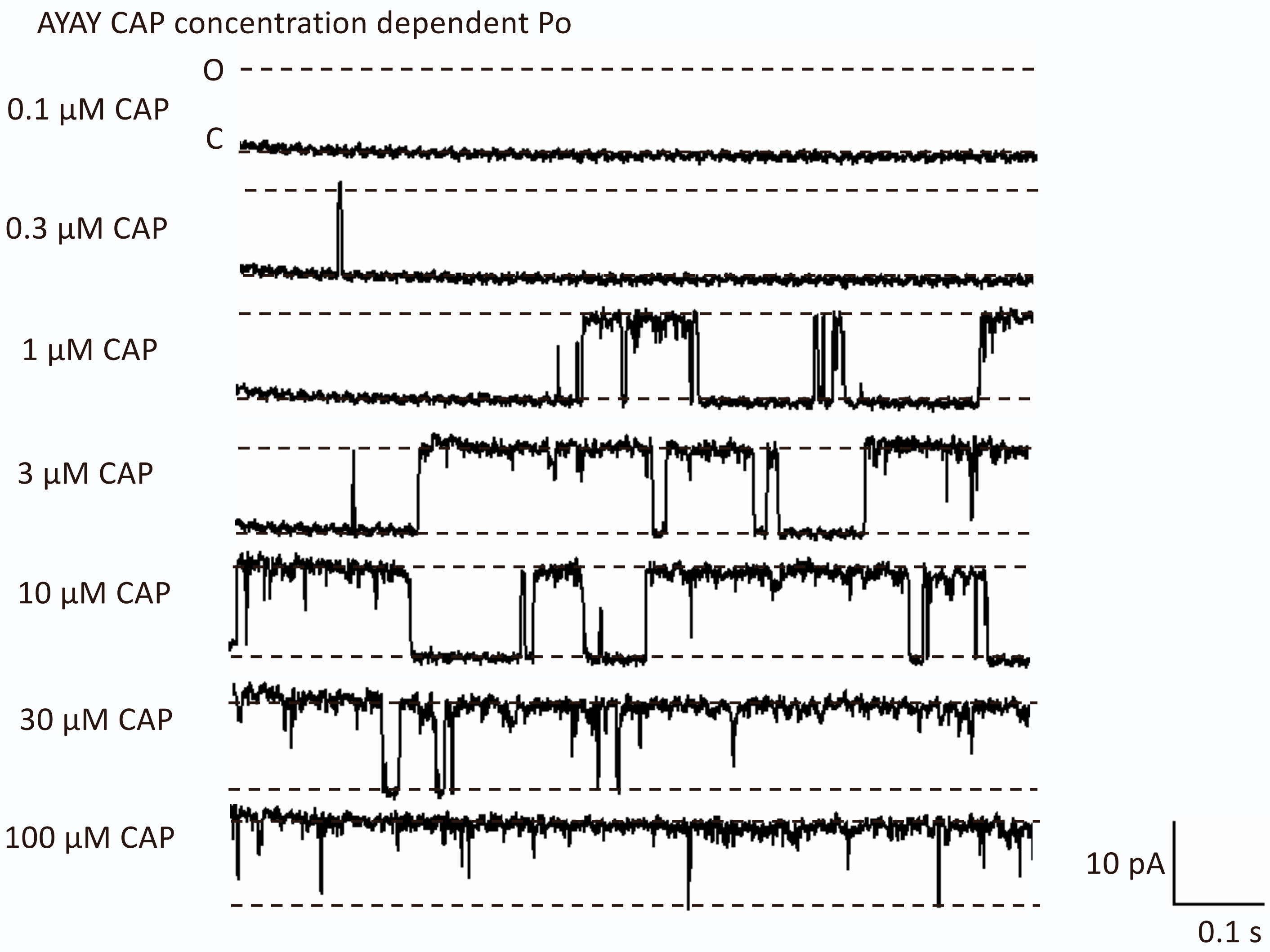

Supplementary Figure 1b

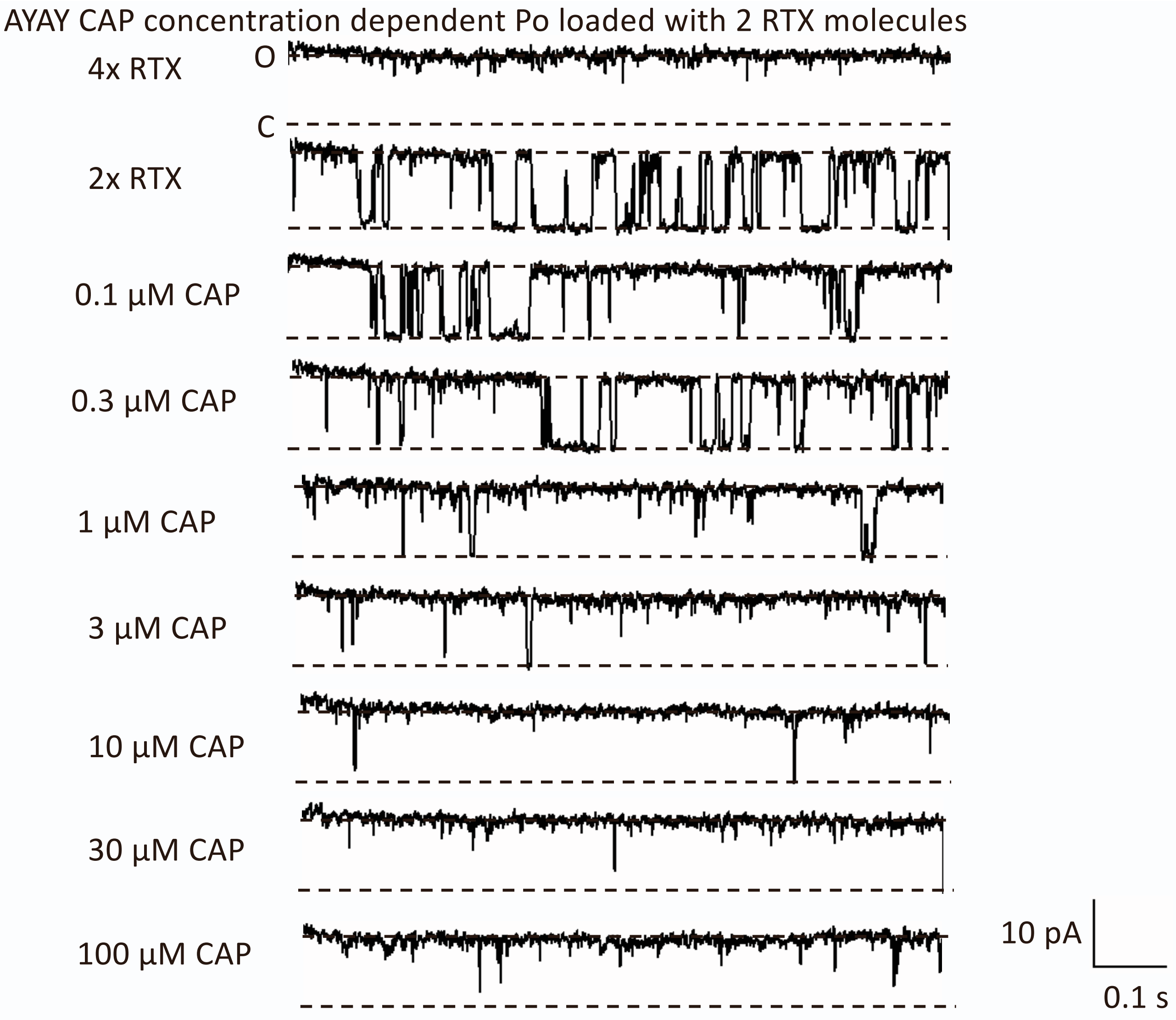

Supplementary Figure 2a

YAYA CAP concentration dependent Po loaded with 2 RTX molecules

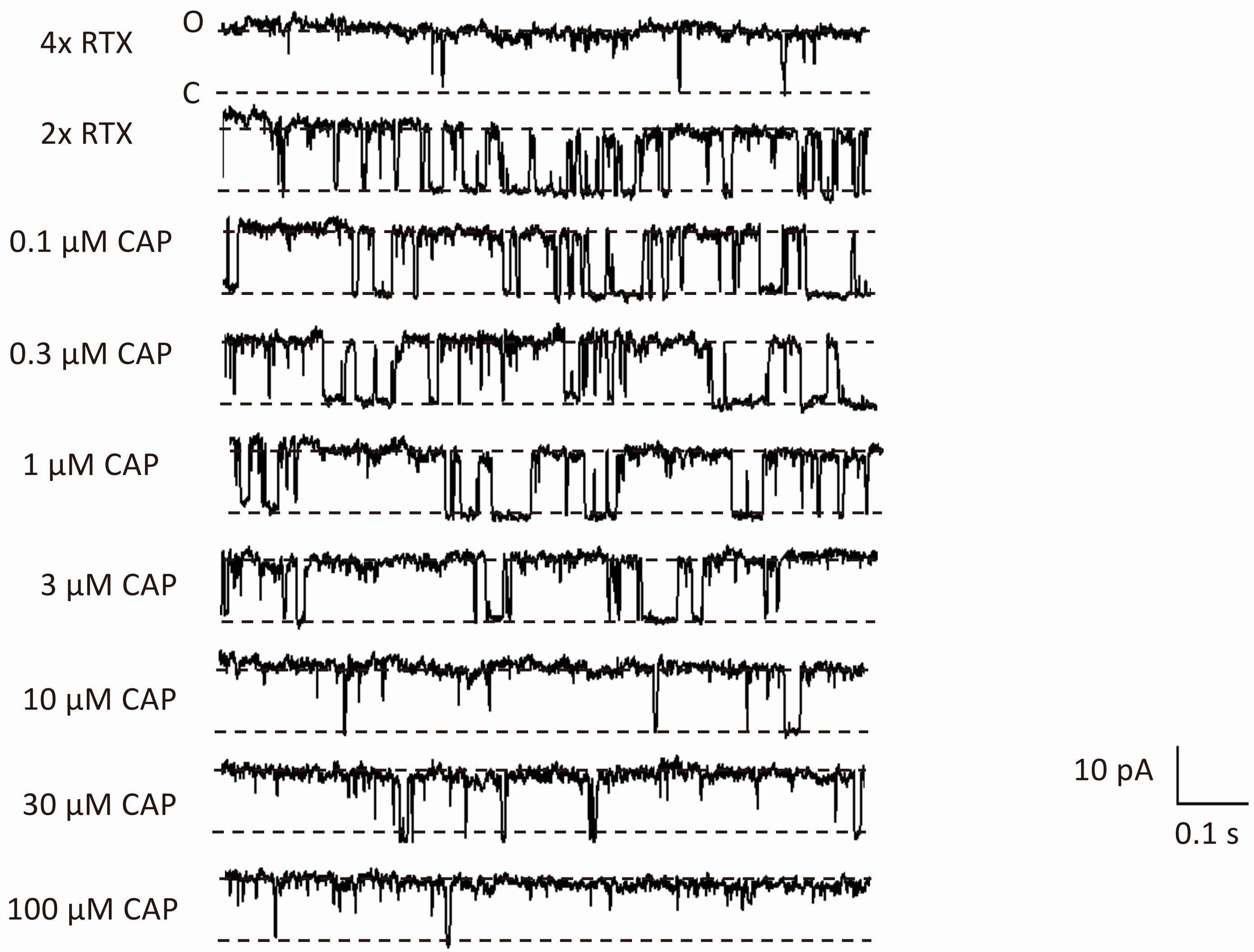

Supplementary Figure 2b

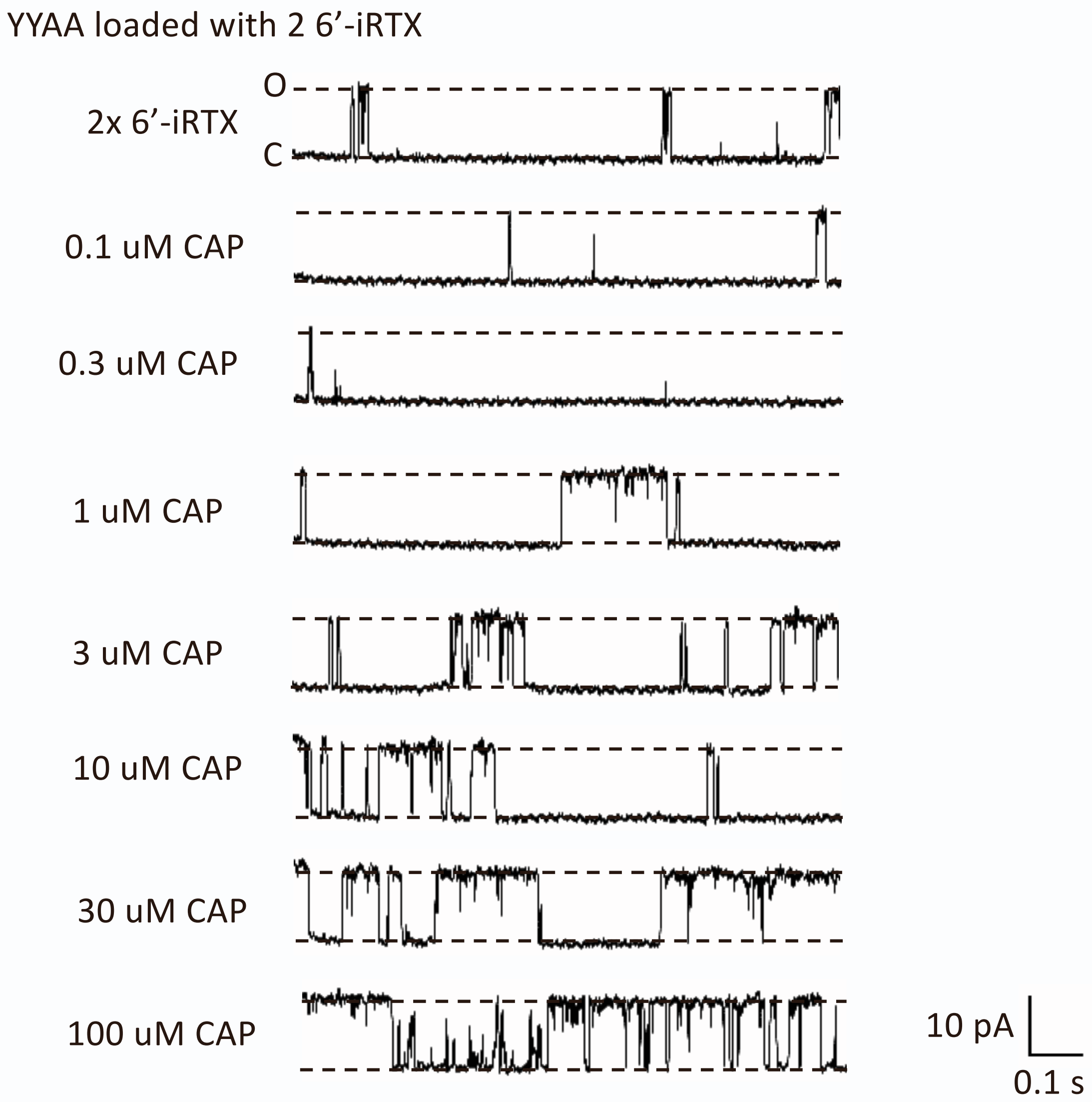

Supplementary Figure 3a

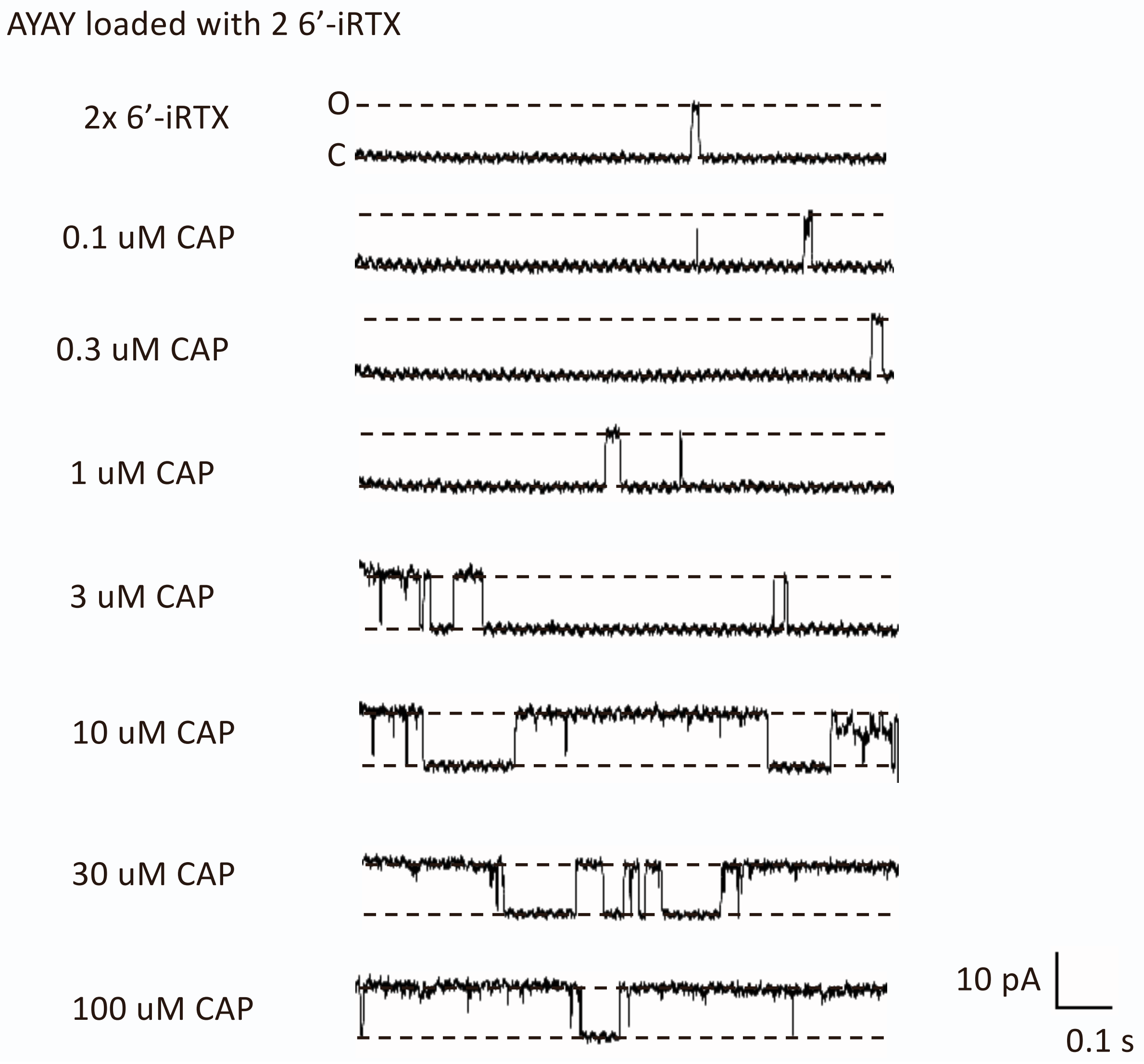

Supplementary Figure 3b

YAYA with 2x 6’-iRTX

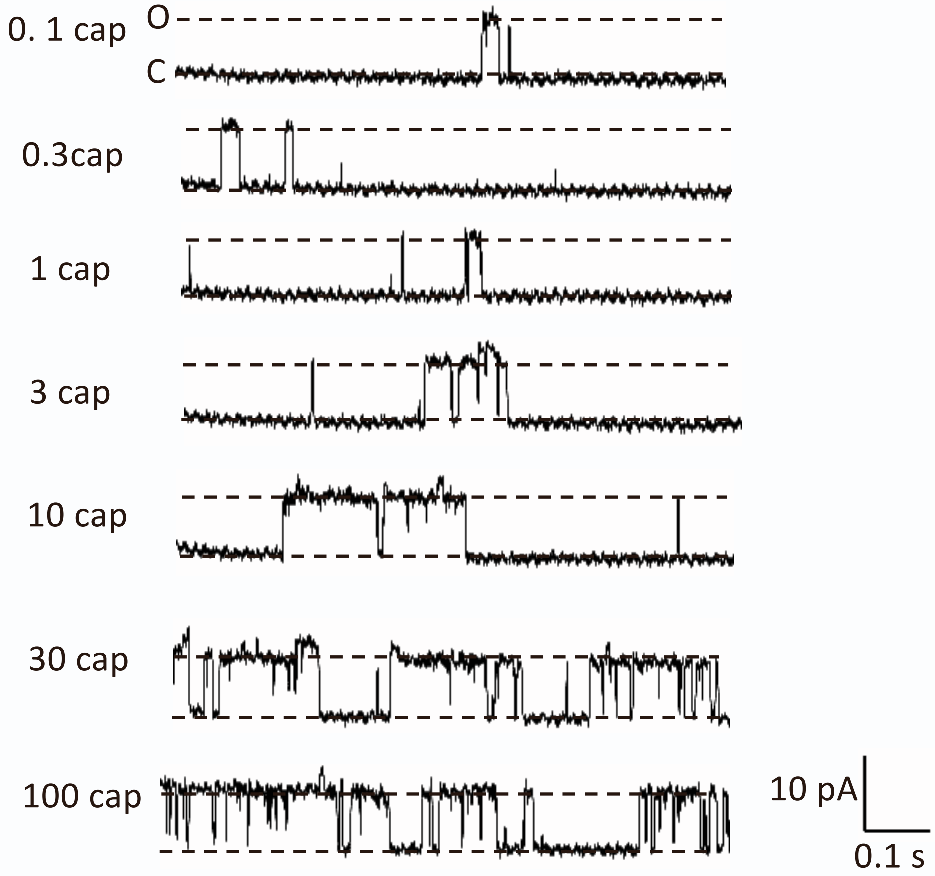

Supplementary Figure 3c

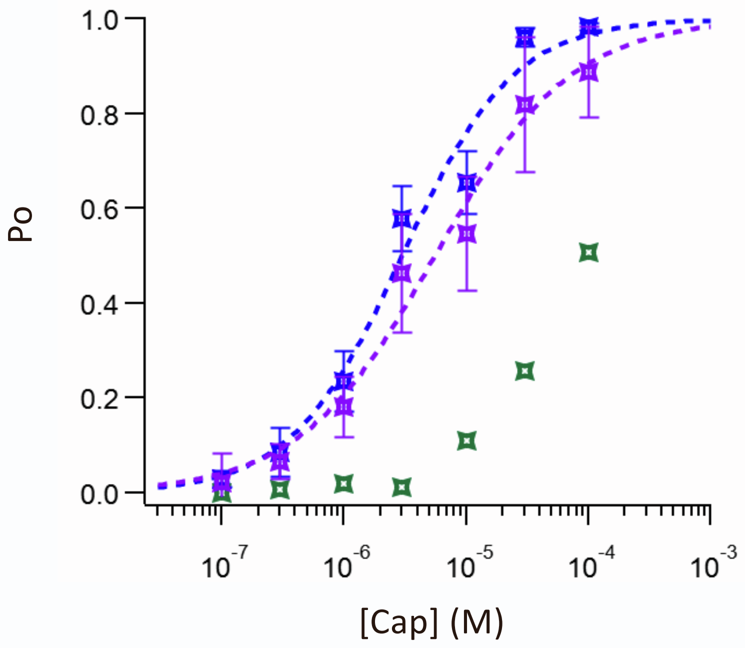

After excluding the outlier recording,
n = 4, EC50 2.99 μM, Hill slope 0.95

Before excluding the outlier recording,
n = 5, EC50 5.55 μM, Hill slope 0.95

One AYAY recording excluded as an outlier

Supplementary Figure 4

Comparison between classic MWC model with Position effect model in Figure 3 (left) and Figure 4 (right)

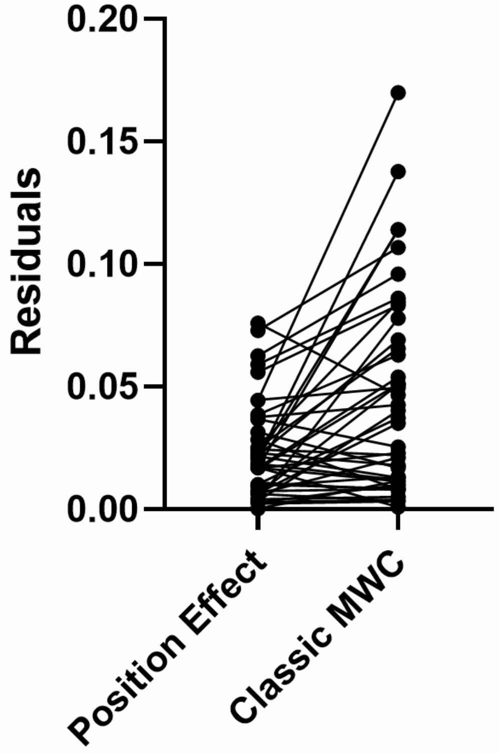

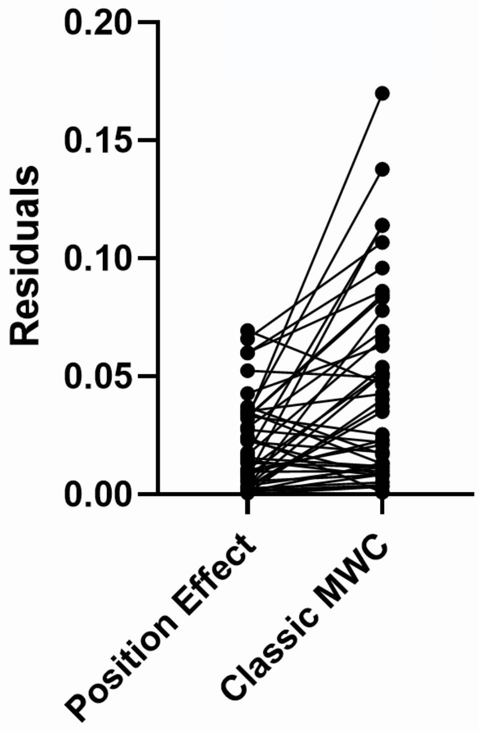

Supplementary Figure 5

Table Analyzed	Data in Figure 1B	AYAY vs YYAA						
Two-way ANOVA	Ordinary							
Alpha	0.05							
Source of Variation	% of total variation	P value	P value summary	Significant?				
Interaction	0.4622	0.4235	ns	No				
Row Factor	72.11	<0.0001	****	Yes				
Column Factor	0.783	0.0046	**	Yes				
ANOVA table	SS (Type III)	DF	MS	F (DFn, DFd)	P value			
Interaction	0.0641	5	0.01282	F (5, 84) = 0.9990	P=0.4235			
Row Factor	10	5	2	F (5, 84) = 155.9	P<0.0001			
Column Factor	0.1086	1	0.1086	F (1, 84) = 8.462	P=0.0046			
Residual	1.078	84	0.01283					
Difference between column means								
Predicted (LS) mean of AYAY	0.4917							
Predicted (LS) mean of YYAA	0.4141							
Difference between predicted means	0.07767							
SE of difference	0.0267							
95% CI of difference	0.02457 to 0.1308							
Compare each cell mean with the other cell mean in that row								
Number of families	1							
Number of comparisons per family	6							
Alpha	0.05							
Sidak's multiple comparisons test	Mean Diff.	95.00% CI of diff.	Significant?	Summary	usted P Value			
AYAY - YYAA								
1e-07	-0.0004735	-0.1767 to 0.1758	No	ns	>0.9999			
3e-07	0.03142	-0.1448 to 0.2076	No	ns	0.9975			
1e-06	0.08885	-0.08738 to 0.2651	No	ns	0.6914			
1e-05	0.08393	-0.09230 to 0.2602	No	ns	0.7436			
3e-05	0.1913	0.01508 to 0.3675	Yes	*	0.0263			
0.0001	0.07101	-0.1052 to 0.2472	No	ns	0.8615			
Test details	Mean 1	Mean 2	Mean Diff.	SE of diff.	N1	N2	t	DF
AYAY - YYAA								
1e-07	0.02933	0.0298	-0.0004735	0.0654	1	1	0.007239	84
3e-07	0.08599	0.05457	0.03142	0.0654	1	1	0.4804	84
1e-06	0.2365	0.1477	0.08885	0.0654	1	1	1.358	84
1e-05	0.6555	0.5716	0.08393	0.0654	1	1	1.283	84
3e-05	0.9599	0.7686	0.1913	0.0654	1	1	2.925	84
0.0001	0.9832	0.9122	0.07101	0.0654	1	1	1.086	84

Table Analyzed	Data in Figure 1B	YAYA vs YYAA						
Two-way ANOVA	Ordinary							
Alpha	0.05							
Source of Variation	% of total variation	P value	P value summary	Significant?				
Interaction	0.8131	0.1608	ns	No				
Row Factor	76.16	<0.0001	****	Yes				
Column Factor	1.951	<0.0001	****	Yes				
ANOVA table	SS (Type III)	DF	MS	F (DFn, DFd)	P value			
Interaction	0.123	5	0.0246	F (5, 90) = 1.628	P=0.1608			
Row Factor	11.52	5	2.305	F (5, 90) = 152.4	P<0.0001			
Column Factor	0.2951	1	0.2951	F (1, 90) = 19.52	P<0.0001			
Residual	1.361	90	0.01512					
Difference between column means								
Predicted (LS) mean of YAYA	0.5321							
Predicted (LS) mean of YYAA	0.4141							
Difference between predicted means	0.1181							
SE of difference	0.02672							
95% CI of difference	0.06497 to 0.1711							
Compare each cell mean with the other cell mean in that row								
Number of families	1							
Number of comparisons per family	6							
Alpha	0.05							
Sidak's multiple comparisons test	Mean Diff.	95.00% CI of diff.	Significant?	Summary	Adjusted P Value			
YAYA - YYAA								
1e-07	-0.01221	-0.1883 to 0.1639	No	ns	>0.9999			
3e-07	0.1201	-0.05599 to 0.2961	No	ns	0.3524			
1e-06	0.1271	-0.04901 to 0.3031	No	ns	0.2894			
1e-05	0.2245	0.04846 to 0.4006	Yes	**	0.0055			
3e-05	0.1809	0.004876 to 0.3570	Yes	*	0.0408			
0.0001	0.06793	-0.1081 to 0.2440	No	ns	0.8845			
Test details	Mean 1	Mean 2	Mean Diff.	SE of diff.	N1	N2	t	DF
YAYA - YYAA								
1e-07	0.0176	0.0298	-0.01221	0.06545	1	1	0.1865	90
3e-07	0.1746	0.05457	0.1201	0.06545	1	1	1.835	90
1e-06	0.2747	0.1477	0.1271	0.06545	1	1	1.941	90
1e-05	0.7961	0.5716	0.2245	0.06545	1	1	3.431	90
3e-05	0.9495	0.7686	0.1809	0.06545	1	1	2.765	90
0.0001	0.9801	0.9122	0.06793	0.06545	1	1	1.038	90

Table Analyzed	Data in Figure 1C	YYAA vs YAYA						
Two-way ANOVA	Ordinary							
Alpha	0.05							
Source of Variation	% of total variation	P value	P value summary	Significant?				
Interaction	1.259	0.9387	ns	No				
Row Factor	47.99	<0.0001	****	Yes				
Column Factor	2.046	0.1621	ns	No				
ANOVA table	SS	DF	MS	F (DFn, DFd)	P value			
Interaction	0.04894	5	0.009787	F (5, 48) = 0.2481	P=0.9387			
Row Factor	1.866	5	0.3731	F (5, 48) = 9.459	P<0.0001			
Column Factor	0.07953	1	0.07953	F (1, 48) = 2.016	P=0.1621			
Residual	1.893	48	0.03945					
Difference between column means								
Mean of YYAA	0.757							
Mean of YAYA	0.8298							
Difference between means	-0.07281							
SE of difference	0.05128							
95% CI of difference	-0.1759 to 0.03029							
Compare each cell mean with the other cell mean in that row								
Number of families	1							
Number of comparisons per family	6							
Alpha	0.05							
Sidak's multiple comparisons test	Mean Diff.	95.00% CI of diff.	Significant?	Summary	djusted P Value			
YYAA - YAYA								
1e-07	-0.09134	-0.4360 to 0.2533	No	ns	0.978			
3e-07	-0.09337	-0.4380 to 0.2513	No	ns	0.9755			
1e-06	-0.168	-0.5126 to 0.1767	No	ns	0.7123			
1e-05	-0.07544	-0.4201 to 0.2692	No	ns	0.9918			
3e-05	0.009542	-0.3351 to 0.3542	No	ns	>0.9999			
0.0001	-0.01834	-0.3630 to 0.3263	No	ns	>0.9999			
Test details	Mean 1	Mean 2	Mean Diff.	SE of diff.	N1	N2	t	DF
YYAA - YAYA								
1e-07	0.5125	0.6038	-0.09134	0.1256	1	1	0.7271	48
3e-07	0.568	0.6614	-0.09337	0.1256	1	1	0.7433	48
1e-06	0.614	0.782	-0.168	0.1256	1	1	1.337	48
1e-05	0.8753	0.9508	-0.07544	0.1256	1	1	0.6006	48
3e-05	0.9956	0.986	0.009542	0.1256	1	1	0.07597	48
0.0001	0.9763	0.9947	-0.01834	0.1256	1	1	0.146	48

Table Analyzed	Data in Figure 1C	YYAA vs AYAY						
Two-way ANOVA	Ordinary							
Alpha	0.05							
Source of Variation	% of total variation	P value	P value summary	Significant?				
Interaction	2.077	0.8164	ns	No				
Row Factor	50.84	<0.0001	****	Yes				
Column Factor	2.035	0.1474	ns	No				
ANOVA table	SS	DF	MS	F (DFn, DFd)	P value			
Interaction	0.07828	5	0.01566	F (5, 48) = 0.4427	P=0.8164			
Row Factor	1.916	5	0.3832	F (5, 48) = 10.84	P<0.0001			
Column Factor	0.0767	1	0.0767	F (1, 48) = 2.169	P=0.1474			
Residual	1.698	48	0.03537					
Difference between column means								
Mean of YYAA	0.757							
Mean of AYAY	0.8285							
Difference between means	-0.07151							
SE of difference	0.04856							
95% CI of difference	-0.1691 to 0.02612							
Compare each cell mean with the other cell mean in that row								
Number of families	1							
Number of comparisons per family	6							
Alpha	0.05							
Sidak's multiple comparisons test	Mean Diff.	95.00% CI of diff.	Significant?	Summary	Adjusted P Value			
YYAA - AYAY								
1e-07	-0.07395	-0.4003 to 0.2524	No	ns	0.9902			
3e-07	-0.06044	-0.3868 to 0.2659	No	ns	0.9967			
1e-06	-0.2209	-0.5473 to 0.1054	No	ns	0.3505			
1e-05	-0.06127	-0.3876 to 0.2651	No	ns	0.9964			
3e-05	0.004623	-0.3217 to 0.3310	No	ns	>0.9999			
0.0001	-0.01709	-0.3434 to 0.3093	No	ns	>0.9999			
Test details	Mean 1	Mean 2	Mean Diff.	SE of diff.	N1	N2	t	DF
YYAA - AYAY								
1e-07	0.5125	0.5865	-0.07395	0.1189	1	1	0.6218	48
3e-07	0.568	0.6284	-0.06044	0.1189	1	1	0.5082	48
1e-06	0.614	0.8349	-0.2209	0.1189	1	1	1.857	48
1e-05	0.8753	0.9366	-0.06127	0.1189	1	1	0.5152	48
3e-05	0.9956	0.9909	0.004623	0.1189	1	1	0.03887	48
0.0001	0.9763	0.9934	-0.01709	0.1189	1	1	0.1437	48

Table Analyzed	Data in Figure 1D	YYAA vs AYAY						
Two-way ANOVA	Ordinary							
Alpha	0.05							
Source of Variation	% of total variation	P value	P value summary	Significant?				
Interaction	3.297	0.1352	ns	No				
Row Factor	67.6	<0.0001	****	Yes				
Column Factor	2.022	0.015	*	Yes				
ANOVA table	SS (Type III)	DF	MS	F (DFn, DFd)	P value			
Interaction	0.146	6	0.02434	F (6, 56) = 1.712	P=0.1352			
Row Factor	2.994	6	0.499	F (6, 56) = 35.10	P<0.0001			
Column Factor	0.08958	1	0.08958	F (1, 56) = 6.300	P=0.0150			
Residual	0.7962	56	0.01422					
Difference between column means								
Predicted (LS) mean of YYAA	0.1512							
Predicted (LS) mean of AYAY	0.2242							
Difference between predicted means	-0.07302							
SE of difference	0.02909							
95% CI of difference	-0.1313 to -0.01474							
Compare each cell mean with the other cell mean in that row								
Number of families	1							
Number of comparisons per family	7							
Alpha	0.05							
Sidak's multiple comparisons test	Mean Diff.	95.00% CI of diff.	Significant?	Summary	Adjusted P Value			
YYAA - AYAY								
1e-07	0.001485	-0.2128 to 0.2158	No	ns	>0.9999			
3e-07	0.008467	-0.2059 to 0.2228	No	ns	>0.9999			
1e-06	0.01694	-0.1974 to 0.2313	No	ns	>0.9999			
3e-06	-0.02924	-0.2436 to 0.1851	No	ns	0.9998			
1e-05	-0.09114	-0.3055 to 0.1232	No	ns	0.8554			
3e-05	-0.182	-0.3964 to 0.03229	No	ns	0.1413			
0.0001	-0.2356	-0.4499 to -0.02131	Yes	*	0.0234			
Test details	Mean 1	Mean 2	Mean Diff.	SE of diff.	N1	N2	t	DF
YYAA - AYAY								
1e-07	0.009782	0.008297	0.001485	0.07697	1	1	0.01929	56
3e-07	0.02053	0.01206	0.008467	0.07697	1	1	0.11	56
1e-06	0.03171	0.01477	0.01694	0.07697	1	1	0.2201	56
3e-06	0.05741	0.08665	-0.02924	0.07697	1	1	0.3799	56
1e-05	0.128	0.2191	-0.09114	0.07697	1	1	1.184	56
3e-05	0.3838	0.5659	-0.182	0.07697	1	1	2.365	56
0.0001	0.427	0.6626	-0.2356	0.07697	1	1	3.061	56

Table Analyzed	Data in Figure 1D	YYAA vs AYAY						
Two-way ANOVA	Ordinary							
Alpha	0.05							
Source of Variation	% of total variation	P value	P value summary	Significant?				
Interaction	2.139	0.5126	ns	No				
Row Factor	51.19	<0.0001	****	Yes				
Column Factor	4.822	0.0009	***	Yes				
ANOVA table	SS (Type III)	DF	MS	F (DFn, DFd)	P value			
Interaction	0.1247	6	0.02079	F (6, 70) = 0.8822	P=0.5126			
Row Factor	2.985	6	0.4976	F (6, 70) = 21.12	P<0.0001			
Column Factor	0.2813	1	0.2813	F (1, 70) = 11.94	P=0.0009			
Residual	1.649	70	0.02356					
Difference between column means								
Predicted (LS) mean of YYAA	0.1512							
Predicted (LS) mean of YAYA	0.2739							
Difference between predicted means	-0.1227							
SE of difference	0.03553							
95% CI of difference	-0.1936 to -0.05189							
Compare each cell mean with the other cell mean in that row								
Number of families	1							
Number of comparisons per family	7							
Alpha	0.05							
Sidak's multiple comparisons test	Mean Diff.	95.00% CI of diff.	Significant?	Summary	Adjusted P Value			
YYAA - YAYA								
1e-07	-0.02165	-0.2814 to 0.2381	No	ns	>0.9999			
3e-07	-0.02446	-0.2842 to 0.2353	No	ns	>0.9999			
1e-06	-0.07143	-0.3312 to 0.1883	No	ns	0.9847			
3e-06	-0.1064	-0.3662 to 0.1534	No	ns	0.8802			
1e-05	-0.2157	-0.4755 to 0.04404	No	ns	0.1608			
3e-05	-0.2	-0.4598 to 0.05974	No	ns	0.2312			
0.0001	-0.2195	-0.4793 to 0.04023	No	ns	0.1465			
Test details	Mean 1	Mean 2	Mean Diff.	SE of diff.	N1	N2	t	DF
YYAA - YAYA								
1e-07	0.009782	0.03143	-0.02165	0.094	1	1	0.2303	70
3e-07	0.02053	0.04498	-0.02446	0.094	1	1	0.2602	70
1e-06	0.03171	0.1031	-0.07143	0.094	1	1	0.7599	70
3e-06	0.05741	0.1638	-0.1064	0.094	1	1	1.132	70
1e-05	0.128	0.3437	-0.2157	0.094	1	1	2.295	70
3e-05	0.3838	0.5839	-0.2	0.094	1	1	2.128	70
0.0001	0.427	0.6465	-0.2195	0.094	1	1	2.336	70

Column B	Classic MWC	
vs.	vs.	
Column A	Position Effect	
Wilcoxon matched-pairs signed rank test		
P value		0.0006
Exact or approximate P value?	Exact	
P value summary	***	
Significantly different (P < 0.05)?	Yes	
One- or two-tailed P value?	Two-tailed	
Sum of positive, negative ranks	750.0 , -196.0	
Sum of signed ranks (W)		554
Number of pairs		43
Number of ties (ignored)		0
Median of differences		
Median		0.007248
How effective was the pairing?		
rs (Spearman)		0.5447
P value (one tailed)	<0.0001	
P value summary	****	
Was the pairing significantly effective?	Yes	

Column B	Classic MWC	
vs.	vs.	
Column A	Position Effect	
Wilcoxon matched-pairs signed rank test		
P value		0.0002
Exact or approximate P value?	Exact	
P value summary	***	
Significantly different (P < 0.05)?	Yes	
One- or two-tailed P value?	Two-tailed	
Sum of positive, negative ranks	767.0 , -179.0	
Sum of signed ranks (W)		588
Number of pairs		43
Number of ties (ignored)		0
Median of differences		
Median		0.01077
How effective was the pairing?		
rs (Spearman)		0.4773
P value (one tailed)		0.0006
P value summary	***	
Was the pairing significantly effective?	Yes	

Figure 1B					
	EC50 (mean ± SD)	Hill slope (mean ± SD)	n	Max	Base
YYAA	7.31 ±0.13 µM	0.87 ± 0.01	12	1 (forced)	0 (forced)
YAYA	2.42 ± 0.22 µM	0.97 ± 0.08	5	1 (forced)	0 (forced)
AYAY	2.99 ± 0.53 µM	0.95 ± 0.14	4	1 (forced)	0 (forced)
Figure 1C					
	EC50 (mean ± SD)	Hill slope (mean ± SD)	n		
YYAA	2.42 ±0.65 µM	0.93 ± 0.15	5	1 (forced)	0.47
YAYA	1.13 ±0.07 µM	0.95±0.04	5	1 (forced)	0.57
AYAY	0.97 ± 0.28 µM	0.94 ± 0.20	5	1 (forced)	0.55
Figure 1D					
	EC50 (mean ± SD)	Hill slope (mean ± SD)	n		
YYAA	14.77 ± 1.59 µM	2.72 ± 0.55	4	0.03	0.43
YAYA	10.25 ± 1.82 µM	1.24 ± 0.24	8	0.04	0.7
AYAY	14.61 ± 1.65 µM	1.95 ± 0.35	6	0.02	0.68

Figure 3C						
	a	a'	L (with 6'-iRTX)	L (with RTX)	K	f
YYAA	0.83		0.01	0.92		
YAYA		1.37	0.03	1.38	$4.75x10^{4}$ M^{-1}	10.35
AYAY			0.02	1.35		
Classic MWC			0.02	1.16	5.83x104 M-1	10.42

Figure 4C						
	b	b'	L (with 6'-iRTX)	L (with RTX)	K	f
YYAA	1.07		0.02	0.94		
YAYA		2.18	0.02	1.32	$4.61x10^{4}$ M^{-1}	7.77
AYAY			0.02	1.28		
Classic MWC			0.02	1.16	5.83x104 M-1	10.42

	Figure 5	
MWC model	f	15.69
	L	0.01
2f model	f1	10.32
	f2	219.68
	L	0.01

YYAA CAP resposne

cap concentration / M	3E-08	1E-07	3E-07	0.000001	0.00001	0.00003	0.0001
	0.00116	0.00132	0.001683	0.075688	0.432355	0.797662	0.67483
	0.01829	0.042395	0.046242	0.101097	0.609344	0.738218	0.909935
	0.02246	0.055506	0.121504	0.20198	0.890999	0.803195	0.989712
	0.00608	0.042125	0.089885	0.018502	0.323397	0.43893	0.91552
	0.0025	0.007667	0.023282	0.36198	0.466941	0.634538	0.95577
		0.002211	0.003331	0.132323	0.607494	0.990124	0.965974
Po		0.004194	0.121493	0.065026	0.680081	0.903366	0.871221
		0.000705	0.029118	0.217594	0.457859	0.791083	0.980562
				0.036775	0.6211	0.834577	0.946127
				0.013542	0.381746	0.813236	
				0.365963	0.729234	0.443383	
				0.181382	0.658433	0.878361	
						0.924557	

YAYA CAP resposne

cap concentration / M	1E-07	3E-07	0.000001	0.000003	0.00001	0.00003	0.0001
	0.001465	0.004298	0.118171	0.504027	0.939473	0.996068	0.992251
	0.003498	0.123485	0.225567	0.422636	0.61102	0.941844	0.980643
Po	0.022952	0.225238	0.218257	0.798635	0.802688	0.997809	0.996706
	0.017854	0.02253	0.197567	0.248744	0.691452	0.81919	0.935415
	0.042208	0.497677	0.613994	0.745865	0.935903	0.992571	0.995534

AYAY CAP resposne

cap concentration / M	1E-07	3E-07	0.000001	0.000003	0.00001	0.00003	0.0001
	0.061226	0.180894	0.158339	0.669647	0.526443	0.973789	0.99663413
	0.023823	0.070584	0.362317	0.501933	0.617259	0.967113	0.978368625
Po	0.002939	0.006483	0.188846	0.426041	0.63376	0.902459	0.968404667
	0.000337	0.006471	0.020092	0.011191	0.110386	0.257236	0.507972924
	0.325531			0.711391	0.844575	0.996079	0.989365964

YYAA with RTX

cap concentration / M	cell 1	cell 2	cell 3	cell 4	cell 5
0	0.49842	0.487785	0.107985		0.716231
0.0000001	0.71584	0.474795	0.108695		0.750652
0.0000003	0.87048	0.439731	0.114172		0.84759
0.000001	0.89901	0.578636	0.201721		0.776695
0.00001	0.97072	0.932182	0.696173	0.801901	0.975632
0.00003	0.99738	0.993018		0.993072	0.998747
0.0001	0.99824	0.987928	0.982719	0.913617	0.99914

YAYA with RTX

cap concentration / M	cell 1	cell 2	cell 3	cell 4	cell 5
0	0.604121	0.189327	0.710825	0.937438	0.420273
0.0000001	0.656735	0.19536	0.710615	0.962873	0.493571
0.0000003	0.701432	0.426661	0.638907	0.969501	0.570313
0.000001	0.824363	0.730462	0.766204	0.985209	0.603599
0.000003	0.879815	0.777065	0.901907	0.972733	0.814442
0.00001	0.992118	0.818747	0.970062	0.996627	0.976245
0.00003	0.958433	0.992473	0.989679	0.998762	0.99071
0.0001	0.99039	0.995668	0.993592	0.998639	0.995032

AYAY with RTX

cap concentration / M	cell 1	cell 2	cell 3	cell 4	cell 5
0	0.691845	0.595336	0.767823	0.432273	0.327849
0.0000001	0.691887	0.60989	0.856322	0.425728	0.348426
0.0000003	0.695947	0.778167	0.608603	0.344579	0.714895
0.000001	0.781502	0.903207	0.966311	0.687934	0.835708
0.000003	0.869644	0.942702	0.974333	0.620049	0.858977
0.00001	0.960938	0.973929	0.975305	0.790927	0.981876
0.00003	0.998152	0.995628	0.997016	0.964571	0.999286
0.0001	0.99361	0.99692	0.997568	0.979565	0.999433

YYAA with 6'-iRTX

cap concentration / M	cell 1	cell 2	cell 3	cell 4	cell 5
0	0.00873	0.003999	0.02539	0.003108	0.001869
0.0000001	0.00246	0.020606	0.019061	0.005774	0.001012
0.0000003	0.00366	0.065908	0.016794	0.013435	0.002834
0.000001	0.01256	0.032805	0.054527	0.053341	0.005295
0.000003	0.05426	0.081571	0.090922		0.002879
0.00001	0.07083	0.123822	0.346912	0.05259	0.045906
0.00003	0.60895	0.403649	0.617971	0.095472	0.193125
0.0001	0.70361	0.457732	0.716091	0.132061	0.12537

YAYA with 6'-iRTX

cap concentration / M	cell 1	cell 2	cell 3	cell 4	cell 5	cell 6	cell 7	cell 8
0.0000001		0.042031	0.012028	0.025366		0.070854	0.003733	0.034557
0.0000003		0.049477	0.063847	0.018594		0.079319	0.002025	0.05663
0.000001		0.137754	0.053195	0.017201		0.029444	0.139807	0.241431
0.000003		0.108648	0.039225	0.11682		0.209297		0.345075
0.00001		0.158245	0.219271	0.188648	0.125988	0.370029	0.801439	0.542602
0.00003	0.521419	0.623337	0.202128	0.505087		0.694698		0.956524
0.0001	0.499784	0.619909	0.657618	0.581853	0.543887	0.645306		0.97726

AYAY with 6'-iRTX

cap concentration / M	cell 1	cell 2	cell 3	cell 4	cell 5	cell 6	cell 7	
0.0000001	0.016318		0.023014	0.000595	0.0007	0.007667		0.001485884
0.0000003	0.033404		0.002062	0.00071	0.001072	0.013476		0.021630435
0.000001	0.029399		0.011638	0.008332	0.003058	0.012434		0.02374756
0.000003	0.082403		0.023916	0.058506	0.067935	0.189653		0.097502979
0.00001	0.108437	0.394773	0.064238	0.206713	0.033504	0.172927		0.553439599
0.00003	0.350716	0.521583	0.65554	0.479749	0.465104	0.617938		0.870441125
0.0001	0.637644	0.62104	0.736006	0.490889	0.351791	0.809765		0.991088894

AAAA	YAAA	YYAA	AYAY	YAYA	YYYA	YYYY
0.003079	0.052774	0.453602	0.691845	0.604121	0.989044	0.9984
0.005536	0.232957	0.724096	0.595336	0.189327	0.998392	0.998152
0.002638	0.131032	0.4369	0.767823	0.710825	0.998437	0.996802
0.002092	0.063132	0.848706	0.432273	0.937438	0.9907	0.996618
0.007779		0.107985	0.327849	0.420273	0.993483	
		0.498422	0.966859	0.693723		
		0.487785	0.951765	0.945481		
		0.716231	0.929844	0.992585		
			0.690101	0.877653		
			0.921346	0.436193		
