# Supplementary material for: How much does TRPV1 deviate from an ideal MWC-type protein?
Source: Biophys J. 2024 Apr 6;123(14):2136–44. doi: 10.1016/j.bpj.2024.04.005 (PMC11309965; doi:10.1016/j.bpj.2024.04.005)
Supplement: Document S2. Article plus supporting material [file mmc2.pdf]

Biophysical Journal
Article

Biophysical Society

How much does TRPV1 deviate from an ideal MWC-type protein?

Shisheng Li[1] and Jie Zheng[1,*]
[1]Department of Physiology and Membrane Biology, University of California at Davis, School of Medicine, Davis, California

ABSTRACT Many ion channels are known to behave as an allosteric protein, coupling environmental stimuli captured by specialized sensing domains to the opening of a central pore. The classic Monod-Wyman-Changeux (MWC) model, originally proposed to describe binding of gas molecules to hemoglobin, has been widely used as a framework for analyzing ion channel gating. Here, we address the issue of how accurately the MWC model predicts activation of the capsaicin receptor TRPV1 by vanilloids. Taking advantage of a concatemeric design that makes it possible to lock TRPV1 in states with zero to four bound vanilloid molecules, we showed quantitatively that the overall gating behavior is satisfactorily predicted by the MWC model. There is, however, a small yet detectable subunit position effect: ligand binding to two kitty-corner subunits is 0.3–0.4 kcal/mol more effective in inducing opening than binding to two neighbor subunits. This difference—less than 10% of the overall energetic contribution from ligand binding—might be due to the restriction on subunit arrangement imposed by the planar membrane; if this is the case, then the position effect is not expected in hemoglobin, in which each subunit is related equivalently to all the other subunits.

SIGNIFICANCE The Monod-Wyman-Changeux (MWC) model, proposed more than 50 years ago, is elegantly simple yet powerful in predicting the behavior of allosteric proteins like hemoglobin. Its prediction power for ion channel gating has been beautifully demonstrated in the studies of BK channels. Our present work aims to determine how accurately the MWC model predicts TRPV1 activation induced by vanilloids. Our findings support the notion that the evolutionary drive upon allosteric proteins applies generally to multi-subunit proteins including ion channels.

INTRODUCTION

In the landmark study of neuronal action potential, Hodgkin and Huxley found that the entities controlling transmembrane conductance for sodium and potassium ions—which are now known as voltage-gated sodium (Nav) channels and voltage-gated potassium (Kv) channels—operate with high voltage sensitivities (1). In their empirical equations describing the voltage dependence of sodium and potassium conductance, this high sensitivity is reflected by the exponents assigned to the probability terms. Modern expansions of the H&H ideas incorporating knowledge of Nav and Kv channel structures reveal that high voltage sensitivity is partially rooted in the highly cooperative nature of voltage-dependent activation. In the 1990s, Zagotta, Hoshi, and Aldrich (2) and Schoppa and Sigworth (3) identified a late cooperative transition that would be needed in an otherwise independent subunit gating scheme to satisfactorily describe the course of activation in *Shaker* potassium channels. It is thought that the voltage sensors of *Shaker*, as well as many other voltage-gated ion channels, operate in a mostly independent manner, whereas some of the conformational changes in the channel pore must be concerted (4).

BK potassium channels are activated by not just transmembrane voltage but also intracellular calcium. Their activation also exhibits cooperativity (5). In a comprehensive investigation of BK channel macroscopic currents, single-channel currents, and gating currents, Horrigan, Cui, and Aldrich revealed that the voltage sensor and calcium sensor operate separately, and they both influence the channel pore opening allosterically (6–8). The kinetic model that could satisfactorily describe BK activation behaviors thus contains two branches of allosteric coupling. In each branch of the model, the four sensors (for voltage or calcium) contribute an equal amount of energy toward influencing the pore opening. Cooperativity among subunits is rooted in this joint influence of open pore stability.

Submitted October 8, 2023, and accepted for publication April 3, 2024.
*Correspondence: jzheng@ucdavis.edu
Editor: Marcel Goldschen-Ohm.
https://doi.org/10.1016/j.bpj.2024.04.005
© 2024 Biophysical Society.
This is an open access article under the CC BY license (http://creativecommons.org/licenses/by/4.0/).

This type of allosteric coupling has been previously proposed to govern another protein, the oxygen carrier hemoglobin. Hemoglobin is a protein complex made of two α subunits and two β subunits; each of these subunits contains a structurally and functionally similar binding pocket for gas molecules (9). Oxygen binding to these four sites in hemoglobin is highly cooperative (10). Monod, Wyman, and Changeux postulated that the cooperativity comes from a concerted conformational change that affects all gas binding sites equally (11). Hemoglobin serves as a carrier for gas molecules; there is no function equivalent to ion conduction in an ion channel that can be used as a direct indicator of the concerted transition. Studies of hemoglobin therefore focused on the ligand-binding process. It was proposed that there is an equal energetic contribution to the concerted transition by each gas molecule binding step and that these binding steps, per se, are independent (11). Introducing interactions at the binding steps, such as those seen in the sequential model, yields good performance but also adds complexity (12).

Equal and independent contribution to gating by each subunit is assumed for BK channels in the study by Horrigan, Cui, and Aldrich and for many other ion channels studied subsequently. The MWC-type models in general worked well in predicting channel behaviors and provided important guidance for mechanistic investigations in the following decades. Many mechanistic predictions from these studies were nicely confirmed when ion channel structures became available (4). In recent studies of the capsaicin receptor TRPV1 (13,14), we realized that a set of concatemers previously designed by Priel and colleagues (15) would allow us to lock a TRPV1 channel in each of the intermediate ligand-bound states. This and the opportunity to directly access the allosteric transition in an ion channel through current recording present a unique opportunity to directly test the various postulates in the MWC model. This is possible because the concatemers, made of various combinations of wild-type and Y511A mutant protomers, would trap a vanilloid molecule such as resiniferatoxin (RTX) when it binds to a wild-type subunit but allow it to fall off a mutant subunit. Isolating intermediate binding states for equilibrium measurements has been challenging for hemoglobin bound with small gas molecules; it is, to our best knowledge, not done with any concatemeric ion channels. In the present study, we took advantage of this powerful system to address the question of how closely TRPV1 resembles an ideal MWC-type allosteric protein.

MATERIALS AND METHODS

Molecular biology

The plasmids used in this study were made in the Priel laboratory (15). Briefly, wild-type rat TRPV1 (Y) cDNAs were joined with the Y511A mutant (A) cDNAs in various combinations: YYYY, YYYA, YYAA, YAYA, AYAY, YAAA, and AAAA. Their functional properties have been carefully tested and described in previous publications; no noticeable change in gating behavior was detected in these studies (13–15). Representative single-channel recordings for each concatemer are presented in Figs. S1–S5. Y511 locates near the entrance of the vanilloid binding pocket (16). Its long side chain points downward in the apo state; binding of a vanilloid ligand causes the side chain to flip upwards (17). In this up position, the side chain would slow down a bound ligand from exiting the pocket (14,18). We recently found that the Y512A mutation (with a smaller side chain) in the mouse TRPV1, or the equivalent Y511A mutation in the rat TRPV1, makes binding of RTX and 6′-iodoresiniferatoxin (6′-iRTX) reversible (13,14). Exit of a bound capsaicin, which has the same vanillyl headgroup as RTX but is much small in overall size, is apparently also facilitated by the mutation (19).

Cell culture

TSA201 cells (HEK293T variant from American Type Culture Collection, Manassas, VA, USA) served as the expression system for patch-clamp assays. These cells were cultivated on 25 mm glass coverslips in 30 mm dishes (from Thermo Scientific, Waltham, MA, USA) until reaching 30%–50% confluence and then transiently transfected. Transfection was carried out using Lipofectamine 2000 (Invitrogen, Carlsbad, CA, USA) 24 h prior to patch-clamp recording, following the manufacturer's guidelines. For single-channel recordings, a combination of 0.1 μg concatemer plasmid and 0.2 μg EYFP plasmid was utilized per transfection.

Chemical solutions

For inside-out patch-clamp recordings, we used symmetric bath and pipette solutions containing 140 mM NaCl, 15 mM HEPES, 2 mM EDTA (pH 7.4). Capsaicin (from Sigma-Aldrich, St. Louis, MO, USA) was dissolved in DMSO to prepare a 1 M stock and then further diluted to concentrations ranging from 0.01 to 100 μM using the bath solution. Both RTX (Alomone Labs, Jerusalem, Israel) and 6′-iRTX (Sigma-Aldrich) were dissolved in ethanol to create a 1 mM stock and subsequently diluted to a 200 nM working concentration with the bath solution. Lastly, 2-aminoethoxydiphenyl borate (Sigma-Aldrich) was dissolved in DMSO to produce a 1 M stock, which was then diluted to a 3 mM working solution.

Electrophysiology

Pipettes for patch-clamp recordings were pulled from borosilicate glass capillaries (Sutter Instrument, Novato, CA, USA) using a P-97 micropipette puller (Sutter Instrument) and fire polished to achieve resistances 8–15 MΩ for single-channel recordings. We employed an EPC 10 USB patch-clamp amplifier (Warner Instruments, Hamden, CT, USA) operated by the PatchMaster software. Sampling and filtering frequencies were set at 10 kHz and 2.25 kHz, respectively. Patch-clamp configurations were primarily inside out unless specifically stated otherwise. The holding potential began at 0 mV and proceeded at steps of +80 and −80 mV. Step durations were adjusted as required. A gravity-driven perfusion system, controlled by the Rapid Solution Changer (RSC-200, BioLogic, Seyssinet-Pariset, France), facilitated solution perfusion and changes.

Data analysis

Patch-clamp data, exported from PatchMaster in the Igor format, were analyzed using Igor Pro 8 (WaveMetrics, Portland, OR, USA). Statistical analyses were conducted in GraphPad Prism 8. Two-way ANOVA tests were done to detect difference between YYAA (ligand binding in neighboring subunits) versus YAYA or AYAY (ligand binding in kitty-corner subunits). Detailed results for the two-way ANOWA test are included in Tables S1–S8. Data are presented as mean ± SEM.

Single-channel open probabilities were determined by creating all-point histograms from single-channel current traces. For recordings from a single channel, the histogram contained two peaks, whose area represented the total time spent in the closed state, t_c, and the open state, t_o, respectively. The open probability was calculated as $Po = \frac{t_o}{t_o+t_c}$. For patches containing two channels, the histogram contained two open-state peaks corresponding to one and two channels in the open state. The areas under them, t_1 and t_2, respectively, were used to determine the open probability using the equation $Po = \frac{t_1+2\times t_2}{2\times T}$. Here, T represents the total area of the histogram. Recordings with more than two channels were discarded.

Model fitting

As an initial analysis, capsaicin responses of concatemeric channels were fitted to a Hill equation

$$Po = P_{o\_base} + \frac{(P_{o\_max} - P_{o\_base})}{1 + \left(\frac{\text{EC50}}{[Cap]}\right)^n},$$

where EC50 is the concentration for 50% activity, $[Cap]$ is the capsaicin concentration, and n is the Hill slope factor. Capsaicin responses of concatemeric channels pretreated with RTX or 6′-iRTX were further fitted to models with specific modifications of the MWC model (shown in Fig. 2). The goal of these analyses was to obtain an upper limit estimate for the free energy representing the deviation from an ideal MWC system. For these analyses, a global fitting routine was applied simultaneously to data from all three concatemers pretreated with either RTX or 6′-iRTX. The global fitting package in Igor Pro 8 was used to fit these single-channel *Po* data.

To obtain free energy estimates, we considered two extreme situations. For the first situation, we assumed that the observed position effect was solely due to differences in capsaicin binding affinity. This could be reflected by assigning a cooperativity coefficient to the first ligand-binding step in the MWC-type model for a two-site system while keeping the other parameters unchanged. Shown in Fig. 3, *A* and *B*, are equilibrium constants for all the transitions, where L_2 is the equilibrium constant for channels preloaded with two RTX or 6′-iRTX molecules, $[c]$ is the concentration of capsaicin in the perfusion solution, K is the binding affinity parameter, a and a' are the cooperativity coefficients for binding, and f is the gating parameter (fold change to L_2 as a result of each capsaicin binding step). Solving this model under the equilibrium condition yields the following equation:

$$Po = \frac{L_2 + 2afK[c]L_2 + af^2K^2[c]^2L_2}{1 + 2aK[c] + aK^2[c]^2 + L_2 + 2afK[c]L_2 + af^2K^2[c]^2L_2}.$$

Global fitting results using this model are shown in Fig. 3 *C*, where the same set of values for f and K were used and the values of L_2, a (applied to the YYAA data set), and a' (applied to the YAYA and AYAY data sets) were allowed to vary between different groups.

For the second extreme situation, we assumed that the observed position effect was solely due to a difference in the activation transition. This could be reflected by assigning a cooperativity coefficient to the first vertical gating transition (which would be carried on to the second gating transition and the first binding transition in the open state, to maintain microscopic reversibility) while keeping the other parameters unchanged. Shown in Fig. 4, *A* and *B*, are equilibrium constants for all the transitions, where individual parameters are defined in the same way as described above, and b and b' are the cooperativity coefficients for gating (ratio factor between the two gating equilibrium constants following the first and second capsaicin binding steps). Solving this model under the equilibrium condition yields the following equation:

$$Po = \frac{L_2 + 2bfK[c]L_2 + bf^2K^2[c]^2L_2}{1 + 2K[c] + K^2[c]^2 + L_2 + 2bfK[c]L_2 + bf^2K^2[c]^2L_2}.$$

Global fitting results using this model are shown in Fig. 4 *C*, where the same set of values for f and K were used and the values of L_2, b (applied to the YYAA data set), and b' (applied to the YAYA and AYAY data sets) were allowed to vary between different groups.

For comparison, a classic MWC model without a position effect was also used to fit the group data (*gray curves* in Figs. 3 *C* and 4 *C*):

$$Po = \frac{L_2 + 2fK[c]L_2 + f^2K^2[c]^2L_2}{1 + 2K[c] + K^2[c]^2 + L_2 + 2fK[c]L_2 + f^2K^2[c]^2L_2}.$$

To directly determine the position effect on free energy associated with the gating transitions, we measured the *Po* values when a different number of RTX molecules were bound to the channel. This was achieved by treating each of the YYYY, YYYA, YYAA, YAYA, AYAY, YAAA, and AAAA channels for an extended time, followed by a thorough wash to remove the reversible binding of RTX to the A subunits, as previously described (13). The following equations were used:

For AAAA (with no RTX bound): $Po = \frac{L_0}{1+L_0}$.

For YAAA (with 1 RTX bound): $Po = \frac{f_1L_0}{1+f_1L_0}$.

For YAYA and AYAY (with 2 RTX bound at diagonal sites): $Po = \frac{f_2L_0}{1+f_2L_0}$.

For YYAA (with 2 RTX bound at adjacent sites): $Po = \frac{f_1{}^2L_0}{1+f_1{}^2L_0}$.

For YYYA (with 3 RTX bound): $Po = \frac{f_1f_2L_0}{1+f_1f_2L_0}$.

For YYYY (with 4 RTX bound): $Po = \frac{f_2{}^2L_0}{1+f_2{}^2L_0}$.

In these equations, L_0 is the equilibrium constant for channels in the apo state, f_1 is the gating parameter when only one RTX is bound in the diagonal pair of the binding sites, and f_2 is the gating parameter when two RTX are bound in the diagonal pair of the binding sites. The results of global fitting of these equations simultaneously to the corresponding data sets are shown in Fig. 5 *B*.

The classic MWC model would predict for this situation the following equation: $Po = \frac{f^nL_0}{1+f^nL_0}$, where L_0 is the equilibrium constant for channels in the apo state, f is the gating parameter for each RTX bound to the channel, and n is the number of bound RTX. A prediction of this model is included in Fig. 5 *B* for comparison.

RESULTS

TRPV1 is an allosteric protein. In the absence of ligands such as capsaicin or RTX, TRPV1 can spontaneously transition from a closed state to an open state at a very low probability (with an equilibrium constant less than 0.01) (13,20). As an increasing number of agonist molecules bind to TRPV1, the equilibrium progressively shifts toward the open state. Our previous study showed that, during RTX activation of TRPV1, sequential RTX bindings shift the equilibrium nearly exponentially, suggesting that energy contributions from these RTX binding steps are approximately equal (13) as the classic MWC model postulates (11). Binding of all four subunits with RTX molecules contributes a total energy of 6.8–7.4 kcal/mol toward activation (13).

However, when we closely examined the capsaicin response curves for YAYA, AYAY, and YYAA concatemers—all producing channels with two wild-type subunits and two mutant subunits but with different subunit

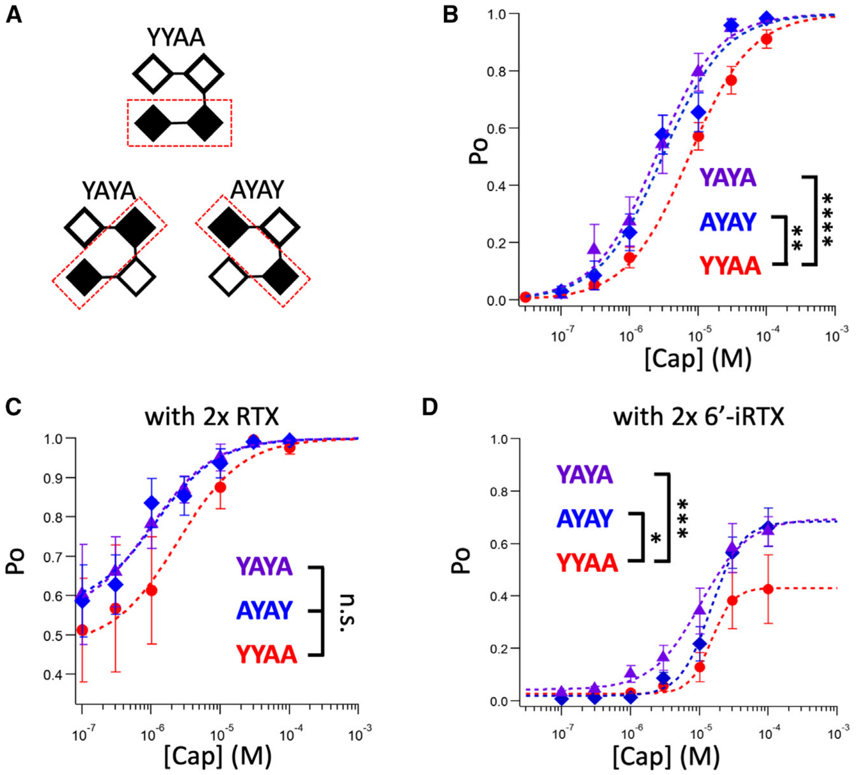

FIGURE 1 (*A*) Expected subunit positioning for YYAA, YAYA, and AYAY concatemers; an open square represents a wild-type subunit, and a filled square represents a Y511A mutant subunit. (*B*) Capsaicin concentration-dependent single-channel open probability curves for YYAA, YAYA, and AYAY fitted to a Hill equation using the following EC50 and Hill slope parameters: YYAA (*red*), 7.31 μM, 0.87, $n = 12$; AYAY (*blue*), 2.99 μM, 0.95, $n = 4$; YAYA (*black*), 2.42 μM, 0.97, $n = 5$. (*C*) Capsaicin concentration-dependent single-channel open probability curves for YYAA, YAYA, and AYAY with two preloaded RTX molecules in the wild-type protomers fitted to a Hill equation: YYAA, 2.42 μM, 0.93, $n = 5$; AYAY, 0.97 μM, 0.94, $n = 5$; YAYA, 1.13 μM, 0.95, $n = 5$. (*D*) Capsaicin concentration-dependent single-channel open probability curves for YYAA, YAYA, and AYAY with two preloaded 6′-iRTX molecules in the wild-type protomers fitted to a Hill equation: YYAA, 14.77 μM, 2.72, $n = 4$; AYAY, 14.61 μM, 1.95, $n = 6$; YAYA, 10.25 μM, 1.24, $n = 8$. Error bars represent SEM. Two-way ANOVA with Sidak's multiple comparisons test; n.s., no significance; $^{*}p < 0.05$, $^{**}p < 0.01$, $^{***}p < 0.001$, and $^{****}p < 0.0001$.

arrangements (Fig. 1 *A*)—a minor but apparent difference could be discerned. Both YAYA and AYAY (with two mutant subunits located at kitty-corners in the assembled channels) appeared to be slightly more sensitive to capsaicin than YYAA (with mutant subunits at neighbor positions) (Fig. 1 *B*). The small differences in the EC50 value, obtained from fitting with a Hill function, would translate into free energy differences of about 0.66 kcal/mol between YYAA and YAYA and 0.53 kcal/mol between YYAA and AYAY. The group data for YAYA and AYAY are significantly deviated from the group data for YYAA ($p < 0.0001$ and $p = 0.005$, respectively) (Table S1). Nonetheless, at most specific capsaicin concentrations, it was hard to confidently establish a statistically significant difference in *Po*. (One of the five recordings from AYAY yielded an abnormal right-shifted capsaicin dependence curve, which was excluded from Fig. 1 *B*. While the cause of this abnormality is unknown, there are previous reports that a concatemeric construct may sometimes fail to constrain the subunit composition. Including this outlier recording would make the difference between AYAY and YYAA even smaller [see Fig. S4].)

Given the uncertainty, we assessed the potential subunit positioning effect with a different approach. Taking advantage of the reversible binding of RTX to the A subunits and irreversible binding to the Y subunits, we first fully loaded each channel type with RTX, followed by a thorough washing. As we reported previously (13,14), this procedure yielded channels containing two Y subunits bound with RTX and two A subunits available for subsequent binding by capsaicin. We measured the channel open probability at increasing concentrations of capsaicin. As shown in Fig. 1 *C*, a slightly higher capsaicin sensitivity in YAYA and AYAY concatemeric channels was again observed, which is reflected in the uplifted concentration-dependent curves, though this apparent difference was not statistically significant ($p = 0.16$ and 0.15) (Table S2). Similar results were obtained using 6′-iRTX, an RTX derivative that is a much weaker TRPV1 agonist: a small yet statistically significant ($p = 0.0009$ and 0.015) upward shift in the capsaicin concentration-dependent curves for AYAY and YAYA could be seen (Fig. 1 *D*).

Our results indicate that, when two ligands bind to a single TRPV1 channel, the diagonal (kitty-corner) binding positions produce a higher open probability than the adjacent binding positions. This behavior is not aligned with the prediction of the classic MWC model, which assumes equality in subunit contributions (Fig. 2 *A*). To experimentally assess the magnitude of this position effect, we used a modified model incorporating distinct states for the two-ligand bound configurations, as shown in Fig. 2 *B*. In this expanded model, the increased cooperativity for the diagonal binding positions could arise either from the binding process (characterized by the *K* parameter) or the gating process (characterized by the *f* parameter). We evaluated these possibilities separately in the following two sets of experiments using

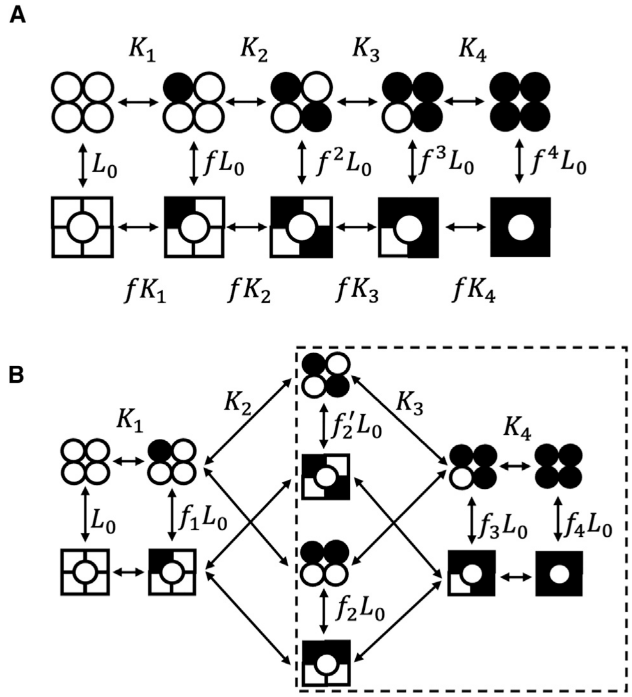

FIGURE 2 (*A*) Illustration of a classic MWC model for a tetrameric ligand-gated ion channel with four identical ligand-binding sites. L_0 represents the equilibrium constant for the apo state, and f represents the cooperative factor, which reflects the energy contribution by each ligand binding; K_1–K_4 represent the ligand association constants. (*B*) Illustration of a modified model with position effects. The dashed box shows the subsystem examined in Figs. 3 and 4.

single-channel *Po* data in response to capsaicin after the two Y subunits in each channel were preloaded with RTX or 6′-iRTX molecules (the subsystem is shown by the *dashed box* in Fig. 2 *B*).

To assess a potential subunit position effect on binding affinity, we first assumed the gating effect (f) to be independent of the position effect (Fig. 3, *A* and *B*); that is, variation in gating cooperativity was assumed to originate solely from binding, hence potentially exaggerating the binding effect. We assigned the equilibrium constant for the last binding step the same K parameter, since this step is identical for all concatemers, as can be seen in Fig. 3, *A* and *B*. However, transitioning from a two-ligand-bound state to a three-ligand-bound state might show sensitivity to the initial positions of liganded subunits, leading us to introduce a coefficient factor a or a' to the binding parameter K. In the absence of a position effect, both a and a' would be 1, as expected from independent binding. However, if a position effect existed, then a and a' would differ. As described earlier, we first treated each concatemeric channel with RTX or 6′-iRTX followed by a thorough wash; this yielded channels in which the wild-type Y subunits were occupied (*black filled symbols* in Fig. 3, *A* and *B*) and the mutant subunits available for binding by capsaicin. Global fitting of the capsaicin response data sets from all concatemers pretreated with either RTX or 6′-iRTX (Figs. 3 *C* and S5, *left*) yielded values of $a = 0.83$ and $a' = 1.37$, suggesting the presence of a position effect, albeit one that is rather small. These factors represent free energy differences from independence (a and $a' = 1$) by −0.11 and 0.19 kcal/mol, respectively. The kitty-corner bound subunit arrangement deviates more noticeably. The free energy difference between the two subunit arrangements would be $\Delta\Delta\Delta G =$ 0.30 kcal/mol. Considering that our method likely overestimated binding cooperativity, the actual difference would be smaller.

To gauge the gating effect, we used a similar approach, assuming the binding steps were independent (Fig. 4, *A* and *B*). This would attribute any binding cooperativity to gating, potentially exaggerating the gating effect. For the reason discussed earlier, we assigned the same f factor when the channel transitioned from a three-ligand-bound state to a four-ligand-bound state. We introduced a coefficient factor b or b' to the gating parameter f for transitions from a two-ligand-bound state to a three-ligand-bound state. Without a position effect, b and b' would be 1; otherwise, they would differ. Global fitting of the same data sets yielded values of $b = 1.07$ and $b' = 2.18$, corresponding to free energy deviations from equal subunit contributions (b and $b' = 1$) by 0.04 and 0.46 kcal/mol (Figs. 4 *C* and S5, *right*). Again, the kitty-corner bound subunit arrangement deviates more noticeably. The free energy difference between the two subunit arrangements would be $\Delta\Delta\Delta G =$ 0.42 kcal/mol.

Since both fitting methods could potentially skew the estimated position effect, we explored a direct approach to estimate the free energy difference associated with the position effect on gating. We have previously shown that, by fully loading each concatemer with RTX followed by thorough washing, we could obtain channels with one to four RTX-bound subunits (13). *Po* measurements from these channels reflect only the gating equilibrium of each vertical transition seen in Fig. 5 *A* without confounding effects from binding. The difference between two-ligand-bound channels could be represented by introducing just one additional degree of freedom to the classic MWC model. We assigned f_1 as the gating coefficient for a single independent binding site and hypothesized that each pair of diagonal binding sites would exhibit stronger cooperativity (f_2) than two independent sites (f_1^2). Using this model to fit the RTX-bound TRPV1 single-channel *Po* data (Fig. 5 *B*), we found f_2 to be 107% larger than f_1^2, equivalent to a free energy difference $\Delta\Delta\Delta G = 0.43$ kcal/mol. This result aligns nicely with our indirect estimates discussed earlier, suggesting that, while binding might contribute to the position effect (probably much less than 0.30 kcal/mol), gating plays a larger role, contributing approximately 0.4 kcal/mol. The position effect would favor traversing the diagonally liganded conformation over the adjacently liganded conformation in a roughly 2:1 ratio. Nonetheless, compared to

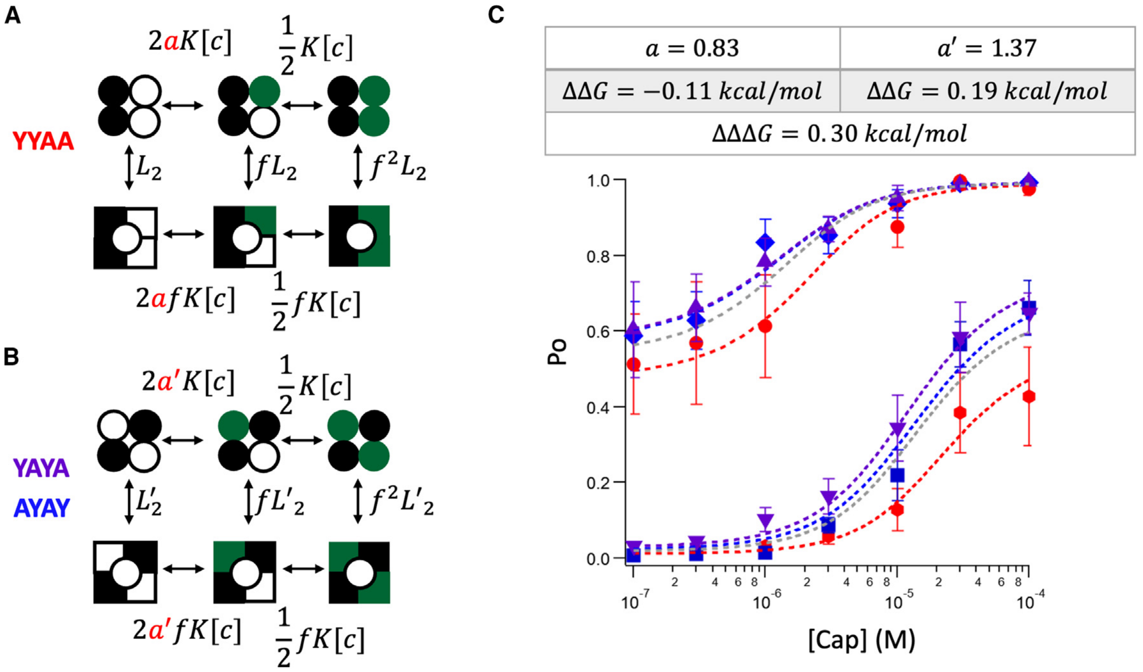

FIGURE 3 (*A* and *B*) Illustration of the models used for fitting the capsaicin-dependent *Po* data with two preloaded RTX or 6′-iRTX to test the contribution of binding to the position effect. L_2 and L_2' represent the equilibrium constants for the state with two preloaded RTX or 6′-iRTX (L_2 for YYAA, L_2' for YAYA and AYAY) before application of capsaicin; a and a' are coefficient factors for the position effect (a for YYAA, a' for YAYA and AYAY); when there is no position effect, a and a' should be 1 for independent bindings; f represents the cooperative factor, which reflects the energy contribution by each ligand binding; K represents the ligand binding affinity constant for the Y511A mutant subunit, and $[c]$ is the capsaicin concentration. (*C*) Global fitting results using the models shown in (*A*) and (*B*) with the following parameters: $a = 0.83$; $a' = 1.37$; $K = 4.8 \times 10^4\ M^{-1}$, $f = 10.3$; YYAA: L (with 6′-iRTX) = 0.01, L (with RTX) = 0.92; YAYA: L (with 6′-iRTX) = 0.03, L (with RTX) = 1.38; AYAY: L (with 6′-iRTX) = 0.02, L (with RTX) = 1.35. Deviations of the a and a' values from 1 (for independent ligand binding) represent $\Delta\Delta G$ values of −0.11 (for YYAA) and 0.19 kcal/mol (for YAYA and AYAY). For model fitting with the classic MWC model (*gray line*): $K = 5.8 \times 10^4\ M^{-1}$, $f = 10.4$, L (with 6′-iRTX) = 0.02, L (with RTX) = 1.16. Error bars represent SEM.

the 6.8–7.4 kcal/mol total energetic contribution from ligand binding to activation, the position effect is small (less than 10%).

DISCUSSION

The present paper is the third in a series of studies of TRPV1 activation by vanilloid molecules (13,14). Our collective results from these studies suggest that, as an allosteric protein, TRPV1 can exist in various conformations (closed versus open and with various numbers of ligands) at any ligand concentration. A recent structural study has captured many of these conformations, including those with two bound ligands at either neighbor or kitty-corner positions (21). It remains unclear what might contribute to the small difference in stability between these two configurations. The cryoelectron microscopy (cryo-EM) structures reveal that a vanilloid molecule bound in its binding pocket does not belong strictly to one subunit: due to the domain-swapped arrangement, the vanilloid binding pocket is formed by the S3 and S4 segments and the S4-S5 linker from one subunit together with the S5 and S6 segments from a neighbor subunit (16). Whereas capsaicin forms hydrogen bonds with the S4 segment and the S4-S5 linker of the same subunit, extensive hydrophobic interactions with the S5 and S6 segments are predicted from both the cryo-EM structures and computational modeling (22). Polar interactions between capsaicin or other ligands and the S6 segment are also predicted (23–25). However, results from our recent study and the present study showed that capsaicin binding to the four TRPV1 subunits are independent events (14), suggesting that the position effect might have a different origin (see below).

One intriguing question that arises from the present study is whether hemoglobin exhibits position differences in ligand-induced allosteric transition like what we have observed in TRPV1. To our best knowledge, no direct experimental evidence in support of such a possibility has been reported. The binding pockets for gas molecules in hemoglobin are formed within each of the four isolated gas molecule binding domains (9). These binding pockets are positioned at the vertex corners of a tetrahedron such that each pocket is related to all the other pockets nearly equally (Fig. 5 *C*, *top*). TRPV1 and other ion channels existing in a planar membrane do not share this symmetry. A vanilloid binding pocket in TRPV1 is related differently to its neighbor pockets and the kitty-corner pocket (Fig. 5 *C*, *bottom*). When two neighbor binding pockets are occupied by ligands, the channel complex lacks the rotational symmetry

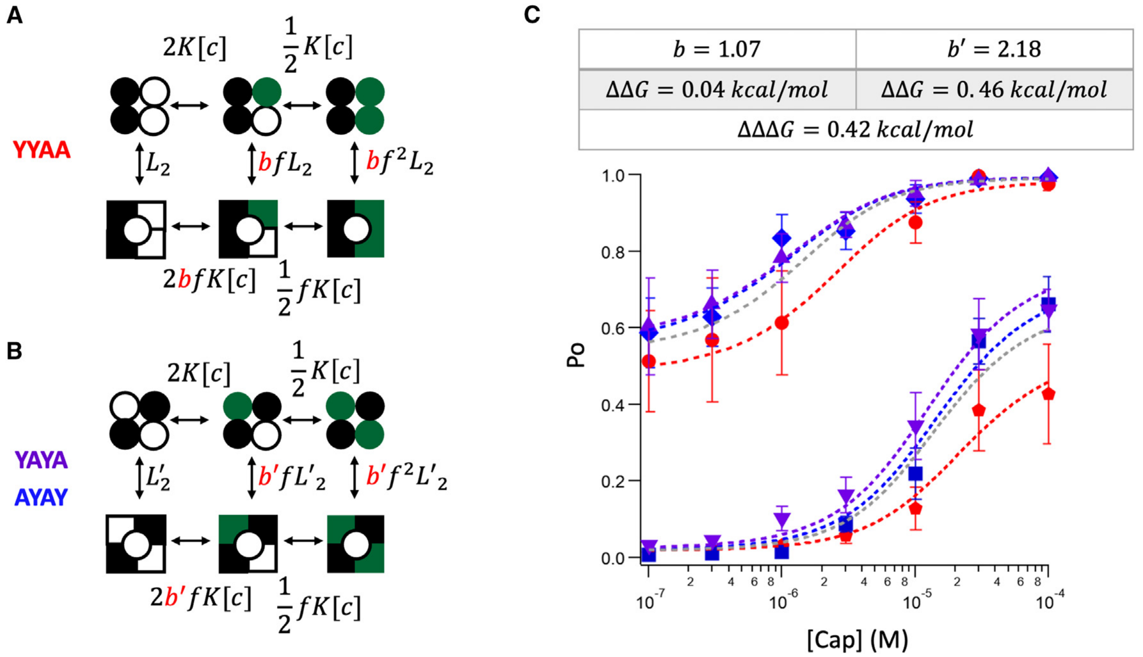

FIGURE 4 (*A* and *B*) Illustration of the models for testing the contribution of gating to the position effect. L_2 and L_2' represent the equilibrium constants for the state with two preloaded RTX or 6′-iRTX (L_2 for YYAA, L_2' for YAYA and AYAY) before application of capsaicin; b and b' are coefficient factors for the position effect (b for YYAA, b' for YAYA and AYAY); when there is no position effect, b and b' should be 1; f represents the cooperative factor, which reflects the energy contribution by each ligand binding; K represents the ligand binding affinity constant for the Y511A mutant subunit, and $[c]$ is the capsaicin concentration. (*C*) Global fitting results using the models from (*A*) and (*B*) with the following parameters: $b = 1.07$; $b' = 2.18$; $K = 4.6 \times 10^4\ \mathrm{M}^{-1}$, $f = 7.8$; YYAA: L (with 6′-iRTX) $= 0.02$, L (with RTX) $= 0.94$; YAYA: L (with 6′-iRTX) $= 0.02$, L (with RTX) $= 1.32$; AYAY: L (with 6′-iRTX) $= 0.02$, L (with RTX) $= 1.28$. Deviations of the b and b' values from 1 (no position effect) represent $\Delta\Delta G$ values of 0.04 (for YYAA) and 0.46 kcal/mol (for YAYA and AYAY). For model fitting with classic MWC model (*gray line*): $K = 5.8 \times 10^4\ \mathrm{M}^{-1}$, $f = 10.4$, L (with 6′-iRTX) $= 0.02$, L (with RTX) $= 1.16$. Error bars represent SEM.

exhibited when the kitty-corner binding pockets are occupied. Our observations showed that diagonally liganded channels have a higher probability residing in the open state (corresponding to the relaxed state in the MWC model). The situation would not be found in hemoglobin.

Despite the geometric restriction imposed by the planar membrane, a centrally located ion permeation pore surrounded by three to five structurally similar subunits or domains evolves as the overwhelmingly dominant architecture for biological ion channels (4). An obvious benefit of this architecture is the possibility for cooperative control of ion permeation by the multiple subunits or domains, which bestows a much greater sensitivity compared to a monomeric functional unit. Simply combining functional units into a complex without cooperativity does not benefit from this advantageous feature. Heteromeric subunit combination offers further opportunities to yield a wider variety of channel types from limited genetic resource (4) while retaining the advantageous feature of cooperativity. The majority of ligand-gated ion channels have each subunit participating in ligand binding, a situation that would maximize the benefit of cooperativity. Intriguingly, many pentameric channels do not possess five ligand-binding sites. Acetylcholine receptors, for example, often have only two ligand-binding sites (26). In these cases, the ligand-bound sites are always separated across the channel protein complex, as if this arrangement would allow acetylcholine binding to exert the maximal energetic effect on channel activation.

SUPPORTING MATERIAL

Supporting material can be found online at https://doi.org/10.1016/j.bpj.2024.04.005.

AUTHOR CONTRIBUTIONS

Conceptualization, S.L. and J.Z.; patch clamp, S.L.; data analysis, S.L. and J.Z.; funding acquisition, J.Z.; supervision, J.Z.; writing – original draft, S.L. and J.Z.; writing – review & editing, S.L. and J.Z.

ACKNOWLEDGMENTS

We are grateful to Avi Priel for sharing the concatemer constructs in this study. We thank Marcel Goldschen-Ohm and members of the Zheng lab for helpful discussions. This study is supported by National Institutes of Health grant R01NS103954 (to J.Z.).

DECLARATION OF INTERESTS

The authors declare no competing interests.

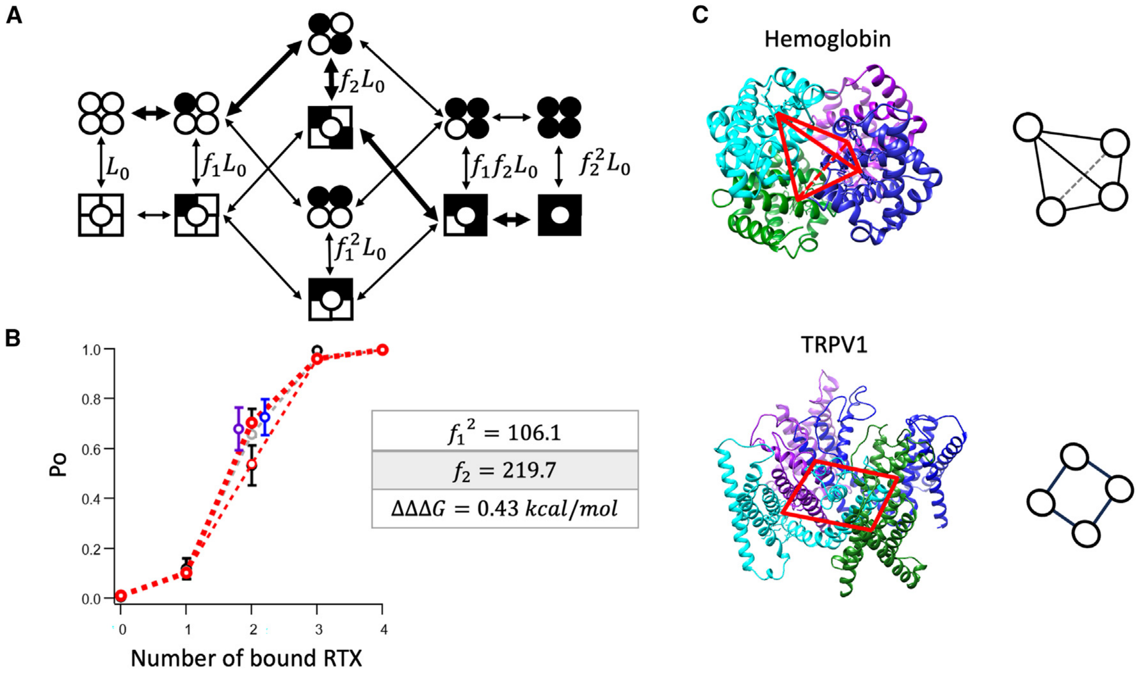

FIGURE 5 (*A*) Illustration of a modified MWC model with position effects. L_0 represents the equilibrium constant for the apo state, f_1 represents the cooperative factor reflecting the energy contribution by one ligand binding to the pair of diagonal binding sites; f_2 represents the cooperative factor reflecting the energy contribution by two ligands binding to the pair of diagonal binding sites; when there is no position effect, f_2 should equal to f_1^2, making the model equivalent to the classic MWC model. Thicker arrows indicate pathways with higher probabilities. (*B*) Global fitting results for the *Po* data from AAAA, YAAA, YYAA, YAYA, AYAY, YYYA, and YYYY concatemers, with the Y subunits loaded with RTX: $L_0 = 0.01$, $f_1 = 10.3$, $f_2 = 219.7$, which produce $\Delta\Delta\Delta G = 0.43$ kcal/mol between neighbor and kitty-corner positions, $n = 4$–10. Black circles represent the data points; red circles represent predictions of our modified MWC model; gray dashed lines represent a classic MWC model with $L_0 = 0.01$, $f = 15.7$. The purple circle represents the YAYA data point, and the blue circle represents the AYAY data point. Error bars represent SEM. (*C*) Relationships between ligand-binding pockets in hemoglobin (PDB: 2DHB, *top*) and tetrameric ligand-gated ion channels such as TRPV1 (transmembrane part of TRPV1, modified from PDB: 3J5R, *bottom*). Positions of ligand-binding sites are highlighted by red lines between them and illustrated by a diagram on the right.

REFERENCES

1. Hodgkin, A. L., and A. F. Huxley. 1952. A quantitative description of membrane current and its application to conduction and excitation in nerve. *J. Physiol.* 117:500–544. https://doi.org/10.1113/jphysiol.1952.sp004764.
2. Zagotta, W. N., T. Hoshi, and R. W. Aldrich. 1994. Shaker potassium channel gating. III: Evaluation of kinetic models for activation. *J. Gen. Physiol.* 103:321–362. https://doi.org/10.1085/jgp.103.2.321.
3. Schoppa, N. E., and F. J. Sigworth. 1998. Activation of Shaker potassium channels. III. An activation gating model for wild-type and V2 mutant channels. *J. Gen. Physiol.* 111:313–342. https://doi.org/10.1085/jgp.111.2.313.
4. Zheng, J., and M. C. Trudeau. 2023. *In* Textbook of Ion Channels: Three Volume Set. CRC Press, Boca Raton, FL.
5. Rothberg, B. S., and K. L. Magleby. 1998. Kinetic structure of large-conductance Ca2+-activated K+ channels suggests that the gating includes transitions through intermediate or secondary states. A mechanism for flickers. *J. Gen. Physiol.* 111:751–780. https://doi.org/10.1085/jgp.111.6.751.
6. Horrigan, F. T., and R. W. Aldrich. 1999. Allosteric voltage gating of potassium channels II. Mslo channel gating charge movement in the absence of Ca(2+). *J. Gen. Physiol.* 114:305–336.
7. Horrigan, F. T., J. Cui, and R. W. Aldrich. 1999. Allosteric voltage gating of potassium channels I. Mslo ionic currents in the absence of Ca(2+). *J. Gen. Physiol.* 114:277–304.
8. Horrigan, F. T., and R. W. Aldrich. 2002. Coupling between voltage sensor activation, Ca2+ binding and channel opening in large conductance (BK) potassium channels. *J. Gen. Physiol.* 120:267–305.
9. Bolton, W., and M. F. Perutz. 1970. Three Dimensional Fourier Synthesis of Horse Deoxyhaemoglobin at 2.8 Å Resolution. *Nature.* 228:551–552. https://doi.org/10.1038/228551a0.
10. Edsall, J. T. 1972. Blood and hemoglobin: The evolution of knowledge of functional adaptation in a biochemical system. *J. Hist. Biol.* 5:205–257. https://doi.org/10.1007/BF00346659.
11. Monod, J., J. Wyman, and J. P. Changeux. 1965. On the Nature of Allosteric Transitions: A Plausible Model. *J. Mol. Biol.* 12:88–118. https://doi.org/10.1016/s0022-2836(65)80285-6.
12. Koshland, D. E., Jr., G. Némethy, and D. Filmer. 1966. Comparison of experimental binding data and theoretical models in proteins containing subunits. *Biochemistry.* 5:365–385. https://doi.org/10.1021/bi00865a047.
13. Li, S., P. T. Nguyen, ..., J. Zheng. 2023. Opening of capsaicin receptor TRPV1 is Stabilized Equally by Its Four Subunits. *J. Biol. Chem.* 299, 104828.
14. Li, S., and J. Zheng. 2023. The Capsaicin Binding Affinity of Wild-Type and Mutant TRPV1 Ion Channels. *J. Biol. Chem.* 299, 105268. https://doi.org/10.1016/j.jbc.2023.105268.
15. Hazan, A., R. Kumar, ..., A. Priel. 2015. The pain receptor TRPV1 displays agonist-dependent activation stoichiometry. *Sci. Rep.* 5:12278.
16. Liao, M., E. Cao, ..., Y. Cheng. 2013. Structure of the TRPV1 ion channel determined by electron cryo-microscopy. *Nature.* 504:107–112.
17. Cao, E., M. Liao, ..., D. Julius. 2013. TRPV1 structures in distinct conformations reveal activation mechanisms. *Nature.* 504:113–118.

18. Yang, F., X. Xiao, …, J. Zheng. 2015. Structural mechanism underlying capsaicin binding and activation of the TRPV1 ion channel. *Nat. Chem. Biol.* 11:518–524. https://doi.org/10.1038/nchembio.1835.

19. Hazan, A., A. Basu, …, A. Priel. 2016. Tyrosine residue in the TRPV1 vanilloid binding pocket regulates deactivation kinetics. *J. Biol. Chem.* 291:13855–13863.

20. Yang, F., X. Xiao, …, J. Zheng. 2018. The conformational wave in capsaicin activation of transient receptor potential vanilloid 1 ion channel. *Nat. Commun.* 9:2879.

21. Zhang, K., D. Julius, and Y. Cheng. 2021. Structural snapshots of TRPV1 reveal mechanism of polymodal functionality. *Cell.* 184:5138–5150.e12.

22. Yang, F., X. Xiao, …, J. Zheng. 2015. Structural mechanism underlying capsaicin binding and activation of the TRPV1 ion channel. *Nat. Chem. Biol.* 11:518–524.

23. Yin, Y., Y. Dong, …, J. Zheng. 2019. Structural mechanisms underlying activation of TRPV1 channels by pungent compounds in gingers. *Br. J. Pharmacol.* 176:3364–3377.

24. Vu, S., V. Singh, …, J. Zheng. 2020. New capsaicin analogs as molecular rulers to define the permissive conformation of the mouse TRPV1 ligand-binding pocket. *Elife.* 9, e62039. https://doi.org/10.7554/eLife.62039.

25. Dong, Y., Y. Yin, …, J. Zheng. 2019. A distinct structural mechanism underlies TRPV1 activation by piperine. *Biochem. Biophys. Res. Commun.* 516:365–372. https://doi.org/10.1016/j.bbrc.2019.06.039.

26. Bouzat, C., and J. F. Chrestia. 2023. Acetylcholine Receptors. *In* Textbook of Ion Channels Volume II: Properties, Function, and Pharmacology of the Superfamilies, p. 223.

Biophysical Journal, Volume *123*

Supplemental information

How much does TRPV1 deviate from an ideal MWC-type protein?

Shisheng Li and Jie Zheng

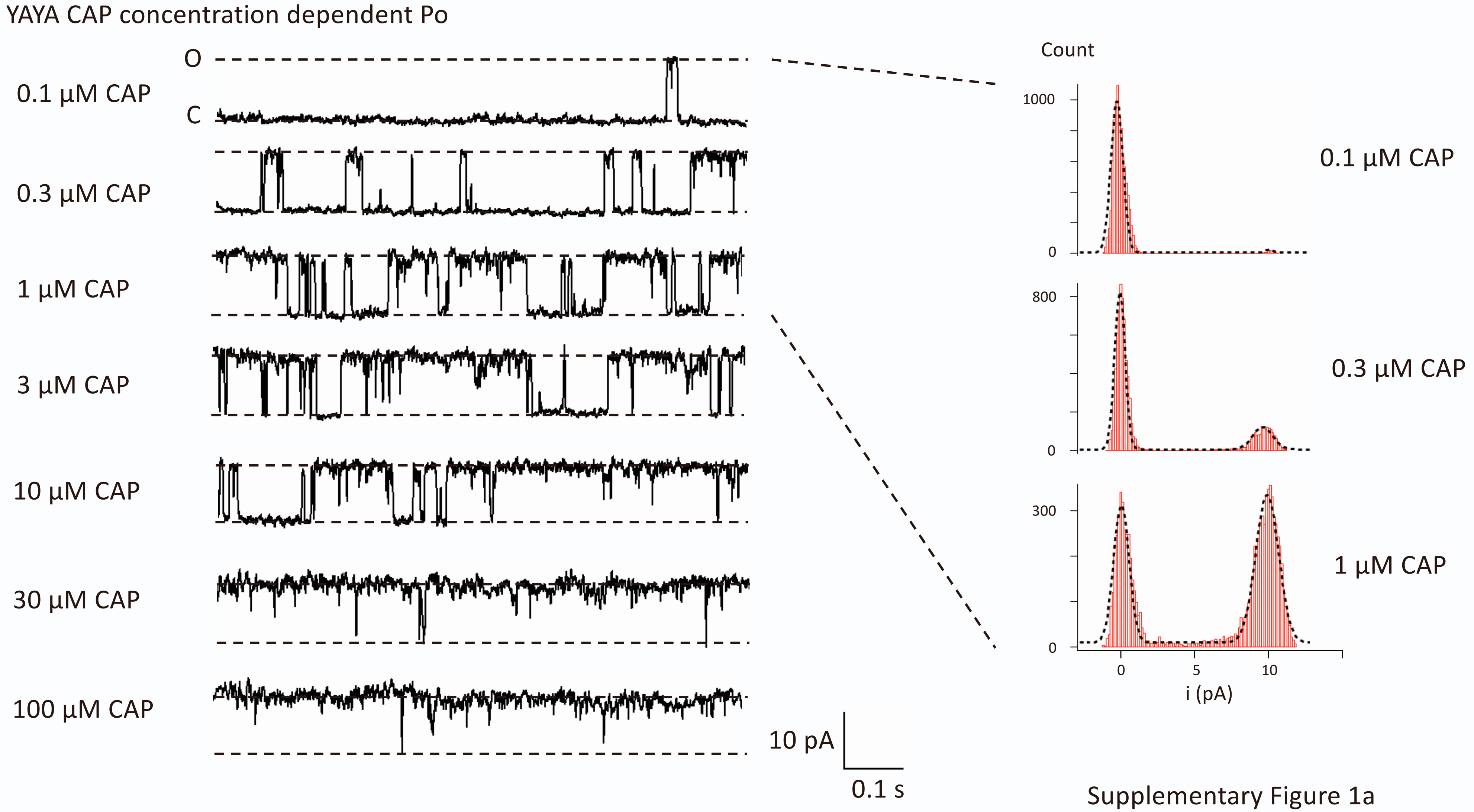

Supplementary Figure 1a

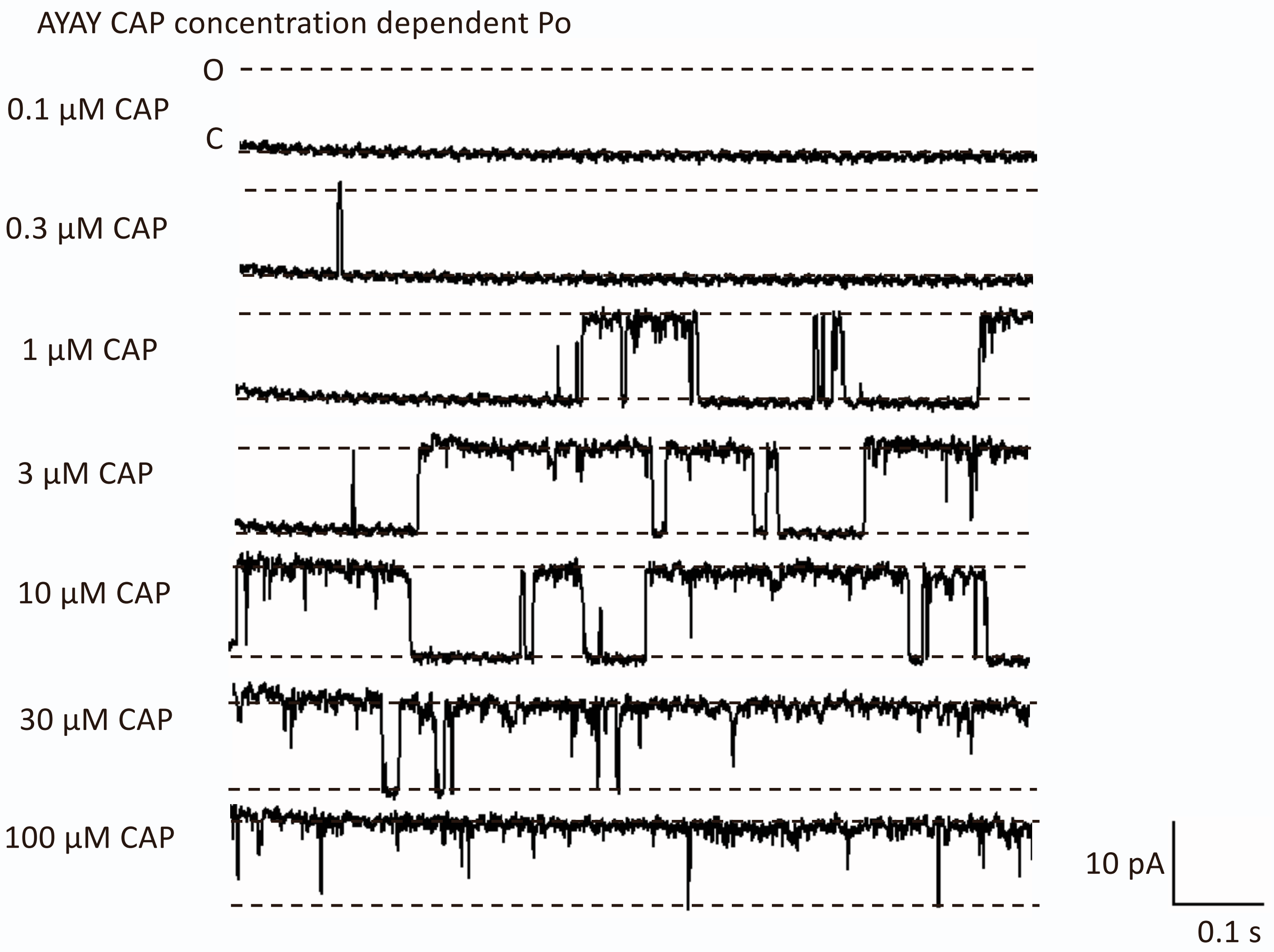

Supplementary Figure 1b

AYAY CAP concentration dependent Po loaded with 2 RTX molecules

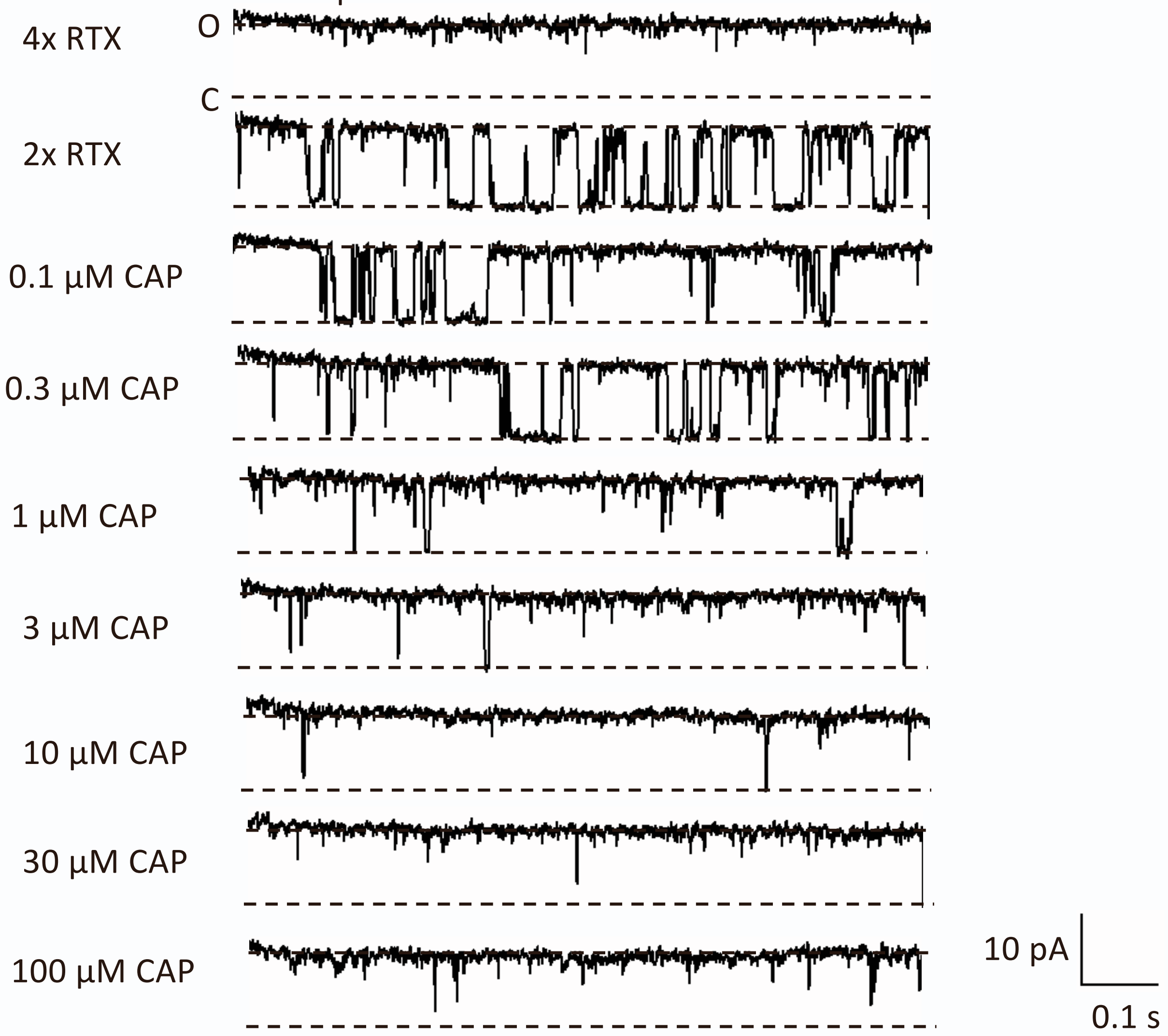

Supplementary Figure 2a

YAYA CAP concentration dependent Po loaded with 2 RTX molecules

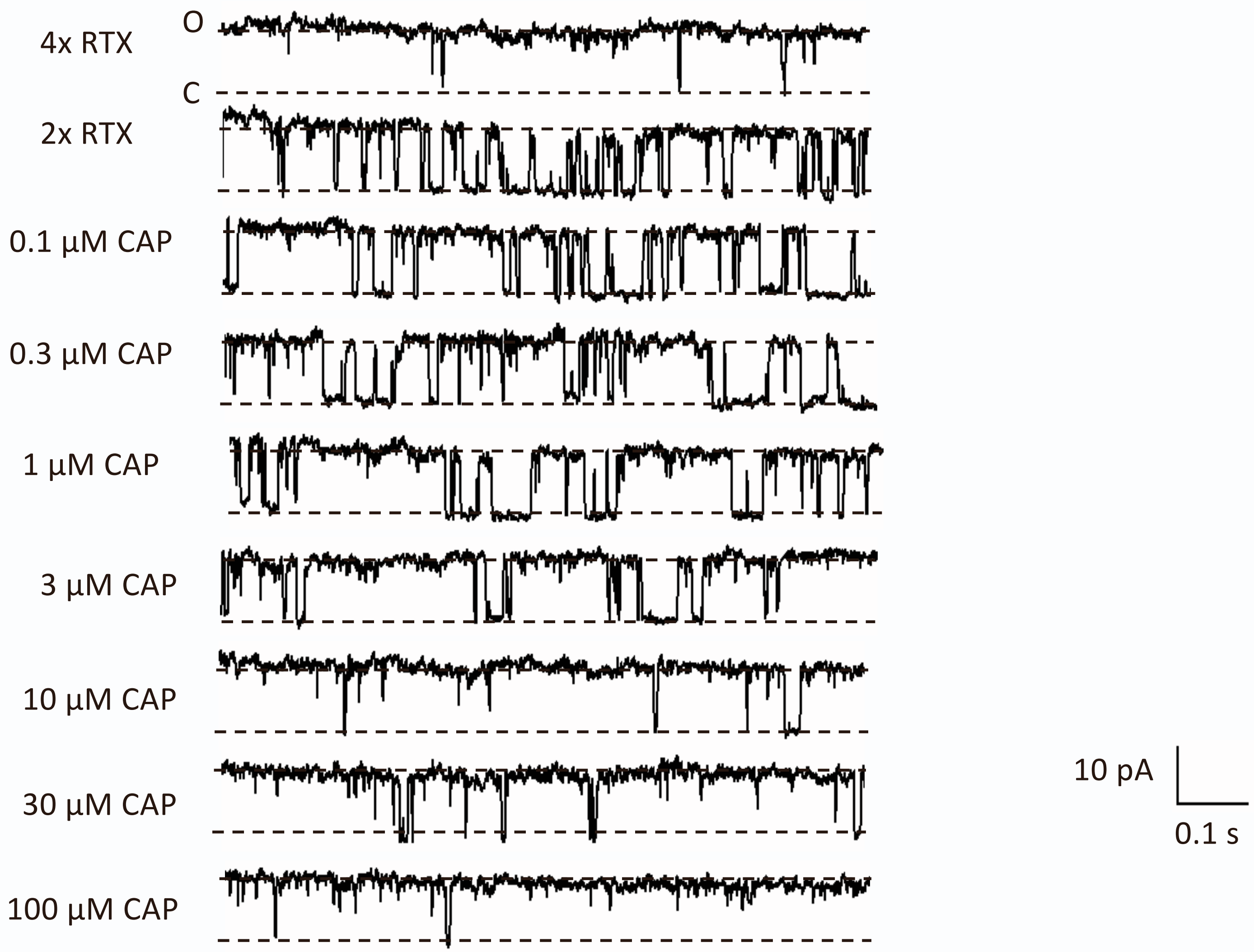

Supplementary Figure 2b

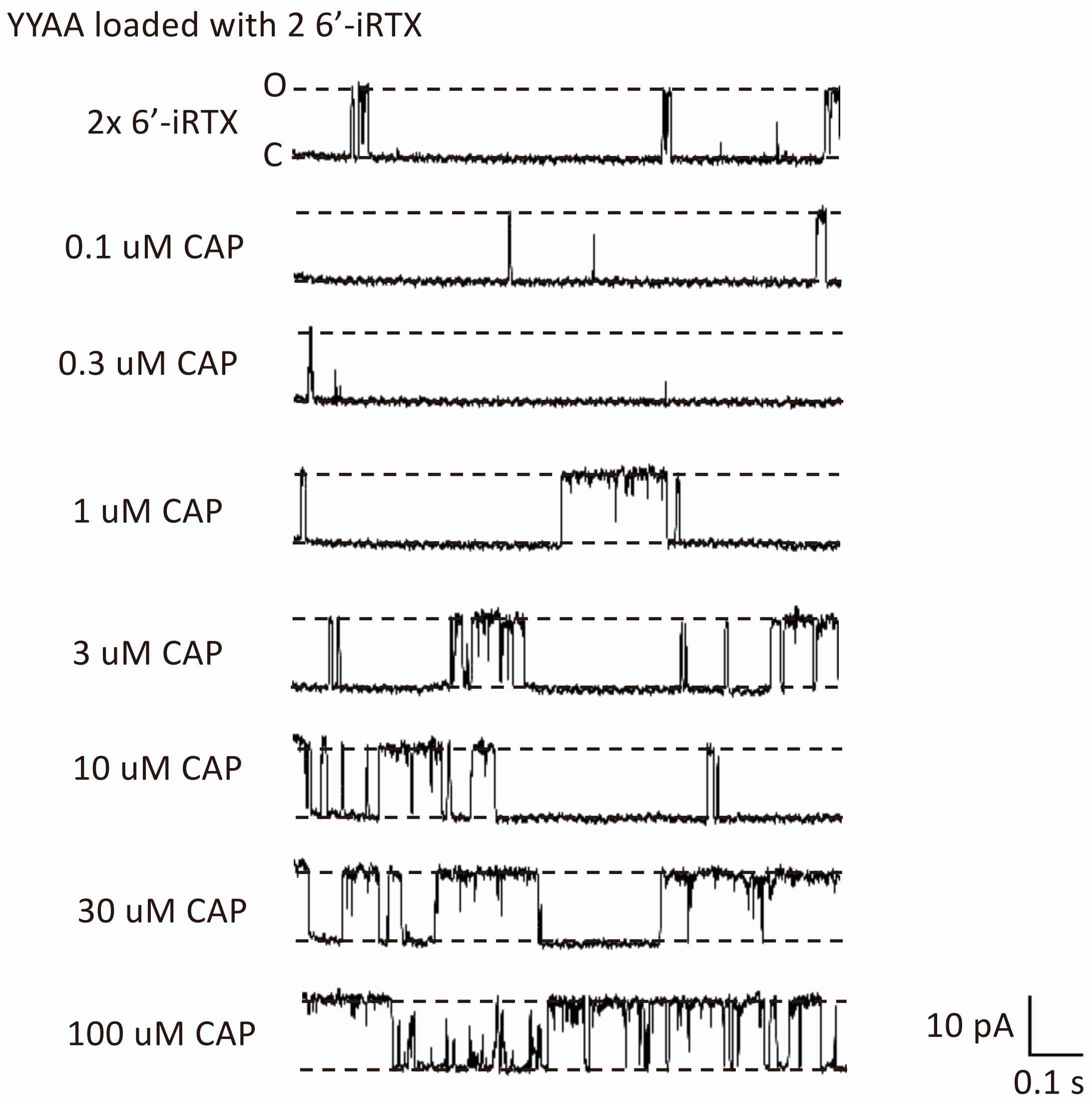

Supplementary Figure 3a

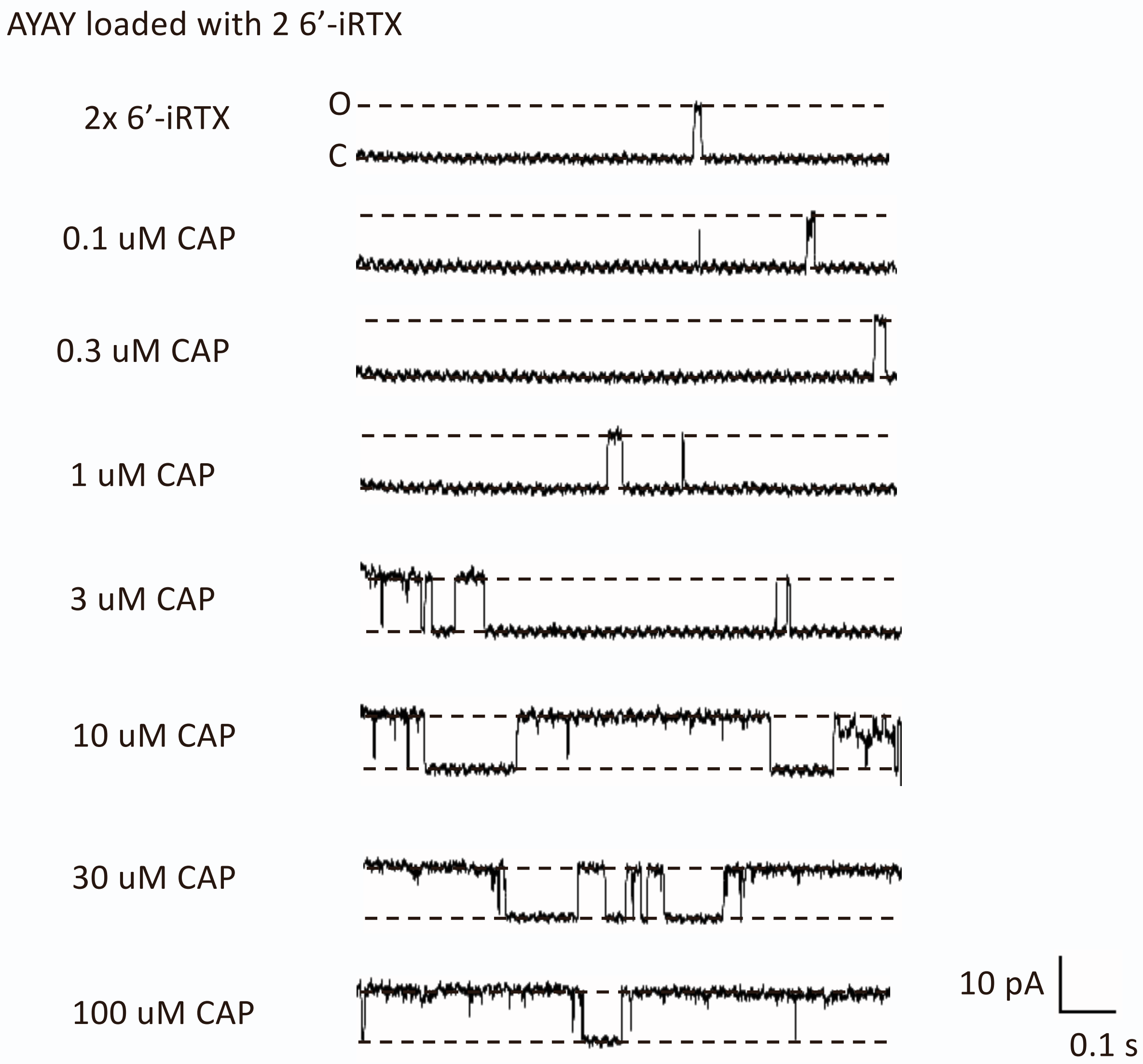

Supplementary Figure 3b

YAYA with 2x 6'-iRTX

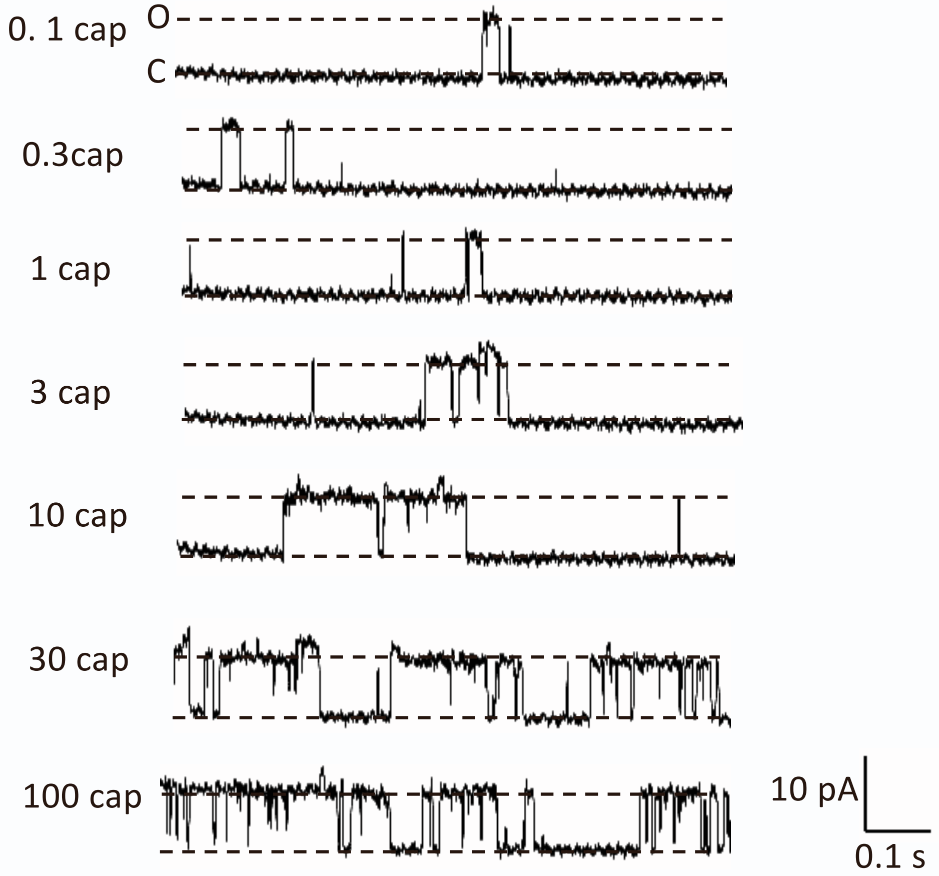

Supplementary Figure 3c

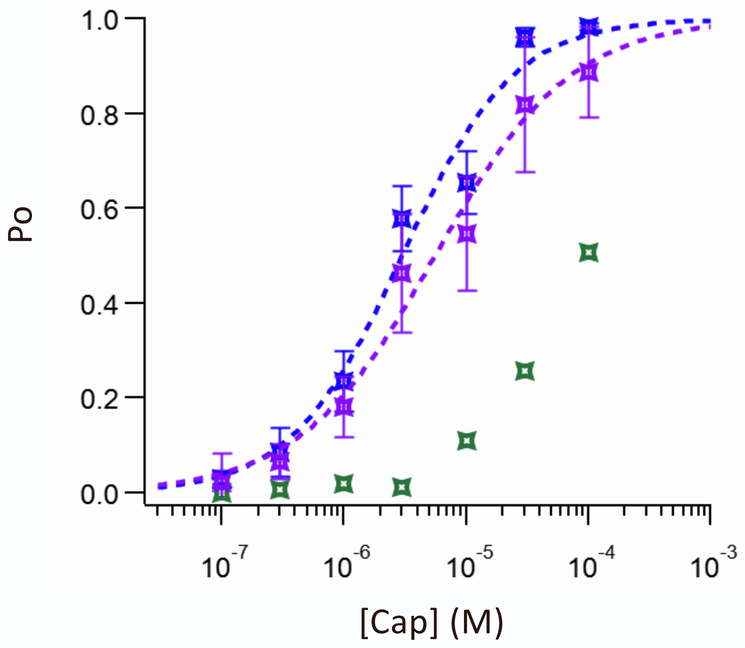

After excluding the outlier recording,
n = 4, EC50 2.99 μM, Hill slope 0.95

Before excluding the outlier recording,
n = 5, EC50 5.55 μM, Hill slope 0.95

One AYAY recording excluded as an outlier

Supplementary Figure 4

Comparison between classic MWC model with Position effect model in Figure 3 (left) and Figure 4 (right)

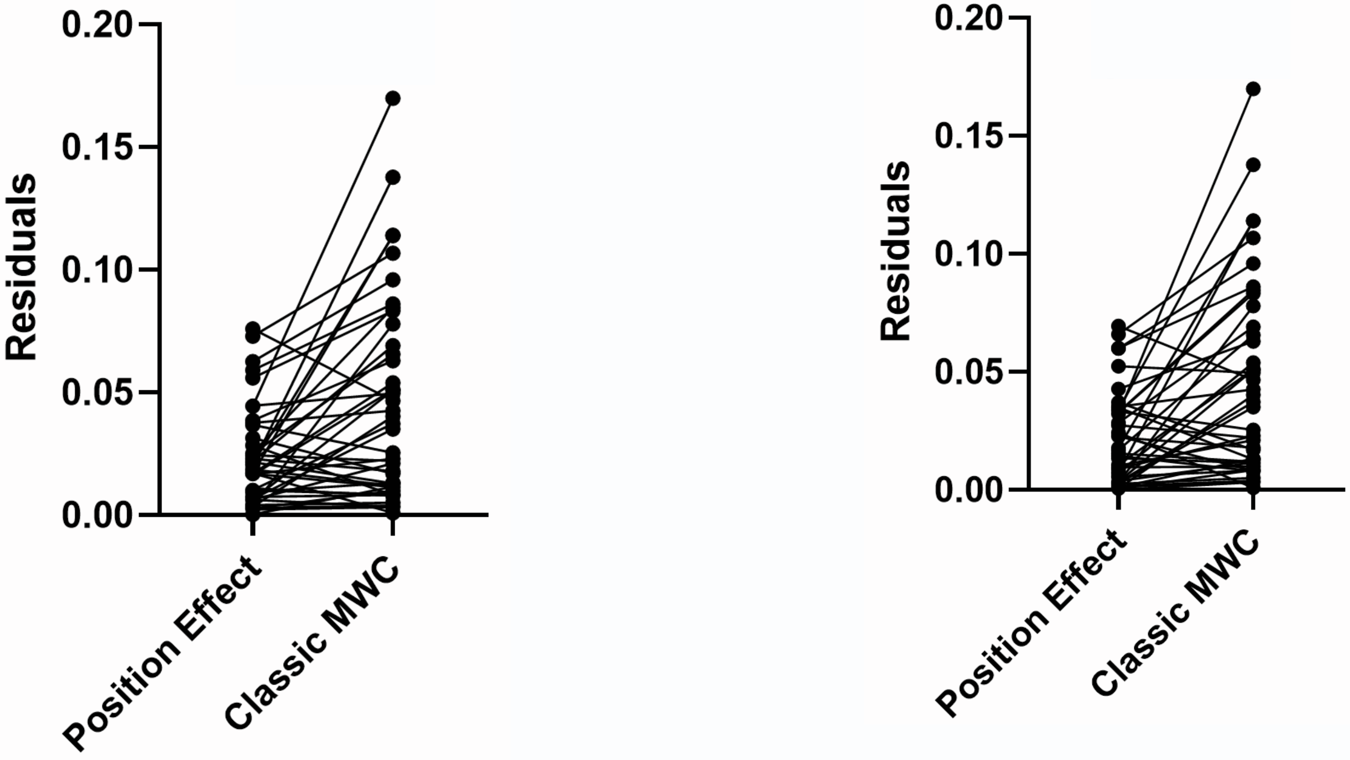

Supplementary Figure 5

Table Analyzed	Data in Figure 1B	AYAY vs YYAA						
Two-way ANOVA	Ordinary							
Alpha	0.05							
Source of Variation	% of total variation	P value	P value summary	Significant?				
Interaction	0.4622	0.4235	ns	No				
Row Factor	72.11	<0.0001	****	Yes				
Column Factor	0.783	0.0046	**	Yes				
ANOVA table	SS (Type III)	DF	MS	F (DFn, DFd)	P value			
Interaction	0.0641	5	0.01282	F (5, 84) = 0.9990	P=0.4235			
Row Factor	10	5	2	F (5, 84) = 155.9	P<0.0001			
Column Factor	0.1086	1	0.1086	F (1, 84) = 8.462	P=0.0046			
Residual	1.078	84	0.01283					
Difference between column means								
Predicted (LS) mean of AYAY	0.4917							
Predicted (LS) mean of YYAA	0.4141							
Difference between predicted means	0.07767							
SE of difference	0.0267							
95% CI of difference	0.02457 to 0.1308							
Compare each cell mean with the other cell mean in that row								
Number of families	1							
Number of comparisons per family	6							
Alpha	0.05							
Sidak's multiple comparisons test	Mean Diff.	95.00% CI of diff.	Significant?	Summary	usted P Value			
AYAY - YYAA								
1e-07	-0.0004735	-0.1767 to 0.1758	No	ns	>0.9999			
3e-07	0.03142	-0.1448 to 0.2076	No	ns	0.9975			
1e-06	0.08885	-0.08738 to 0.2651	No	ns	0.6914			
1e-05	0.08393	-0.09230 to 0.2602	No	ns	0.7436			
3e-05	0.1913	0.01508 to 0.3675	Yes	*	0.0263			
0.0001	0.07101	-0.1052 to 0.2472	No	ns	0.8615			
Test details	Mean 1	Mean 2	Mean Diff.	SE of diff.	N1	N2	t	DF
AYAY - YYAA								
1e-07	0.02933	0.0298	-0.0004735	0.0654	1	1	0.007239	84
3e-07	0.08599	0.05457	0.03142	0.0654	1	1	0.4804	84
1e-06	0.2365	0.1477	0.08885	0.0654	1	1	1.358	84
1e-05	0.6555	0.5716	0.08393	0.0654	1	1	1.283	84
3e-05	0.9599	0.7686	0.1913	0.0654	1	1	2.925	84
0.0001	0.9832	0.9122	0.07101	0.0654	1	1	1.086	84

Table Analyzed	Data in Figure 1B	YAYA vs YYAA						
Two-way ANOVA	Ordinary							
Alpha	0.05							
Source of Variation	% of total variation	P value	P value summary	Significant?				
Interaction	0.8131	0.1608	ns	No				
Row Factor	76.16	<0.0001	****	Yes				
Column Factor	1.951	<0.0001	****	Yes				
ANOVA table	SS (Type III)	DF	MS	F (DFn, DFd)	P value			
Interaction	0.123	5	0.0246	F (5, 90) = 1.628	P=0.1608			
Row Factor	11.52	5	2.305	F (5, 90) = 152.4	P<0.0001			
Column Factor	0.2951	1	0.2951	F (1, 90) = 19.52	P<0.0001			
Residual	1.361	90	0.01512					
Difference between column means								
Predicted (LS) mean of YAYA	0.5321							
Predicted (LS) mean of YYAA	0.4141							
Difference between predicted means	0.1181							
SE of difference	0.02672							
95% CI of difference	0.06497 to 0.1711							
Compare each cell mean with the other cell mean in that row								
Number of families	1							
Number of comparisons per family	6							
Alpha	0.05							
Sidak's multiple comparisons test	Mean Diff.	95.00% CI of diff.	Significant?	Summary	Adjusted P Value			
YAYA - YYAA								
1e-07	-0.01221	-0.1883 to 0.1639	No	ns	>0.9999			
3e-07	0.1201	-0.05599 to 0.2961	No	ns	0.3524			
1e-06	0.1271	-0.04901 to 0.3031	No	ns	0.2894			
1e-05	0.2245	0.04846 to 0.4006	Yes	**	0.0055			
3e-05	0.1809	0.004876 to 0.3570	Yes	*	0.0408			
0.0001	0.06793	-0.1081 to 0.2440	No	ns	0.8845			
Test details	Mean 1	Mean 2	Mean Diff.	SE of diff.	N1	N2	t	DF
YAYA - YYAA								
1e-07	0.0176	0.0298	-0.01221	0.06545	1	1	0.1865	90
3e-07	0.1746	0.05457	0.1201	0.06545	1	1	1.835	90
1e-06	0.2747	0.1477	0.1271	0.06545	1	1	1.941	90
1e-05	0.7961	0.5716	0.2245	0.06545	1	1	3.431	90
3e-05	0.9495	0.7686	0.1809	0.06545	1	1	2.765	90
0.0001	0.9801	0.9122	0.06793	0.06545	1	1	1.038	90

Table Analyzed	Data in Figure 1C	YYAA vs YAYA						
Two-way ANOVA	Ordinary							
Alpha	0.05							
Source of Variation	% of total variation	P value	P value summary	Significant?				
Interaction	1.259	0.9387	ns	No				
Row Factor	47.99	<0.0001	****	Yes				
Column Factor	2.046	0.1621	ns	No				
ANOVA table	SS	DF	MS	F (DFn, DFd)	P value			
Interaction	0.04894	5	0.009787	F (5, 48) = 0.2481	P=0.9387			
Row Factor	1.866	5	0.3731	F (5, 48) = 9.459	P<0.0001			
Column Factor	0.07953	1	0.07953	F (1, 48) = 2.016	P=0.1621			
Residual	1.893	48	0.03945					
Difference between column means								
Mean of YYAA	0.757							
Mean of YAYA	0.8298							
Difference between means	-0.07281							
SE of difference	0.05128							
95% CI of difference	-0.1759 to 0.03029							
Compare each cell mean with the other cell mean in that row								
Number of families	1							
Number of comparisons per family	6							
Alpha	0.05							
Sidak's multiple comparisons test	Mean Diff.	95.00% CI of diff.	Significant?	Summary	djusted P Value			
YYAA - YAYA								
1e-07	-0.09134	-0.4360 to 0.2533	No	ns	0.978			
3e-07	-0.09337	-0.4380 to 0.2513	No	ns	0.9755			
1e-06	-0.168	-0.5126 to 0.1767	No	ns	0.7123			
1e-05	-0.07544	-0.4201 to 0.2692	No	ns	0.9918			
3e-05	0.009542	-0.3351 to 0.3542	No	ns	>0.9999			
0.0001	-0.01834	-0.3630 to 0.3263	No	ns	>0.9999			
Test details	Mean 1	Mean 2	Mean Diff.	SE of diff.	N1	N2	t	DF
YYAA - YAYA								
1e-07	0.5125	0.6038	-0.09134	0.1256	1	1	0.7271	48
3e-07	0.568	0.6614	-0.09337	0.1256	1	1	0.7433	48
1e-06	0.614	0.782	-0.168	0.1256	1	1	1.337	48
1e-05	0.8753	0.9508	-0.07544	0.1256	1	1	0.6006	48
3e-05	0.9956	0.986	0.009542	0.1256	1	1	0.07597	48
0.0001	0.9763	0.9947	-0.01834	0.1256	1	1	0.146	48

Table Analyzed	Data in Figure 1C	YYAA vs AYAY						
Two-way ANOVA	Ordinary							
Alpha	0.05							
Source of Variation	% of total variation	P value	P value summary	Significant?				
Interaction	2.077	0.8164	ns	No				
Row Factor	50.84	<0.0001	****	Yes				
Column Factor	2.035	0.1474	ns	No				
ANOVA table	SS	DF	MS	F (DFn, DFd)	P value			
Interaction	0.07828	5	0.01566	F (5, 48) = 0.4427	P=0.8164			
Row Factor	1.916	5	0.3832	F (5, 48) = 10.84	P<0.0001			
Column Factor	0.0767	1	0.0767	F (1, 48) = 2.169	P=0.1474			
Residual	1.698	48	0.03537					
Difference between column means								
Mean of YYAA	0.757							
Mean of AYAY	0.8285							
Difference between means	-0.07151							
SE of difference	0.04856							
95% CI of difference	-0.1691 to 0.02612							
Compare each cell mean with the other cell mean in that row								
Number of families	1							
Number of comparisons per family	6							
Alpha	0.05							
Sidak's multiple comparisons test	Mean Diff.	95.00% CI of diff.	Significant?	Summary	Adjusted P Value			
YYAA - AYAY								
1e-07	-0.07395	-0.4003 to 0.2524	No	ns	0.9902			
3e-07	-0.06044	-0.3868 to 0.2659	No	ns	0.9967			
1e-06	-0.2209	-0.5473 to 0.1054	No	ns	0.3505			
1e-05	-0.06127	-0.3876 to 0.2651	No	ns	0.9964			
3e-05	0.004623	-0.3217 to 0.3310	No	ns	>0.9999			
0.0001	-0.01709	-0.3434 to 0.3093	No	ns	>0.9999			
Test details	Mean 1	Mean 2	Mean Diff.	SE of diff.	N1	N2	t	DF
YYAA - AYAY								
1e-07	0.5125	0.5865	-0.07395	0.1189	1	1	0.6218	48
3e-07	0.568	0.6284	-0.06044	0.1189	1	1	0.5082	48
1e-06	0.614	0.8349	-0.2209	0.1189	1	1	1.857	48
1e-05	0.8753	0.9366	-0.06127	0.1189	1	1	0.5152	48
3e-05	0.9956	0.9909	0.004623	0.1189	1	1	0.03887	48
0.0001	0.9763	0.9934	-0.01709	0.1189	1	1	0.1437	48

Table Analyzed	Data in Figure 1D	YYAA vs AYAY						
Two-way ANOVA	Ordinary							
Alpha	0.05							
Source of Variation	% of total variation	P value	P value summary	Significant?				
Interaction	3.297	0.1352	ns	No				
Row Factor	67.6	<0.0001	****	Yes				
Column Factor	2.022	0.015	*	Yes				
ANOVA table	SS (Type III)	DF	MS	F (DFn, DFd)	P value			
Interaction	0.146	6	0.02434	F (6, 56) = 1.712	P=0.1352			
Row Factor	2.994	6	0.499	F (6, 56) = 35.10	P<0.0001			
Column Factor	0.08958	1	0.08958	F (1, 56) = 6.300	P=0.0150			
Residual	0.7962	56	0.01422					
Difference between column means								
Predicted (LS) mean of YYAA	0.1512							
Predicted (LS) mean of AYAY	0.2242							
Difference between predicted means	-0.07302							
SE of difference	0.02909							
95% CI of difference	-0.1313 to -0.01474							
Compare each cell mean with the other cell mean in that row								
Number of families	1							
Number of comparisons per family	7							
Alpha	0.05							
Sidak's multiple comparisons test	Mean Diff.	95.00% CI of diff.	Significant?	Summary	Adjusted P Value			
YYAA - AYAY								
1e-07	0.001485	-0.2128 to 0.2158	No	ns	>0.9999			
3e-07	0.008467	-0.2059 to 0.2228	No	ns	>0.9999			
1e-06	0.01694	-0.1974 to 0.2313	No	ns	>0.9999			
3e-06	-0.02924	-0.2436 to 0.1851	No	ns	0.9998			
1e-05	-0.09114	-0.3055 to 0.1232	No	ns	0.8554			
3e-05	-0.182	-0.3964 to 0.03229	No	ns	0.1413			
0.0001	-0.2356	-0.4499 to -0.02131	Yes	*	0.0234			
Test details	Mean 1	Mean 2	Mean Diff.	SE of diff.	N1	N2	t	DF
YYAA - AYAY								
1e-07	0.009782	0.008297	0.001485	0.07697	1	1	0.01929	56
3e-07	0.02053	0.01206	0.008467	0.07697	1	1	0.11	56
1e-06	0.03171	0.01477	0.01694	0.07697	1	1	0.2201	56
3e-06	0.05741	0.08665	-0.02924	0.07697	1	1	0.3799	56
1e-05	0.128	0.2191	-0.09114	0.07697	1	1	1.184	56
3e-05	0.3838	0.5659	-0.182	0.07697	1	1	2.365	56
0.0001	0.427	0.6626	-0.2356	0.07697	1	1	3.061	56

Table Analyzed	Data in Figure 1D	YYAA vs AYAY						
Two-way ANOVA	Ordinary							
Alpha	0.05							
Source of Variation	% of total variation	P value	P value summary	Significant?				
Interaction	2.139	0.5126	ns	No				
Row Factor	51.19	<0.0001	****	Yes				
Column Factor	4.822	0.0009	***	Yes				
ANOVA table	SS (Type III)	DF	MS	F (DFn, DFd)	P value			
Interaction	0.1247	6	0.02079	F (6, 70) = 0.8822	P=0.5126			
Row Factor	2.985	6	0.4976	F (6, 70) = 21.12	P<0.0001			
Column Factor	0.2813	1	0.2813	F (1, 70) = 11.94	P=0.0009			
Residual	1.649	70	0.02356					
Difference between column means								
Predicted (LS) mean of YYAA	0.1512							
Predicted (LS) mean of YAYA	0.2739							
Difference between predicted means	-0.1227							
SE of difference	0.03553							
95% CI of difference	-0.1936 to -0.05189							
Compare each cell mean with the other cell mean in that row								
Number of families	1							
Number of comparisons per family	7							
Alpha	0.05							
Sidak's multiple comparisons test	Mean Diff.	95.00% CI of diff.	Significant?	Summary	Adjusted P Value			
YYAA - YAYA								
1e-07	-0.02165	-0.2814 to 0.2381	No	ns	>0.9999			
3e-07	-0.02446	-0.2842 to 0.2353	No	ns	>0.9999			
1e-06	-0.07143	-0.3312 to 0.1883	No	ns	0.9847			
3e-06	-0.1064	-0.3662 to 0.1534	No	ns	0.8802			
1e-05	-0.2157	-0.4755 to 0.04404	No	ns	0.1608			
3e-05	-0.2	-0.4598 to 0.05974	No	ns	0.2312			
0.0001	-0.2195	-0.4793 to 0.04023	No	ns	0.1465			
Test details	Mean 1	Mean 2	Mean Diff.	SE of diff.	N1	N2	t	DF
YYAA - YAYA								
1e-07	0.009782	0.03143	-0.02165	0.094	1	1	0.2303	70
3e-07	0.02053	0.04498	-0.02446	0.094	1	1	0.2602	70
1e-06	0.03171	0.1031	-0.07143	0.094	1	1	0.7599	70
3e-06	0.05741	0.1638	-0.1064	0.094	1	1	1.132	70
1e-05	0.128	0.3437	-0.2157	0.094	1	1	2.295	70
3e-05	0.3838	0.5839	-0.2	0.094	1	1	2.128	70
0.0001	0.427	0.6465	-0.2195	0.094	1	1	2.336	70

Column B	Classic MWC	
vs.	vs.	
Column A	Position Effect	
Wilcoxon matched-pairs signed rank test		
P value		0.0006
Exact or approximate P value?	Exact	
P value summary	***	
Significantly different (P < 0.05)?	Yes	
One- or two-tailed P value?	Two-tailed	
Sum of positive, negative ranks	750.0 , -196.0	
Sum of signed ranks (W)		554
Number of pairs		43
Number of ties (ignored)		0
Median of differences		
Median		0.007248
How effective was the pairing?		
rs (Spearman)		0.5447
P value (one tailed)	<0.0001	
P value summary	****	
Was the pairing significantly effective?	Yes	

Column B	Classic MWC	
vs.	vs.	
Column A	Position Effect	
Wilcoxon matched-pairs signed rank test		
P value		0.0002
Exact or approximate P value?	Exact	
P value summary	***	
Significantly different (P < 0.05)?	Yes	
One- or two-tailed P value?	Two-tailed	
Sum of positive, negative ranks	767.0 , -179.0	
Sum of signed ranks (W)		588
Number of pairs		43
Number of ties (ignored)		0
Median of differences		
Median		0.01077
How effective was the pairing?		
rs (Spearman)		0.4773
P value (one tailed)		0.0006
P value summary	***	
Was the pairing significantly effective?	Yes	

Figure 1B					
	EC50 (mean ± SD)	Hill slope (mean ± SD)	n	Max	Base
YYAA	7.31 ±0.13 µM	0.87 ± 0.01	12	1 (forced)	0 (forced)
YAYA	2.42 ± 0.22 µM	0.97 ± 0.08	5	1 (forced)	0 (forced)
AYAY	2.99 ± 0.53 µM	0.95 ± 0.14	4	1 (forced)	0 (forced)
Figure 1C					
	EC50 (mean ± SD)	Hill slope (mean ± SD)	n		
YYAA	2.42 ±0.65 µM	0.93 ± 0.15	5	1 (forced)	0.47
YAYA	1.13 ±0.07 µM	0.95±0.04	5	1 (forced)	0.57
AYAY	0.97 ± 0.28 µM	0.94 ± 0.20	5	1 (forced)	0.55
Figure 1D					
	EC50 (mean ± SD)	Hill slope (mean ± SD)	n		
YYAA	14.77 ± 1.59 µM	2.72 ± 0.55	4	0.03	0.43
YAYA	10.25 ± 1.82 µM	1.24 ± 0.24	8	0.04	0.7
AYAY	14.61 ± 1.65 µM	1.95 ± 0.35	6	0.02	0.68

Figure 3C						
	a	a'	L (with 6'-iRTX)	L (with RTX)	K	f
YYAA	0.83		0.01	0.92		
YAYA		1.37	0.03	1.38	$4.75 \times 10^4\ M^{-1}$	10.35
AYAY			0.02	1.35		
Classic MWC			0.02	1.16	5.83x104 M-1	10.42

Figure 4C						
	b	b'	L (with 6'-iRTX)	L (with RTX)	K	f
YYAA	1.07		0.02	0.94		
YAYA		2.18	0.02	1.32	$4.61 \times 10^4\ M^{-1}$	7.77
AYAY			0.02	1.28		
Classic MWC			0.02	1.16	5.83x104 M-1	10.42

	Figure 5	
MWC model	f	15.69
	L	0.01
2f model	f1	10.32
	f2	219.68
	L	0.01

YYAA CAP resposne

cap concentration / M	3E-08	1E-07	3E-07	0.000001	0.00001	0.00003	0.0001
	0.00116	0.00132	0.001683	0.075688	0.432355	0.797662	0.67483
	0.01829	0.042395	0.046242	0.101097	0.609344	0.738218	0.909935
	0.02246	0.055506	0.121504	0.20198	0.890999	0.803195	0.989712
	0.00608	0.042125	0.089885	0.018502	0.323397	0.43893	0.91552
	0.0025	0.007667	0.023282	0.36198	0.466941	0.634538	0.95577
		0.002211	0.003331	0.132323	0.607494	0.990124	0.965974
Po		0.004194	0.121493	0.065026	0.680081	0.903366	0.871221
		0.000705	0.029118	0.217594	0.457859	0.791083	0.980562
				0.036775	0.6211	0.834577	0.946127
				0.013542	0.381746	0.813236	
				0.365963	0.729234	0.443383	
				0.181382	0.658433	0.878361	
						0.924557	

YAYA CAP resposne

cap concentration / M	1E-07	3E-07	0.000001	0.000003	0.00001	0.00003	0.0001
	0.001465	0.004298	0.118171	0.504027	0.939473	0.996068	0.992251
	0.003498	0.123485	0.225567	0.422636	0.61102	0.941844	0.980643
Po	0.022952	0.225238	0.218257	0.798635	0.802688	0.997809	0.996706
	0.017854	0.02253	0.197567	0.248744	0.691452	0.81919	0.935415
	0.042208	0.497677	0.613994	0.745865	0.935903	0.992571	0.995534

AYAY CAP resposne

cap concentration / M	1E-07	3E-07	0.000001	0.000003	0.00001	0.00003	0.0001
	0.061226	0.180894	0.158339	0.669647	0.526443	0.973789	0.99663413
	0.023823	0.070584	0.362317	0.501933	0.617259	0.967113	0.978368625
Po	0.002939	0.006483	0.188846	0.426041	0.63376	0.902459	0.968404667
	0.000337	0.006471	0.020092	0.011191	0.110386	0.257236	0.507972924
	0.325531			0.711391	0.844575	0.996079	0.989365964

YYAA with RTX

cap concentration / M	cell 1	cell 2	cell 3	cell 4	cell 5
0	0.49842	0.487785	0.107985		0.716231
0.0000001	0.71584	0.474795	0.108695		0.750652
0.0000003	0.87048	0.439731	0.114172		0.84759
0.000001	0.89901	0.578636	0.201721		0.776695
0.00001	0.97072	0.932182	0.696173	0.801901	0.975632
0.00003	0.99738	0.993018		0.993072	0.998747
0.0001	0.99824	0.987928	0.982719	0.913617	0.99914

YAYA with RTX

cap concentration / M	cell 1	cell 2	cell 3	cell 4	cell 5
0	0.604121	0.189327	0.710825	0.937438	0.420273
0.0000001	0.656735	0.19536	0.710615	0.962873	0.493571
0.0000003	0.701432	0.426661	0.638907	0.969501	0.570313
0.000001	0.824363	0.730462	0.766204	0.985209	0.603599
0.000003	0.879815	0.777065	0.901907	0.972733	0.814442
0.00001	0.992118	0.818747	0.970062	0.996627	0.976245
0.00003	0.958433	0.992473	0.989679	0.998762	0.99071
0.0001	0.99039	0.995668	0.993592	0.998639	0.995032

AYAY with RTX

cap concentration / M	cell 1	cell 2	cell 3	cell 4	cell 5
0	0.691845	0.595336	0.767823	0.432273	0.327849
0.0000001	0.691887	0.60989	0.856322	0.425728	0.348426
0.0000003	0.695947	0.778167	0.608603	0.344579	0.714895
0.000001	0.781502	0.903207	0.966311	0.687934	0.835708
0.000003	0.869644	0.942702	0.974333	0.620049	0.858977
0.00001	0.960938	0.973929	0.975305	0.790927	0.981876
0.00003	0.998152	0.995628	0.997016	0.964571	0.999286
0.0001	0.99361	0.99692	0.997568	0.979565	0.999433

YYAA with 6'-iRTX

cap concentration / M	cell 1	cell 2	cell 3	cell 4	cell 5
0	0.00873	0.003999	0.02539	0.003108	0.001869
0.0000001	0.00246	0.020606	0.019061	0.005774	0.001012
0.0000003	0.00366	0.065908	0.016794	0.013435	0.002834
0.000001	0.01256	0.032805	0.054527	0.053341	0.005295
0.000003	0.05426	0.081571	0.090922		0.002879
0.00001	0.07083	0.123822	0.346912	0.05259	0.045906
0.00003	0.60895	0.403649	0.617971	0.095472	0.193125
0.0001	0.70361	0.457732	0.716091	0.132061	0.12537

YAYA with 6'-iRTX

cap concentration / M	cell 1	cell 2	cell 3	cell 4	cell 5	cell 6	cell 7	cell 8
0.0000001		0.042031	0.012028	0.025366		0.070854	0.003733	0.034557
0.0000003		0.049477	0.063847	0.018594		0.079319	0.002025	0.05663
0.000001		0.137754	0.053195	0.017201		0.029444	0.139807	0.241431
0.000003		0.108648	0.039225	0.11682		0.209297		0.345075
0.00001		0.158245	0.219271	0.188648	0.125988	0.370029	0.801439	0.542602
0.00003	0.521419	0.623337	0.202128	0.505087		0.694698		0.956524
0.0001	0.499784	0.619909	0.657618	0.581853	0.543887	0.645306		0.97726

AYAY with 6'-iRTX

cap concentration / M	cell 1	cell 2	cell 3	cell 4	cell 5	cell 6	cell 7	
0.0000001	0.016318		0.023014	0.000595	0.0007	0.007667		0.001485884
0.0000003	0.033404		0.002062	0.00071	0.001072	0.013476		0.021630435
0.000001	0.029399		0.011638	0.008332	0.003058	0.012434		0.02374756
0.000003	0.082403		0.023916	0.058506	0.067935	0.189653		0.097502979
0.00001	0.108437	0.394773	0.064238	0.206713	0.033504	0.172927		0.553439599
0.00003	0.350716	0.521583	0.65554	0.479749	0.465104	0.617938		0.870441125
0.0001	0.637644	0.62104	0.736006	0.490889	0.351791	0.809765		0.991088894

AAAA	YAAA	YYAA	AYAY	YAYA	YYYA	YYYY
0.003079	0.052774	0.453602	0.691845	0.604121	0.989044	0.9984
0.005536	0.232957	0.724096	0.595336	0.189327	0.998392	0.998152
0.002638	0.131032	0.4369	0.767823	0.710825	0.998437	0.996802
0.002092	0.063132	0.848706	0.432273	0.937438	0.9907	0.996618
0.007779		0.107985	0.327849	0.420273	0.993483	
		0.498422	0.966859	0.693723		
		0.487785	0.951765	0.945481		
		0.716231	0.929844	0.992585		
			0.690101	0.877653		
			0.921346	0.436193		
